# Antisense transcription is associated with expression of metal resistance determinants in *Cupriavidus metallidurans* CH34

**DOI:** 10.1093/mtomcs/mfae057

**Published:** 2024-11-19

**Authors:** Cornelia Große, Jan Grau, Martin Herzberg, Dietrich H Nies

**Affiliations:** Molecular Microbiology, Martin-Luther-University Halle-Wittenberg, 06120 Halle (Saale), Germany; Computer Sciences, Martin-Luther-University Halle-Wittenberg, 06120 Halle (Saale), Germany; Molecular Microbiology, Martin-Luther-University Halle-Wittenberg, 06120 Halle (Saale), Germany; Molecular Microbiology, Martin-Luther-University Halle-Wittenberg, 06120 Halle (Saale), Germany

## Abstract

*Cupriavidus metallidurans* is able to thrive in metal-rich environments but also survives metal starvation. Expression of metal resistance determinants in *C. metallidurans* was investigated on a global scale. *Cupriavidus metallidurans* was challenged with a MultiTox metal mix specifically designed for the wildtype strain CH34 and its plasmid-free derivative AE104, including treatment with ethylenediamintetraacetate (EDTA), or without challenge. The sense and antisense transcripts were analyzed in both strains and under all three conditions by RNASeq. A total of 10 757 antisense transcripts (ASTs) were assigned to sense signals from genes and untranslated regions, and 1 319 of these ASTs were expressed and were longer than 50 bases. Most of these (82%) were dual-use transcripts that contained antisense and sense regions, but ASTs (16%) were also observed that had no sense regions. Especially in metal-treated cells of strains CH34 and AE104, up- or down-regulated sense transcripts were accompanied by antisense transcription activities that were also regulated. The presence of selected asRNAs was verified by reverse transcription polymerase chain reaction (RT-PCR). Following metal stress, expression of genes encoding components of the respiratory chain, motility, transcription, translation, and protein export were down-regulated. This should also affect the integration of the metal efflux pumps into the membrane and the supply of the energy required to operate them. To solve this dilemma, transcripts for the metal efflux pumps may be stabilized by interactions with ASTs to allow their translation and import into the membrane. Alternatively, metal stress possibly causes recruitment of RNA polymerase from housekeeping genes for preferential expression of metal resistance determinants.

## Graphical Abstract

**Graphical Abstract:**
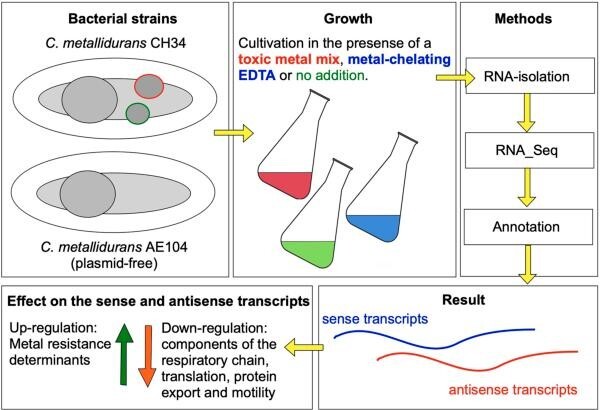
The transenvelope pump ZniCBA was upregulated under metal-starvation conditions and an antisense RNA was involved in regulation of this process. This suggests a possibly novel function of these efflux systems.

## Introduction

The transition metal contents of environments can vary over a wide range of concentrations and metal composition, not only for essential-but-toxic cations such as zinc, cobalt, and copper cations, but also for toxic-only ones such as the cadmium cation [1]. The beta-proteobacterium *Cupriavidus metallidurans* strain CH34 has an outstanding ability to thrive in environments rich in metals, for instance, auriferous soils or zinc-rich deserts, but it can also handle zinc starvation conditions [2–5]. Besides its bacterial chromosome, *C. metallidurans* contains a chromid and two large plasmids. Its metal resistance determinants are required for resistance to metals (Table 1): zinc (*znt*), cadmium (*cad)*, nickel and cobalt (*cnr*), cobalt (*dme*), copper (*cus, cop, sil, cop_2_, cup*), lead (*pbr*), silver (*sil*), mercury (*mer_1-4_*), chromate (*chr, chr_2_*), arsenic (*ars*), and finally to cobalt, zinc, cadmium (*czc)*. Many of these determinants were acquired by horizontal gene transfer and are located on the two plasmids, the chromid and genomic islands, which are part of the four replicons. The plasmid-free derivative AE104 of *C. metallidurans* contains only the chromosomal and chromid-encoded determinants (*znt, cad, mer_3_, ars, dme, cop_2_, cup, chr_2_*), leading to a metal resistance level comparable to that of *Escherichia coli* [1, 2, 6].

**Table 1.** Metal resistance determinants in *C. metallidurans**^a^*.

Determinant	Operon structure
Chromosomal determinants	
*hmz*	*hmzAB↔yodB-Rmet_3014; hmzSR←* (genomic island CMGI-4)
*cad*	*cdfX-Rmet_2300-Rmet_2301-cadR↔cadAC* (genomic island CMGI-1)
*cup*	*cupRA↔cupC*
*dme*	*dmeF-Rmet_0199←*
*ars*	*arsPHC_1_BC_2_IRM←* (genomic island CMGI-7)
Determinants on the chromid	
*znt-czc_2_*	*zntA↔czcI_2_C_2_B_2_‘//>czcR_2_S_2_-ubiG; czcA_2_B_2_‘‘←*
*zni/zne*	*zniAB↔zniC; zniS↔zniR; Rmet_5324↔zneRS; zneCAB↔zneR_2_S_2_*
*hmv*	→*hmvCBA‘/A‘‘*
*hmy*	→*hmyFCB; tnpA↔hmyA*
*nim*	*nimCAa-tnpB-nimA2b←*
*cus*	*→cusDCBAF*
*cop_2_*	*copD_2_C_2_B_2_A_2_↔copR_2_S_2_;* (*nim* determinant adjacent)
*chr_2_*	*chrF_2_A_2_B_2_-Rmet_3867←*
Determinants on plasmid pMOL30	
*czc*	*czcM↔czcICBADRSE; czcJ-ompP-tnpBA↔flgB-czcP*
*ncc*	*→nccCBa/BbA-nreB*
*sil*	*silABCD←*
*pbr*	*pbrD(BC)A↔pbrRTUa//→pbrUb*
*cop_1_*	*cobWE↔copHQ;copL↔copOFGJ; copID1C1B1A1↔copR1S1N;* *copKM↔copTV*
Determinants on plasmid pMOL28	
*cnr*	→*cnrYXHCBAT*
*chr_1_*	*chrZYPNOF_1_ECA_1_B_1_↔chrI-cnr* determinant

^a^The determinants are listed according to their host replicon. Bold-faced gene names indicate their location on the forward strand, semicolons directly adjacent genes, double arrows „↔‘ common promoter regions and indication of the direction of transcription, single arrows direction of transcription, double slashes „//‘ separation of gene regions, in case of the ancient, interrupted *czc2* gene region by 128 genes and in case of *pbr* by four genes. Adjacent determinants and genomic islands are also listed but the *mer* mercury resistance determinants were ignored. Gene names with dashes or small letters are interrupted genes.

Resistance to toxic concentrations of transition metal ions is based on transport and redox-reactions. Zn(II), Cd(II), Pb(II), Ni(II), and Co(II) are exported from the cytoplasm by members of the protein transporter protein families P-type ATPases (ZntA, CadA, CzcP, and PbrA; [7–9]), CDF (CzcD, DmeF, FieF; cation diffusion facilitator family; [10, 11]) or others (CnrT, AtmA) into the periplasm and from there by three-component trans-envelope efflux complexes composed of RND, MFP, and OMF (resistance-nodulation-cell division, membrane fusion, outer membrane factors; [12–15]) members to the outside, for instance, CzcCBA or CnrCBA. For copper resistance, inner membrane efflux by P-type ATPases (CupA and CopF) and trans-envelope efflux by CusCBA and SilCBA is supplemented by periplasmic oxidation of Cu(I) to the less toxic Cu(II) by components of the *cop* or *cop_2_* determinants [16, 17]. Furthermore, Hg(II) is reduced to the volatile Hg(0) oxidation state, arsenate is reduced to arsenite, which is exported, and chromate is also exported from the cell [18–21].

We aim to understand how all these transport systems, metal-binding proteins, one- and two-component regulatory systems and sigma factors interact to maintain transition metal homeostasis in *C. metallidurans*. To gain more insight into the regulation of expression of metal-resistance determinants, we here followed up on the preliminary evidence that antisense RNAs might be involved in *cus* and *cop_2_* regulation [22]. Therefore, we examined the possible involvement of antisense RNAs (asRNA) in regulation of metal homeostasis on a large scale using RNASeq-dependent transcriptome analysis with *C. metallidurans* CH34 wildtype and its plasmid-free variant AE104, under high, ambient, and low-metal conditions. We could demonstrate that antisense activities exist in *C. metallidurans*, which are co-up-regulated along with their cognate sense transcripts under metal stress in the case of most metal resistance determinants examined. Evidence for the existence of involvement of antisense activities in metal resistance of *C. metallidurans* is the first step to understand this process on the systems level. The second step, involving the influence of asRNAs on the copy number of the gene products of the associated sense mRNA, will be revealed in a subsequent publication [23]. In these two papers, we provide evidence for the existence and biological role of asRNAs in the regulation of metal-cation homeostasis in *C. metallidurans*.

## Results

### Challenging cells with metal cations

The transcriptomes of *C. metallidurans* CH34 and its plasmid-free derivative AE104 were analyzed by RNASeq in non-challenged cells, and under metal starvation and metal-stress conditions. It was anticipated that under these circumstances, the totality of the metal resistance determinants in *C. metallidurans* should be up-regulated. To exponentially growing cells, we added a 3.35 mM CH34-specific (*n* = 7) and a 1 mM AE104-specific MultiTox metal mix (*n* = 7) along with the separate addition of 50 µM ethylenediamintetraacetate (EDTA) to both strains (*n* = 4), or where no addition (*n* = 5) was made. The EDTA concentration used was not toxic to the cells but yielded a 90% Zur-dependent up-regulation of the gene encoding the zinc importer ZupT that was a clear indication for a zinc starvation condition [24–27]. The cells were used for RNASeq, as well as for determination of their metal content using ICP-MS (inductively-coupled plasma mass spectrometry, Table 2).

**Table 2.** Metal composition of the challenged cells as determined by ICP-MS*^a^*.

		Metals present in the strain-specific MultiTox metal mix							
Strain	condition Metal/cell	10^3^ Cr	10^3^ Co	10^3^ Ni	10^3^ Cu	10^3^ Zn	10^3^ As	10^3^ Cd	10^3^ Hg
CH34	No addition	4.8 ± 3.5	27 ± 10	20 ± 12	19 ± 10	83 ± 10	bQ	bQ	bQ
CH34	EDTA	bQ	8* ± *4	5 ± 3	7 ± 3	50 ± 12	bQ	bQ	bQ
CH34	MetalMix	**75.6 ± 29.2**	**727 ± 278**	**1343 ± 338**	**1587 ± 543**	**3558 ± 2283**	**3746 ± 1 380**	**85 ± 53**	**27 ± 6**
Chal. Conc., (µM)*^b^*		19	347	761	241	461	1503	15	0.37
AE104	no addition	bQ	27 ± 2	7 ± 3	10 ± 2	70 ± 16	bQ	bQ	bQ
AE104	EDTA	bQ	*18 ± 2*	7 ± 3	11 ± 5	59 ± 12	bQ	bQ	bQ
AE104	MetalMix	**37.9 ± 8.7**	**61 ± 16**	**59 ± 18**	**2035 ± 2251**	**92 ± 9**	**2034 ± 1 933**	**236 ± 233**	**21 ± 11**
Chal. Conc., (µM)*^b^*		7	7	9	120	5	849	3	0.18
		**Metals not present in the MultiTox metal mix, but present in the mineral salts medium used (TMM)**							
**Strain**	**condition metal**	**10^6^ Mg**	**10^3^ Ca**	**Mn**	**10^3^ Fe**	**Mo**			
CH34	No addition	10.7 ± 1.2	212 ± 92	531 ± 157	735 ± 80	2488 ± 359			
CH34	EDTA	9.6 ± 0.3	117 ± 23	680 ± 295	636 ± 60	2117 ± 100			
CH34	MetalMix	9.0 ± 0.5	213 ± 75	833 ± 311	609 ± 61	922 ± 41			
AE104	No addition	10.0 ± 0.9	164 ± 17	464 ± 175	740 ± 36	2670 ± 272			
AE104	EDTA	9.4 ± 0.4	154 ± 21	328 ± 134	675 ± 48	2378 ± 100			
AE104	MetalMix	10.0 ± 0.5	153 ± 41	491 ± 340	652 ± 55	2222 ± 48			

^a^ *Cupriavidus metallidurans* strains CH34 and AE104 were challenged with 50 µM EDTA, 3.35 mM of the CH34-specific, or 1 mM of the AE104-specific MultiTox metal mix. The metal content of the cells was determined by ICP-MS (*n* > 3). Bold faced letters indicate a significant (*D* > 1) up- and letters in italics a significant down-regulation compared to the unchallenged cells of the same strain; bQ, below limit of quantification (LOQ). *^b^*Concentration of an individual metal ion in the strain-specific metal mix. Please refer to the methods for details.

As expected, challenging the cells with MultiTox metal mixes increased the cellular content of the metals that were components of the mixture. Treatment with 50 µM EDTA decreased the cellular content of Co, Ni, Cu, and Zn in strain CH34. This demonstrated that the EDTA concentration used was sufficient to mediate metal starvation conditions. When confronted with the AE104-specific MultiTox metal mix, the cellular content of Cr, Co, Ni, and Zn was lower in AE104 compared to CH34 cells, which was probably caused by the lower metal ion concentrations in the AE104- compared to the CH34-specific MultiTox mix. The IC_50_ of the strain-specific metal mixture used for the respective strains indicated that CH34 could tolerate more cell-bound metal cations than its plasmid-free derivative AE104 (Table 2). The cellular content of the metals that were not part of the metal mix did not change, with the exception of a lower Mo content in metal-chased cells of strain CH34. Homeostasis of these metals was, therefore, not affected by the metal mix or by EDTA addition.

### Antisense activities exist in *C. metallidurans*

Using the RNASeq data, the program TraV Mac [28] was used to calculate the transcript abundances as NPKM values for all genes, 5′ and 3′ untranslated regions (5UTR, 3UTR), free and antisense transcripts (FT, AST) for strain CH34 and AE104, for all three conditions and for biological repeats. The transcripts were assigned to 6 841 annotated features for genes and RNAs 1 670 5UTRs, 1 691 3UTRs, and 10 757 ASTs (Supplementary Material M1, Supplementary material Table S1), and 385 FTs were found. The sense transcripts were named according to the number of the associated Rmet locus tag, the FTs and ASTs to the position of their first nucleotide, DNA strand and replicon (Supplementary Results). Only 1 391 ASTs and 12 FTs exhibited an abundance (NPKM, nucleotide activities per kilobase of exon model per million mapped reads) of NPKM > 10 and in the case of the ASTs a length of >50 bp (Tables S2 and S3).

It is important to note and detailed in the Supplement, an annotated AST may represent a discrete RNA molecule or a bin, a group of overlapping RNA molecules with some of them serving in total or in part as antisense RNA with respect to transcripts from the other DNA strand: an AST as annotated was a bin containing at least one transcript with an antisense region.

Most (82%) of the ASTs were “dual-use” transcripts or bins (Table S4), composed of at least one part or subgroup that was an antisense transcript for annotated features on the other DNA strand and in a least one other part or subgroup a sense transcript. Another 16% of the ASTs did not contain a portion that was a sense transcript. With the algorithm used, only few free transcript activities were found in non-challenged CH34 cells. In contrast, antisense transcription activities were found that covered the complete sense transcripts of genes with respect to their 5UTRs and 3UTRs. The majority of these antisense activities was of a small size, or were of low abundance. Nevertheless, antisense transcription activities clearly existed in *C. metallidurans* cells (Supplementary Results) in response to metal challenge.

The ASTs were associated with sense transcripts, genes, 5UTRs, and 3UTRs (Supplementary Material M2). There was no association of long ASTs to CMGIs (catabolic metabolic chromosomal islands, [29, 30], Fig. S1). No connection was found between the antisense abundance and usage of *rut* (Rho utilization) sites for loading of the “open-donut”-shaped ATP-dependent Rho factor for transcription-translation coupling [31–33] by termination (Supplementary Material M3, Supplementary Results, Figs. S2 and S3).

The 10 179 sense transcripts (genes, 3UTRs, 5UTRs) in untreated cells of *C. metallidurans* were sorted into groups of up- or down-regulated sense signals (>2, <0.5) with up- or down-regulated antisense signals (>2, <0.5; Table 3), for instance up–up, up–down, or up–not genes with the sense transcript being up-regulated and the antisense transcript up-, down- or not regulated, respectively. In the comparison of EDTA-treated CH34 cells with control cells even more than half of the signals did not change in their abundance of sense or antisense transcript. In CH34 cells treated with the MultiTox metal mix, 1 066 sense signals were up- and 1222 sense signals were down-regulated. About half as many sense signals were up- (565), or down-regulated (670) in the comparison of metal-mix-treated AE104 with CH34 cells. The number of changes in the other comparisons was lower, between 127 and 448 sense signals (Supplementary Results).

**Table 3.** Number of pairs of associated sense and antisense transcripts that were simultaneously up- or down-regulated^a^.

Regulation of		Comparison				
Sense	Antisense	CH34_M_0	CH34_E_0	0_A_C	M_A_C	E_A_C
Up	Up	274	10	3	79	4
Up	Not	575	135	86	391	100
Up	Down	93	14	9	54	10
Up	NAA	124	21	29	41	30
∑ Up		1066	180	127	565	144
Down	Down	214	7	28	109	20
Down	Not	717	198	288	441	200
Down	Up	115	15	13	47	10
Down	NAA	176	43	119	73	58
∑ Down		1222	263	448	670	288
Not	Up	893	566	434	768	486
Not	Down	879	762	1001	1004	980
Not	Not	5184	7225	5613	5174	6264
Not	NAA	935	1183	1203	454	533
	total	10179	10179	8826	8635	8695

^a^The number of associated pairs of sense (genes, 3UTRs and 5UTRs) and antisense transcripts that are simultaneously up-, down-, or not regulated is given. Up- and down-regulation above two-fold counted. The comparisons are M_0 (CH34 with MultiTox metal mix/no addition), E_0 (CH34 with EDTA/no addition), 0_A_C (no addition: AE104 value divided by CH34 value), E_A_C (same for EDTA values), or M_A_C (same for MultiTox metal mix values). NAA, no antisense transcript associated. The number of sense transcripts was lower in all comparisons with strain AE104 due to the lack of the plasmids and a different number of not annotated items in non-, metal-, and EDTA-challenged cells.

In all comparisons, more than half of the up- or down-regulated signals for sense transcripts were not accompanied by a change in abundance of the associated antisense signal (Table 3). In the comparison of metal-mix-treated CH34 with non-challenged cells (CH34_M_0), about 25% of the up-regulated sense transcripts were associated with an up-regulated antisense transcript, while about 9% were associated with a down-regulated antisense transcript. About 17% of the down-regulated sense transcripts in this comparison were associated with a down-regulated antisense transcript, and about 9% with an inversely regulated and up-regulated antisense transcript. These percentages were much lower in the other comparisons, with the exception of the comparison of metal mix-treated AE104 with CH34 cells (M_A_C, Table 3).

Treatment of *C. metallidurans* with a toxic metal mix had a strong impact on the transcriptome of this bacterium, changing 22% of the sense transcripts. In contrast, only 4% of the sense transcripts were changed in their regulation after EDTA treatment. In the comparison between AE104 and CH34 cells, 6.5% of the sense transcripts were different in the unchallenged cells, while 5% were differentially regulated in EDTA-challenged and, again, 14% in metal-challenged cells.

There was an enrichment of antisense activities in metal-challenged cells compared to other cells: Genes, 3UTRs, or 5UTRs differentially expressed following metal stress were often accompanied to a disproportionately high level by regulated antisense activities. This enrichment suggested a function for these antisense transcripts in the metal resistance response of *C. metallidurans*.

The complete results concerning the identification and association of the ASTs in *C. metallidurans* is provided in the “Supplementary Information” section.

### Transcriptional network in *C. metallidurans* CH34 cells after treatment with the MultiTox metal-mix

A total of 407 annotated genes (Table 4) among the 1 066 sense transcripts (Table 3) were up-regulated in the CH34_M_0 comparison (Supplementary Material M2). The remaining up-regulated transcript abundances were associated with 3UTRs and 5URTs. The 10 most strongly up-regulated (>300-fold, Table 4) genes after metal shock included 7 metal resistance genes, 1 transposase, 1 gene encoding a sensor histidine kinase, and 1 encoding an uncharacterized protein, both of unknown function (Table 4). All were accompanied by up-regulated antisense activities (Supplementary Material M2). The following 18 genes (>100-fold up-regulated) included 17 metal resistance genes plus 1 encoding a peptidase and 14 genes with up-regulated antisense activities. The majority of the most strongly up-regulated genes encoded metal resistance factors and were accompanied by up-regulated antisense activities. As the quotient of up-regulation decreased, genes encoding product with other functions became more prominent and the ratio of genes that were not accompanied by up-regulated antisense activities increased.

**Table 4.** Most strongly regulated genes in the comparisons*^a^*.

Locus tag	Gene	Q	Description
Up-regulated genes			
CH34_M_0 (407)			
Rmet_2315	*merA*	3959	Q8GQ26 Mercuric (Hg(II)) reductase
Rmet_2313	*merT*	3763	Q8GQ24 Mercuric transport protein MerT
Rmet_2317		2006	Q1LKY2 Transposase Tn3
Rmet_2314	*merP*	1335	Q8GQ25 Periplasmic mercuric ion binding protein MerP
Rmet_4026		566	Q1LG32 Putative periplasmic ligand-binding sensor protein
Rmet_0331	*arsC2*	467	Q1LRK9 Protein tyrosine phosphatase
Rmet_0329	*arsC1*	352	Q1LRL1 Arsenate reductase
Rmet_0330	*arsB*	345	Q1LRL0 Bile acid:sodium symporter
Rmet_0333	*arsR*	338	Q1LRK7 Transcriptional regulator, ArsR family
Rmet_5969		316	B2UEB6 Putative uncharacterized protein
CH34_E_0 (34)			
Rmet_0123		10.7	Q1LS67 TonB-dependent receptor
Rmet_5243		7.67	Q1LCM4 Methyltransferase type 11
Rmet_4296		6.00	Q1LFB4 Transcriptional regulator, LysR family
Rmet_3889		5.30	Q1LGG9 Transcriptional regulator, GntR family
Rmet_6329		5.25	Q1L9I8 HNH nuclease
Rmet_0615	*groS*	5.17	Q1LQS5 10 kDa chaperonin
Rmet_1146	*etfD*	4.85	Q1LP94 Electron transfer flavoprotein-ubiquinone oxidoreductase
Rmet_1967	*aatA*	4.56	Q1LLY0 Aminotransferase
Rmet_6103		4.50	Q58AD1 Acyl-CoA dehydrogenase
Rmet_5925		4.32	Q1LAP2 3-demethylubiquinone-9 3-methyltransferase
0_AE_CH (15)			
Rmet_4596	*czcC2*	5.40	Q1LEG8 Outer membrane efflux protein
Rmet_1717	*tauD*	3.00	Q1LMM8 Taurine catabolism dioxygenase TauD/TfdA
Rmet_5059	*atoD*	3.00	Q1LD55 3-oxoacid CoA-transferase, subunit A
Rmet_5701		3.00	Q1LBB6 Putative uncharacterized protein
Rmet_2312	*merR*	2.90	Q8GQ23 Organomercurial resistance regulatory protein MerR
Rmet_4560		2.83	Q1LEK4 Putative uncharacterized protein
Rmet_5087		2.50	Q1LD30 Uncharacterised conserved protein UCP012702
Rmet_5088	*dppA*	2.35	Q1LD29 Extracellular solute-binding protein, family 5
Rmet_4082		2.33	Q1LFX7 Putative uncharacterized protein
Rmet_1730		2.25	Q1LML7 Putative uncharacterized protein
M_AE_CH (305)			
Rmet_1125	*fdhD*	21.74	Q1LPB5 Formate dehydrogenase family accessory protein FdhD
Rmet_4750		16.65	Q1LE14 Rh-like protein/ammonium transporter
Rmet_1124		14.09	Q1LPB6 Oxidoreductase alpha (Molybdopterin) subunit
Rmet_0462		10.56	Q1LR78 Major facilitator superfamily MFS_1
Rmet_5782		10.30	Q1LB35 Putative uncharacterized protein
Rmet_2062	*glnA*	8.40	Q1LLN5 Glutamine synthetase
Rmet_5074		7.81	Q1LD40 NADPH-dependent FMN reductase
Rmet_2493		7.65	Q1LKF6 Putative uncharacterized protein
Rmet_4585	*ilvD*	7.56	Q1LEH9 Dihydroxyacid dehydratase
Rmet_5075	*msuE1*	7.29	Q1LD39 Putative uncharacterized protein
E_AE_CH (7)			
Rmet_1559	*trbI*	3.00	Q1LN34 Conjugation TrbI-like protein
Rmet_4362		3.00	Q1LF49 CoA-binding
Rmet_4918	*nosC*	3.00	Q1LDJ6 Cytochrome c, class I
Rmet_5134		3.00	Q1LCY3 AMP-dependent synthetase and ligase
Rmet_5139	*nrdD*	3.00	Q1LCX8 Ribonucleoside-triphosphate reductase, anaerobic
Rmet_2312	*merR*	2.88	Q8GQ23 Organomercurial resistance regulatory protein MerR
Rmet_4560		2.44	Q1LEK4 Putative uncharacterized protein
Downregulated genes (NPKM ≥10 in CH34_0)			
CH34_M_0 (654)			
Rmet_1628		0.02	Q1LMW5 Porin, Gram-negative type
Rmet_5058	bugT	0.02	Q1LD56 Uncharacterized protein UPF0065
Rmet_0133		0.03	Q1LS57 Nuclear export factor GLE1
Rmet_4565		0.03	Q1LEJ9 TonB-dependent receptor
Rmet_3139		0.03	Q1LIL5 Putative uncharacterized protein
Rmet_0523		0.03	Q1LR17 Auxin Efflux Carrier
Rmet_5793	cyoA	0.04	Q1LB24 Ubiquinol oxidase, subunit II

**Table 4.** Continued

Locus tag	Gene	Q	Description
Rmet_5616	bug	0.04	Q1LBK1 Uncharacterized protein UPF0065
Rmet_5156	paaE	0.04	Q1LCW1 Thiolase
Rmet_2976	furA	0.04	O30330 Ferric uptake regulation protein
CH34_E_0 (63)			
Rmet_3212		0.00	Q1LIE2 Putative uncharacterized protein
Rmet_2284	*yeiE*	0.07	Q1LL13 Transcriptional regulator, LysR family
Rmet_4505		0.12	Q1LEQ6 Transcriptional regulator, LysR family
Rmet_3222		0.12	Q1LID2 Putative uncharacterized protein
Rmet_0819		0.13	Q1LQ71 L-carnitine dehydratase/bile acid-inducible protein F
Rmet_0564		0.17	Q1LQX6 Putative uncharacterized protein
Rmet_4416		0.17	Q1LEZ5 Transcriptional regulator, GntR family
Rmet_0983		0.19	Q1LPQ7 Transcriptional regulator, LysR family
Rmet_0733		0.19	Q1LQF7 Histone deacetylase superfamily
Rmet_2234		0.20	Q1LL63 ABC transporter-related protein
0_AE_CH (5)			
Rmet_1251	*tnp*	0.09	Q1LNY9 Putative uncharacterized protein
Rmet_0123		0.20	Q1LS67 TonB-dependent receptor
Rmet_5171	*cspA*	0.29	Q1LCU6 Cold-shock DNA-binding protein family
Rmet_4640		0.31	Q1LEC4 Putative uncharacterized protein
Rmet_2987		0.45	Q1LJ16 Putative uncharacterized protein
M_AE_CH (159)			
Rmet_1928		0.02	Q1LM19 Putative uncharacterized protein
Rmet_1926	*yfaV*	0.02	Q1LM21 Major facilitator superfamily MFS_1
Rmet_1929		0.04	Q1LM18 Transcriptional regulator, GntR family
Rmet_3620	*degP*	0.05	Q1LH84 Peptidase S1C, Do
Rmet_2303	*cadA*	0.08	A7HYL0 Heavy metal translocating P-type ATPase
Rmet_2304	*pbrC2*	0.08	Q8GQ15 Lipoprotein signal peptidase
Rmet_1251	*tnp*	0.11	Q1LNY9 Putative uncharacterized protein
Rmet_4191		0.12	Q1LFL8 Transcriptional regulator, LysR family
Rmet_2305		0.13	Q8GQ16 Putative uncharacterized protein ORF C95
Rmet_2178	*ppk*	0.15	Q1LLB9 Polyphosphate kinase
E_AE_CH (4)			
Rmet_1251	*tnp*	0.09	Q1LNY9 Putative uncharacterized protein
Rmet_5171	*cspA*	0.26	Q1LCU6 Cold-shock DNA-binding protein family
Rmet_4640		0.42	Q1LEC4 Putative uncharacterized protein
Rmet_0123		0.48	Q1LS67 TonB-dependent receptor

^a^This table gives in the comparisons all annotated genes (omitting 3UTRs, 5UTRs) in the decreasing (up-regulation) or increasing order (down-regulation) of the ratio Q, if the distance value was *D* >1. The comparisons were again metal-challenged CH34 cells divided by non-challenged cells, EDTA-treated CH34 cell divided by non-challenged cells, non-challenged AE104 divided by non-challenged CH34 cells, the same with metal-challenged or EDTA-treated AE104 divided by CH34 cells. For down-regulated genes, only those that were expressed in non-challenged CH34 cells (NPKM >10) were listed. The numbers following the comparison headlines indicate the total number of annotated genes that were *Q* >2 or *Q* <0.5, with *D* >1. The Rmet locus tags also indicate the associated replicons of these genes, Rmet_0001 to Rmet_3615 chromosome, to Rmet_5941 chromid, to Rmet_6181 pMOL30, to Rmet_6346 pMOL28 and from Rmet_6347 genes from all four replicons that were annotated in a later annotation round.

All active metal resistance determinants in CH34 were up-regulated following treatment with the MultiTox metal mix (Supplementary  Material M2), which confirms the findings of previous gene array data [34]. Most determinants that encoded RND-driven trans-envelope efflux systems, or components thereof (*czc, czc_2_, cnr, zni/zne, nimC, cus, sil*), were accompanied by antisense transcription activities (Table 5). Especially the sense transcripts of the major metal resistance determinants (*czc, cnr, cop, cup, chr* and *ars)* were accompanied by up-regulated antisense activities while recessive determinants (*nre/ncc, hmv, hmz*) were not. This strengthened our premise of the importance of the antisense activities for full regulatory function of the active and dominant metal resistance determinants. In comparison, the *cus* and *cop_2_* determinants that were not plasmid-encoded were accompanied by down-regulated or unregulated antisense transcripts (Table 6, Figs. S4 and S5). Plasmid-encoded metal resistance genes appear consistently to have an associated up-regulated antisense transcript in addition to the up-regulated sense transcript.

**Table 5.** Regions containing up-regulated sense and up-regulated antisense transcripts after incubation of the CH34 cells with MultiTox metal mix*^a^*.

Region	Sense signals	Up-Up	Up-down	Up-not	Up-up-regulated annotated genes
*czc*	24	16	0	0	*czcP, flgB, ompP, czcJ, czcE, czcS, czcR, czcD, czcA, czcB, czcC, czcI, czcN, czcM*
*czc_2_*	15	6	0	0	*zntA, czcI_2_, czcR_2_*
*cnr*	8	5	0	2	*cnrY, cnrX, cnrH, cnrB, cnrA*
*zni/zne*	27	4	1	1	*zniA, zniB, zneP*
*nre/ncc*	6	0	0	1	
*hmv*	3	0	0	0	
*hmy*	5	0	0	1	
*nim*	8	3	2	0	*nimC, nimA2, nimB*
*hmz*	8	1	0	0	*Rmet_3014*
*cus*	6	0	3	1	
*sil*	5	1	0	1	*silC*
*cusF_2_*	3	2	0	0	*cusF_2_*
*cad*	12	5	0	2	*cadC* and downstream genes
*pbr*	11	4	0	0	*pbrR, pbrA, pbrB/C, pbrD*
*cop*	35	19	0	2	*copT, copM, copK, copN, copS_1_, copR_1_, copA_1_, copB_1_, copC_1_, copD_1_, copI, copJ, copG, copF, copL, copQ, copH*
*cup*	7	4	0	3	*betA2, pldB, cupR, cupA*
*cop_2_*	12	2	1	3	*copA_2_*
*chr*	14	9	0	3	*chrP, chrN, chrO, chrF_1_, chrE, chrC, chrA1, chrB1, chrI*
*chr_2_*	7	0	0	4	
*ars*	16	9	0	0	*arsP, arsH, arsC_1_, arsB, arsC_2_, arsI, arsR, arsM*
*mer^b^*	28	4	0	6	*merP, merA'', merR, Rmet_2317*
*gsh*	8	0	2	1	
*isc*	9	2	4	0	*iscU, iscA*
*agr*	9	1	0	1	
*pp/pst*	18	10	0	0	*ppk, phoR, phoB, phoU, pstB, pstA, pstC, pstS, rrmJ*
*pho*	6	0	0	3	
*gig*	8	3	1	4	*gigT, rpoQ*
*ompP*	3	0	0	3	
*phaC*	5	2	0	2	*phaC_3_, phaB_3_*
*chrF_3_*	9	2	0	3	*Rmet_5224, Rmet_5225*
*zwf*	12	3	1	5	*Zwf, Rmet_5798*
*gtr_1_*	12	9	0	0	*gtrM_3_, tnpA, tnpA, gtrM_1_, gtrA_1_, gtrB_1_*
*gtr_2_*	11	0	0	4	
*mmr*	5	3	0	1	*flgB, mmrQ*
					
Others					Examples
Up-up	145	145	0	0	*grxC, acrA, fieF, copO*
Up-down	78	0	78	0	*atmA, rpoN*
Up-not	518	0	0	518	*dnaK, clpS, rpoH, folB, bfr,*
Up-NAA	124				*ptx*

^a^In the comparison M_0 for *C. metallidurans* CH34, all regions containing up-regulated sense signals (*Q* >2, NPKM, 3UTR, and 5UTR signals) and up-regulated antisense transcripts (up–up) are listed with the number of signals, those up–up, up-down (antisense down), up-not regulated (antisense not regulated) and the names of the genes that were up-up-regulated. *^b^*summary of several *mer* loci and chromosomal and plasmid DNA. Full data: Supplementary Material M2. NAA, no antisense associated.

**Table 6.** Influence of antisense activities on the expression and regulation of copper resistance genes in *C. metallidurans^a^.*

					Comparisons										
					CH34_M_0		CH34_E_0		0_AE104_CH34		M_AE104_CH34		E_AE104_CH34		
locus tag	gene	MeanS	MeanAST	S/AS	Q_sense	Q_ast	Q_sense	Q_ast	Q_sense	Q_ast	Q_sense	Q_ast	Q_sense	Q_ast	Description
*cup, copF*															
Rmet_3523	*cupR*	62.3	*8.00*	*7.79*	**35.4**	**59.2**	1.07	1.17	0.97	1.17	***0.39***	***0.02***	0.72	***0.25***	Q1LHI1 Transcriptional regulator, MerR family
Rmet_3524	*cupA*	15.0	47.3	0.32	**126**	**10.0**	1.07	0.82	1.00	0.77	***0.42***	**2.42**	0.94	0.82	Q1LHI0 Heavy metal translocating P-type ATPase
Rmet_3525	*cupC*	**184**	**334**	0.55	**42.9**	0.77	5.00*^b^*		0.79	0.92	0.86	0.60	0.79	1.06	Q1LHH9 Heavy metal transport/detoxification protein
Rmet_6119	*copF*	41.3	10.0	4.13	**13.5**	**8.97**	1.00	**2.00**							Q58AE3 Heavy metal translocating P-type ATPase
*cop2, cop1*															
Rmet_5668	*copD_2_*	*4.3*	12.3	0.35	**25.5**	***0.16***	1.40	1.16	1.00	1.46	**2.62**	**10.3**	1.07	1.19	Q1LBE9 Copper resistance D
Rmet_5669	*copC_2_*	*1.7*	4.67	0.36	**37.0**	***0.50***			1.40	***0.21***	**2.84**	0.57	1.00	***0.12***	Q1LBE8 Copper resistance protein CopC
Rmet_5670	*copB_2_*	*3.7*	0.67	5.50	**15.6**	*0.50*	0.64	1.50	0.64	0.50	**2.64**	**102**	1.14	1.00	Q1LBE7 Copper resistance B
Rmet_5671	*copA_2_*	*3.3*	8.67	0.38	**19.9**	**3.35**	0.80	0.96	0.70	0.85	**3.22**	1.17	1.00	1.04	Q1LBE6 Copper-resistance protein CopA
Rmet_5672	*copR_2_*	*9.7*			**5.52**		0.84	0.64	1.00		1.82	1.74	1.03	0.81	Q1LBE5 Two component response regulator
Rmet_5673	*copS_2_*	*5.3*			**7.38**		1.19		1.13		1.19	**2.32**	0.89	***0.02***	Q1LBE4 Sensor protein
Rmet_6110	*copS*	27.7	*0.67*	**41.5**	**21.2**	**277**	0.99	**11.0**							Q58AD4 Sensor protein
Rmet_6111	*copR*	24.0	*8.00*	3.00	**25.9**	**23.1**	0.96								Q58AD5 Two component response regulator
Rmet_6112	*copA*	*7.0*	20.7	0.34	**48.1**	**11.0**	1.18	1.18							Q58AD6 Copper resistance protein CopA
Rmet_6113	*copB*	*5.7*	*1.33*	4.25	**28.5**	**171**	0.88	2.50							Q58AD7 CopB protein (Copper resistance B)
Rmet_6114	*copC*	20.3	31.0	0.66	**20.4**	**11.1**	1.20	***0.09***							Q1LA53 Copper resistance protein CopC
Rmet_6115	*copD*	29.3	31.00	0.95	**16.8**	**11.1**	1.08	***0.09***							Q58AD9 Copper resistance protein CopD
*cus, sil*															
Rmet_5030	*cusD*	**291**			1.70		1.01		0.95		1.82	**11.3**	0.99	0.86	Q1LD84 Putative uncharacterized protein
Rmet_5031	*cusC*	*1.7*	*2.00*	0.83	**14.0**	***0.17***	1.00	1.00	1.00	0.67	**6.33**	**68**	1.00	0.67	Q1LD83 Putative outer membrane cation efflux protein
Rmet_5032	*cusB*	*2.0*	35.0	0.06	**12.2**	***0.13***	0.83	***0.14***	1.00	0.57	**6.92**	**4.86**	1.40	0.93	Q1LD82 Secretion protein HlyD
Rmet_5033	*cusA*	*1.7*	35.0	0.05	**14.2**	***0.13***	0.80	***0.14***	1.00	0.57	**5.99**	**4.86**	1.50	0.93	Q1LD81 Heavy metal efflux pump CzcA
Rmet_5034	*cusF*	*2.3*	35.0	0.07	**21.3**	1.90	0.67	1.11	0.86	0.57	**5.32**	***0.34***	1.75	1.03	Q1LD80 Periplasmic protein
Rmet_6133	*silD*	**129**	*8.67*	**14.85**	1.84	**2.38**	1.06	***0.08***							Q1LA34 Putative uncharacterized protein
Rmet_6134	*silC*	*4.0*	*1.33*	3.00	**3.00**	**15.5**	1.08	0.75							Q58AF2 Outer membrane silver efflux protein
Rmet_6135	*silB*	*6.3*	*8.33*	0.76	**2.26**	1.04	0.89	1.12							Q58AF3 Silver efflux protein
Rmet_6136	*silA*	36.7	*3.00*	12.2	1.71	0.89	0.98	0.89							Q58AF4 Silver efflux protein
*gig, rpoQ*															
Rmet_4682	*gigT*	*5.7*	*5.67*	1.00	**3.12**	**2.82**	1.06	1.35	0.82	**2.65**	0.98	1.17	0.78	0.61	Q1LE82 DoxX
Rmet_4683	*gigB*	*3.3*	*5.67*	0.59	**3.30**	***0.47***	1.10	***0.41***	1.00	**2.65**	1.09	**7.00**	0.82	**2.00**	Q1LE81 Putative uncharacterized protein
Rmet_4684	*gigA*	*8.7*	28.3	0.31	**2.42**	1.72	0.81	0.98	0.92	0.53	0.97	***0.38***	1.05	0.71	Q1LE80 Putative uncharacterized protein
Rmet_4685	*gigP*	*7.7*	28.3	0.27	**2.26**	1.72	1.00	0.98	0.70	0.53	1.13	***0.38***	1.06	0.71	Q1LE79 Putative uncharacterized protein
Rmet_4686	*rpoQ*	69.3	4.33	**16.0**	**2.19**	**3.23**	1.15	1.31	0.95	1.31	0.64	1.93	0.66	1.06	Q1LE78 Sigma-24 (FecI-like)
Rmet_4687	*rsqA*	27.0			**2.11**		1.01		0.91		0.79	0.70	0.80	1.00	Q1LE77 Putative uncharacterized protein
*gshA, gshB*															
Rmet_0242	*gshA*	**329**	69.3	4.75	**2.67**	***0.17***	1.07	***0.13***	1.05	***0.11***	0.62	0.75	0.87	0.86	Q1LRU8 Glutamate–cysteine ligase GshA
Rmet_0243	*gshB*	**268**	69.3	3.87	**2.36**	***0.17***	1.07	***0.13***	1.12	***0.11***	0.70	0.75	0.82	0.86	Q1LRU7 Glutathione synthase

The locus tag, gene name, NPKM values of the sense and antisense transcripts is shown followed by the ration S/AS of both values. Bold-faced are here transcripts with NPKM > 100 or S/AS ratios > 10, which indicates a low probability of an antisense influence. The comparisons are metal-challenged to non-challenged CH34 cells, EDTA-treated to non-treated CH34 cells, unchallenged, metal-, and EDTA- challenged AE104 to CH34 cells. The ratios Q of the sense and antisense NPKM values is shown. Bold if Q >2, italics and bold if Q <0.5. Deviations are in the Supplementary Material. Empty cells indicate missing sense or antisense transcripts, for instance for plasmid-encoded factors in the plasmid-free strain AE104.

*^b^* Not significant. Plasmid genes on light-gray fields.

All plasmid-encoded *czc* genes were up-regulated and accompanied by up-regulated antisense activities (Supplementary  Material M2, Supplementary Results). A similar interaction network of up-regulated sense transcripts and antisense activities after metal shock was also observed for other metal resistance determinants, for instance, the interrupted “ancient” *czc_2_* region adjacent to *zntA* on the chromid. For the cobalt-nickel resistance determinant *cnr* on plasmid pMOL28, the antisense activities may also interact with adjacent genes outside of the metal-resistance determinants and in the case of *cnr* this was with the adjacent chromate-resistance determinant. Other metal resistance determinants, such as *chr, chr_2_*, and *ars*, were also up-regulated after metal stress and were accompanied in the case of most genes by up-regulated antisense activities (Supplementary  Results).

These data demonstrate that following metal stress, metal resistance determinants are up-regulated, as anticipated, and that in most but not all cases this up-regulation is paralleled by an up-regulation of an associated antisense activity (Table 5). A similar pattern was exhibited by genes encoding phosphate uptake by the PtsABC transporter, iron-cluster and glutathione biosynthesis, and the *gig* genes (for “gold-induced”). In these cases, the respective gene products were needed to protect the cells against damage caused by the high concentration of toxic metals. An antisense activity that reduces expression of genes encoding such important proteins would be counterproductive. This suggests that for metal resistance genes, antisense transcripts may interact with sense transcripts to stabilize them. Alternatively, the asRNAs might be involved in synchronize gene expression to yield the optimal ratio of the subunits of protein complexes or those of different proteins of a resistance system.

### Genes down-regulated following metal stress

Among the down-regulated 654 genes (<0.08-fold; only annotated genes expressed in non-challenged CH34 cells were counted, Table 4), the most strongly down-regulated ones included two TonB-dependent outer membrane receptors and a general porin. No gene was accompanied by any change in the potential antisense activities.

**Figure 1.**
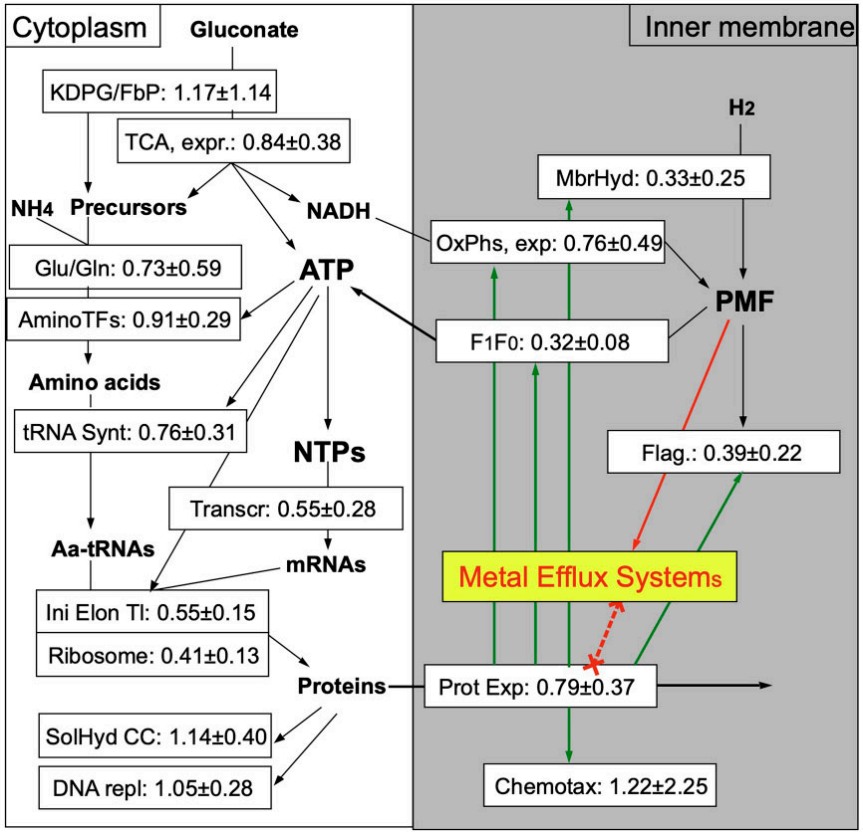
Change of the flow of energy and metabolites in metal-shocked *C. metallidurans* CH34 cells. The carbon source gluconate is degraded by the 2-keto-3-deoxy-6-phospo-gluconate (KDPG) pathway and the lower branch of the fructose-1,6-bisphosphate pathway (FbP) via pyruvate to acetyl-CoA, which enters the TCA for degradation to CO_2_ and redox equivalents, and which donates electrons to the respiratory chain that conserves energy as proton motive force (OxPhs, PMF). Fbp and TCA also produce precursors for the synthesis of amino acids with NH_4_ being assimilated via glutamate, glutaminate, and amino-transferases. These are needed to produce loaded tRNAs for translation mediated by ribosomes, initiation, and elongation factors. The F_1_F_0_ ATPase uses the PMF to synthesize ATP. Depicted are the membrane-bound hydrogenase, flagella, protein export systems and chemotaxis in the membrane (gray) and transcription, soluble hydrogenase and DNA replication in the cytoplasm (white). The numbers in the boxes indicate the mean quotient of down-regulation of the genes for the enzymes of the respective pathway. The green arrows indicate that protein export is necessary to bring the respective proteins into the membrane. The synthesized metal efflux system competes with other proteins for transport into the membrane (red, bold, dashed line) and for the PMF as driving force (red arrow). All crude data are shown in  Supplementary Material M2.

The next gene-set with a lower down-regulation quotient included genes involved in hydrogenase synthesis, in motility, respiratory chain and translation complexes (Supplementary Results, Supplementary Material M2, Fig. 1). Not only were the genes for the membrane-bound hydrogenase down-regulated after metal shock, but also the *atp* genes encoding the F_1_F_0_-ATPase, most *nuo* genes encoding the NADH-quinone oxidoreductase, *cox* genes for cytochrome *c* oxidase, *cyo* genes encoding a cytochrome *o* ubiquionol oxidase (initially annotated as cytochrome *c* oxidase) and *app, cyd* and *cyo* genes encoding other respiratory chain components. This indicated that metal shock resulted in a down-regulation in the synthesis of most membrane-bound proteins involved in energy conservation from the generation of the proton motive force to the synthesis of ATP (Fig. 1, OxPhs).

While the chemotaxis genes were only mildly down-regulated following metal shock, the *flh/flg* and the *fla/fli* genes encoding components required for synthesis of flagella were all down-regulated (Supplementary Results, Fig. 1, Flag). Most genes of the tricarboxylic acid cycle (TCA), which provided NADH for the respiratory chain as well as anabolic metabolites (Fig. 1, TCA), were unchanged in expression with a mild down-regulation in expression of the genes for the membrane-bound succinate dehydrogenase. After metal shock, expression of the genes encoding enzymes for the degradation of gluconate to the level of acetyl-CoA and those for the synthesis of amino acids, including genes required for their associated tRNAs, were changed little, except that the initial input reactions (*gntT, gntK, edd)* for carbon from gluconate and nitrogen via the ATP-dependent GlnA-GltDB enzymes were down-regulated. This may decrease the overall flow of metabolites from gluconate to amino acids loaded to their tRNAs (Fig. 1, Supplementary Results).

The genes for the ribosomal proteins were all down-regulated ∼0.4-fold (0.13- to 0.79-fold, down-regulation of an antisense activity covering the genes encoding 25 ribosomal proteins, Fig. 1). Expression of the genes encoding the translational initiation factors was mildly down-regulated, while that of the genes encoding the elongation factors was more strongly down-regulated. Regarding the genes whose products are involved in protein export, expression of *ffh* and *ftsY* encoding membrane integration via the signal recognition particle was not down-regulated but expression levels of all genes for the general secretion pore were, including the leader peptidases and YidC, with the exception of *secA*. This indicates that the energy-consuming translation and protein export by the general secretion pathway were down-regulated following metal shock (Fig. 1).

Compared to the genes for the ribosomal proteins, no gene for subunits of the DNA polymerase III or the other DNA polymerases were down-regulated in their expression. DNA replication and repair processes were thus unimpaired. In contrast (Table 7), the genes encoding the RNA polymerase core enzyme, the *nus* and *gre* factors, *rho* and the major house-keeping sigma factor *rpoD_1_* were all down-regulated between 0.34- and 0.79-fold (mean value 0.55-fold), with the exception of *rpoZ* (mildly up-regulated 1.4-fold). For *rpoB, rpoC, nusG, greA*, and *rpoD_1_*, this was accompanied by an up-regulation of the associated antisense activity and for *rpoA* and *nusB* with a down-regulation. Among the genes encoding the alternative sigma factors, expression of *rpoH, rpoN, rpoQ*, and *cnrH* was up-regulated, with *cnrH* showing a 360-fold up-regulation of its antisense activity; expression of *rpoS* was unaffected. Only the gene encoding chemotaxis and motility sigma factor FliA was down-regulated (Table 7). Not only the translation but also the general transcription activity of metal-shocked *C. metallidurans* cells was down-regulated, possibly to conserve energy by decreasing the energy-intensive process of protein biosynthesis.

**Table 7.** Influence of antisense activities on the expression and regulation of the genes needed for transcription in *C. metallidurans^a^.*

					Comparisons										
					CH34_M_0		CH34_E_0		0_AE104_CH34		M_AE104_CH34		E_AE104_CH34		
locus tag	gene	MeanS	MeanAST	S/AS	Q_sense	Q_ast	Q_sense	Q_ast	Q_sense	Q_ast	Q_sense	Q_ast	Q_sense	Q_ast	Description
Rmet_3291	*rpoA*	**783**	58.0	**13.5**	***0.43***	***0.41***	1.14	*0.95*	1.33	0.82	1.84	1.94	1.09	0.85	Q1LI63 DNA-directed RNA polymerase subunit alpha
Rmet_3334	*rpoB*	**401**	39.7	**10.1**	0.63	**1.84**	1.13	*0.97*	1.34	0.78	1.30	0.56	1.11	0.94	Q1LI20 DNA-directed RNA polymerase subunit beta
Rmet_3333	*rpoC*	**379**	39.7	9.56	0.79	**1.84**	*1.09*	*0.97*	1.28	0.78	1.07	0.56	1.10	0.94	Q1LI21 DNA-directed RNA polymerase subunit beta'
Rmet_0857	*rpoZ*	**2033**	**126**	**16.2**	1.38	1.21	*0.99*	*1.07*	0.90	0.90	0.72	0.62	0.95	0.87	Subunit Omega
Rmet_2032	*nusA*	**562**	8.00	**70.3**	0.50	*0.88*	*0.96*	*0.83*	1.15	0.75	1.91	0.90	1.14	0.70	Q1LLR5 NusA antitermination factor
Rmet_2695	*nusB*	**144**	**475**	0.30	0.51	***0.45***	*1.09*	*1.01*	1.30	0.81	1.65	1.71	1.05	0.91	Q1LJV8 N utilization substance protein B homolog
Rmet_3339	*nusG*	**1280**	39.7	**32.3**	***0.45***	**1.84**	*1.04*	*0.97*	1.22	0.78	**2.32**	0.56	1.13	0.94	Q1LI15 Transcription antitermination protein nusG
Rmet_2192	*greA*	**485**	36.3	**13.4**	***0.34***	**5.09**	*1.00*	**1.52**	1.14	0.76	**2.59**	0.72	1.12	0.64	Q1LLA5 Transcription elongation factor
Rmet_0859	*greB*	**225**	**231**	0.97	0.54	*1.04*	*1.02*	*0.98*	1.00	1.03	1.41	0.97	0.96	1.10	Q1LQ31 Transcription elongation factor
Rmet_2135	*rho*	**509**	**230**	2.21	0.63	*0.87*	*1.06*	*1.18*	1.08	0.99	1.67	1.25	1.03	0.96	Q1LLG2 Transcription termination factor Rho
Rmet_2606	*rpoD1*	**426**	19.3	**22.1**	0.65	**5.00**	*1.03*	*0.90*	1.18	0.83	1.79	***0.24***	1.16	0.81	Q1LK43 RNA polymerase sigma factor
Rmet_4661	*rpoD2*	**12.0**	6.00	2.00	*1.14*	**1.89**	0.86	0.83	0.78	1.33	1.02	1.74	1.10	1.07	Q1LEA3 RNA polymerase sigma factor
Rmet_2115	*rpoS*	**392**	**136**	2.89	1.28	**1.55**	*1.05*	*1.18*	1.11	***0.06***	0.89	0.83	1.03	0.90	Q1LLI2 RNA polymerase sigma factor
Rmet_0272	*rpoH*	**381**	**345**	1.11	**2.48**	0.54	*1.05*	*1.03*	0.96	0.98	0.83	1.54	0.85	0.87	Q1LRR8 RNA polymerase sigma factor
Rmet_3702	*fliA*	**43.0**			***0.47***		*1.05*		1.02		0.90	0.76	0.81	1.21	Q1LH02 RNA polymerase sigma factor
Rmet_0303	*rpoN*	**352**	81.3	4.33	**2.16**	***0.13***	*1.05*	*1.16*	0.91	1.17	0.73	**7.00**	0.90	***0.06***	Q1LRN7 Sigma-54 (RpoN)
Rmet_2425	*rpoE*	**806**	10.3	**78.0**	1.54	1.16	0.95	*0.97*	0.95	0.71	0.88	0.78	1.10	0.77	Q1LKM4 RNA polymerase sigma factor
Rmet_1120	*rpoI*	**135**	2.00	**67.7**	0.81	**1.67**	*1.10*	0.50	0.83	0.50	1.19	0.60	0.91	**2.33**	Q1LPC0 Sigma-24 (FecI-like)
Rmet_4499	*rpoJ*	**10.7**	1.00	**10.7**	*1.25*	**3.67**	1.31	*1.33*	1.06	1.33	1.28	***0.45***	1.07	1.25	Q1LER2 Sigma-24 (FecI-like)
Rmet_4001	*rpoK*	**53.0**	2.67	**19.9**	0.66	*0.88*	*1.07*	*1.00*	0.75	0.88	1.31	1.00	1.06	0.88	Q1LG57 Sigma-24 (FecI-like)
Rmet_3280	*rpoL*	**240**	4.00	**60.1**	*1.24*	*0.92*	*1.16*	0.83	1.23	0.58	0.86	**1.00**	0.90	1.00	Q1LI74 Sigma-24 (FecI-like)
Rmet_5400	*rpoM*	**9.3**	2.33	4.00	*1.07*	*0.29*	*0.93*	*0.71*	0.93	***0.14***	1.00	**3.00**	1.15	0.60	Q1LC67 Sigma-24 (FecI-like)
Rmet_0597	*rpoO*	**28.3**	1.33	**21.3**	*0.69*	*0.75*	*0.92*	*0.75*	0.82	***0.25***	1.07	*0.00*	0.81	***0.33***	Q1LQU3 Sigma-24 (FecI-like)
Rmet_1648	*rpoP*	**17.0**	1.67	**10.2**	*0.94*	*1.00*	0.54		0.90	***0.40***	1.15	1.00	1.02	**2.25**	Q1LMU5 Sigma-24 (FecI-like)
Rmet_4686	*rpoQ*	**69.3**	4.33	**16.0**	**2.19**	**3.23**	*1.15*	1.31	0.95	1.31	0.64	1.93	0.66	1.06	Q1LE78 Sigma-24 (FecI-like)
Rmet_0910	*rpoR*	**378**	664	0.57	0.64	0.53	*0.91*	*0.97*	0.92	0.98	1.26	1.03	0.99	0.99	Q1LPY0 Sigma-24 (FecI-like)
Rmet_6207	*cnrH*	**61.0**	3.00	**20.3**	**7.64**	**361**	*1.13*	**5.44**							P37978 RNA polymerase sigma factor cnrH

The locus tag, gene name, NPKM values of the sense and antisense transcripts is shown followed by the ration S/AS of both values. Bold-faced are here transcripts with NPKM >100 or S/AS ratios >10, which indicates a low probability of an antisense influence. The comparisons are metal-challenged to non-challenged CH34 cells, EDTA-treated to non-treated CH34 cells, unchallenged, metal- and EDTA- challenged AE104 to CH34 cells. The ratios Q of the sense and antisense NPKM values is shown. Bold if Q >2, italics and bold if Q <0.5. Deviations are in the Supplementary Material. Empty cells indicate missing sense or antisense transcripts, for instance for plasmid-encoded factors in the plasmid-free strain AE104.

In total, treatment with multiple metal cations of *C. metallidurans* CH34 led to an up-regulation of metal resistance determinants and in most cases these were accompanied by an associated up-regulation of antisense activities. These determinants encode a multitude of membrane-bound metal efflux systems. Simultaneously, the genes for the F_1_F_0_ ATPase and respiratory chain components are down-regulated, which counterintuitively should reduce the amount of available energy to the cell. Consequently, high energy-consuming processes such as motility, transcription, translation, and protein export are down-regulated, perhaps to retain sufficient energy to ensure synthesis of the efflux systems. These processes also lead to a higher competition between the efflux systems, F_1_F_0_-ATPase and the respiratory chain components for protein export capacity by the general secretion system (Fig. 1, Supplementary  Results for more details).

### Other comparisons

The up-regulated genes (>5-fold) identified in *C. metallidurans* after treatment with EDTA (CH34_E_0 comparison) were difficult to interpret in terms of roles in metal-limitation (most strongly regulated genes shown in Table 4), except for possibly the five-fold up-regulation of *groS* encoding a chaperonin, indicating possible problems with protein folding under this stress. Similarly, the top 10 of the 63 down-regulated genes (≤0.23-fold) also did not yield many insights (Table 4). Among the genes for transport proteins (Table 8), only *zupT* was up-regulated (1.63-fold). None of the genes for the siderophore biosynthesis cluster downstream of the sigma factor genes *rpoI* was up- or down-regulated so that the EDTA-treated cells appeared to retain sufficient iron supply by the siderophore produced in the EDTA-trated cells at the same level as in the control cells (Supplementary Material M2, “Transport”). This was supported by the unchanged amount of iron in these cells (Table 2). Except for *zntA, cadA*, and *czcD*, none of the genes for efflux systems, which were up-regulated following metal shock, was down-regulated in EDTA-treated cells. The “anabolic” Cu(I)-exporting P-type ATPase genes *rdxI* and *ctpF* and the Co(II) exporter gene *dmeF* were not up-regulated following metal stress, but the *ctpF* antisense activity was strongly up-regulated (15-fold) under metal stress. There was little change in the expression of the genes encoding components of the metal transportome of the inner membrane under conditions of metal starvation, except for the down-regulation of *zntA, cadA*, and *czcD* genes (Table 8). With the exception of the TonB-dependent Rmet_0123, the already present metal transportome plus some Zur-dependent up-regulation of *zupT* and the *cobW1* cluster was sufficient for *C. metallidurans* to survive EDTA-mediated metal starvation. In contrast to metal resistance determinants that counteracted metal shock, no “starvation-resistance” determinants seemed to exist for non-iron transition metals in *C. metallidurans*, except the Zur regulon. Moreover, no expression change for the sigma factors was observed (Table 7) with the exception of up-regulation of the antisense activities for the plasmid-encoded *cnrH*.

**Table 8.** Influence of antisense activities on the expression and regulation of the genes for main metal ion uptake and efflux systems in *C. metallidurans^a^.*

					Comparisons									
					CH34_M_0		CH34_E_0		0_AE104_CH34		M_AE104_CH34		E_AE104_CH34	
Locus tag	Gene	MeanS	MeanAST	S/AS	Q_sense	Q_ast	Q_sense	Q_ast	Q_sense	Q_ast14	Q_sense	Q_ast	Q_sense	QS_AS
Uptake systems systems														
Rmet_3052	*corA_1_*	55.0	**125**	0.44	0.55	***0.02***	*1.05*	*1.04*	1.04	0.83	1.77	**50.0**	1.03	0.92
Rmet_0036	*corA_2_*	66.3	**373**	0.18	0.81	***0.37***	*0.94*	*0.94*	0.92	0.00	0.96	1.46	0.97	0.87
Rmet_3287	*corA_3_*	**114**	**853**	0.13	*0.92*	***0.48***	*0.96*	*1.05*	1.20	1.11	1.18	1.39	0.99	0.30
Rmet_2621	*zupT*	**151**	61.0	2.48	0.72	*0.86*	1.63	*1.17*	1.02	1.19	1.11	1.04	0.78	0.86
Rmet_1533	*hoxN*	**183**	*5.67*	**32.3**	*1.36*	0.59	*0.98*	*0.94*	0.82	0.82	0.57	1.50	0.71	1.00
Rmet_1973	*pitA*	**280**	99.3	2.82	*0.34*	1.66	*1.05*	*1.01*	1.28	0.82	**2.44**	0.87	1.09	1.26
Rmet_5396	*mgtA*	12.3	*1.67*	7.40	1.38	*0.60*	*1.05*	***0.20***	0.95	0.00	0.69	**2.67**	0.97	2.00
Rmet_2211	*mgtB*	19.7	*2.00*	9.83	*1.05*	**14.7**	*1.12*	**2.17**	0.98	1.67	0.98	1.15	0.97	1.23
Rmet_0549	*zntB*	12.7	*2.00*	6.33	0.58	1.67	*0.97*	*0.83*	1.00	1.00	1.05	0.30	0.95	1.40
Rmet_5890	*feoB*	**110**	14.3	7.70	1.43	0.81	1.21	*0.91*	1.04	0.93	0.95	0.77	0.91	0.00
Rmet_5891	*feoA*	**132**	14.3	9.19	1.33	0.81	*1.24*	*0.91*	0.92	0.93	1.06	0.77	0.84	1.18
Efflux systems														
Rmet_4594	*zntA*	41.7	77.3	0.54	**73.2**	**5.54**	***0.40***	1.17	1.23	0.55	***0.25***	***0.34***	1.39	0.79
Rmet_2303	*cadA*	10.3	50.0	0.21	**62.1**	1.89	***0.48***	*1.01*	1.19	0.81	**0.08**	0.75	1.27	0.87
Rmet_5947	*pbrA*	*8.0*	*1.67*	4.80	**30.0**	**3.00**	0.75	*0.80*						
Rmet_5970	*czcP*	*7.7*	*0.67*	11.5	**9.74**	**840**	*1.13*							
Rmet_3524	*cupA*	15.0	47.3	0.32	**126**	**10.0**	*1.07*	*0.82*	1.00	0.77	***0.42***	**2.42**	0.94	0.82
Rmet_6119	*copF*	41.3	10.0	4.13	**13.4**	**8.97**	*1.00*	**2.00**						
Rmet_2211	*ctpF*	19.7	*2.00*	9.83	*1.05*	**14.7**	*1.12*	**2.17**	0.98	1.67	0.98	1.15	0.97	1.23
Rmet_2046	*rdxI*	250	*5.67*	**44.2**	***0.41***	1.29	*1.00*	*1.06*	1.08	0.59	1.10	0.86	1.08	0.72
Rmet_5979	*czcD*	13.7	*1.33*	**10.2**	**27.9**	**119**	***0.46***	*1.25*						
Rmet_0198	*dmeF*	44.3	**1734**	0.26	1.32	0.77	*0.89*	*1.02*	1.00	0.93	1.05	1.02	0.92	0.93
Rmet_3406	*fieF*	108	30.7	3.52	**2.43**	**5.37**	1.23	*0.99*	0.98	0.78	0.53	***0.45***	0.94	0.96
Rmet_6211	*cnrT*	47.3	21.3	2.22	**4.86**	*0.94*	*1.04*	0.78						
Rmet_0391	*atmA*	58.0	42.3	1.37	**3.68**	***0.47***	*1.09*	*0.90*	1.01	0.59	***0.40***	1.68	0.88	1.11

The locus tag, gene name, NPKM values of the sense and antisense transcripts is shown followed by the ration S/AS of both values. Bold-faced are here transcripts with NPKM >100 (<10 in italics) or S/AS ratios >10, which indicates a low probability of an antisense influence. The comparisons are metal-challenged to non-challenged CH34 cells, EDTA-treated to non-treated CH34 cells, unchallenged, metal-, and EDTA- challenged AE104 to CH34 cells. The ratios Q of the sense and antisense NPKM values is shown. Bold if Q >2, italics and bold if Q <0.5. Deviations are in the Supplementary Material M2, “Transport”. Empty cells indicate missing sense or antisense transcripts, for instance for plasmid-encoded factors in the plasmid-free strain AE104.

In the comparison of non-challenged cells of the plasmid-free strain AE104 to the wild-type strain CH34, expression of only 15 genes was up-regulated (*Q* > 2, *D* > 1, gene annotated) and that of 5 genes was down-regulated. Expression of the genes for the metal efflux and uptake systems was not different in both strains (Table 8). With a few exceptions, the plasmid-encoded metal resistance determinants had little impact on cells living under non-challenging conditions.

When metal-challenged AE104 and CH34 cells were compared, 305 genes were up-regulated and 159 genes were down-regulated in AE104 compared to CH34 (Table 4). A total of 230 of the 305 genes up-regulated in AE104 was down-regulated (*Q* < 0.7) in metal-shocked CH34 cells. Included is a variety of genes that were down-regulated in the comparison of metal-shocked CH34 compared to untreated CH34 cells: 75% of the up-regulated genes in AE104 nearly reached the same transcript abundance in metal-stressed AE104 cells as in non-challenged CH34 cells. This means that the plasmid-encoded metal resistance determinants were responsible for the energy shortfall and its consequences in strain CH34.

In 38 cases, genes already up-regulated in metal-stressed CH34 cells were expressed at an even higher abundance in metal-stressed AE104 cells. These were components of the chromosomal or chromid-encoded metal resistance determinants *cus, cop_2_* (Table 6), and especially *mer*, which was up-regulated already 4000-fold in CH34 and now again additionally 2-fold in AE104 when the plasmid-encoded *mer* determinants were absent (Supplementary Material M2). In AE104 cells, with the exception of the gene encoding the response regulator HmzR, no component of the recessive RND-driven trans-envelope systems was up-regulated above the level determined for metal-challenged CH34 cells. Clearly, these systems did not compensate for the plasmid-encoded systems. There was also no change in the expression of the genes for the metal uptake systems, with one exception: *pitA* was 0.34-fold down-regulated in metal-challenged CH34 cells and 2.44-fold up-regulated in metal-challenged AE104 cells (Table 8), nearly reaching the abundance of the transcript in un-challenged CH34 cells. The antisense activity of *corA_1_* was 50-fold up-regulated in metal-challenged AE104 cells.

Comparing the abundances of the efflux systems in metal-challenged AE104 to non-challenged AE104 control cells, the genes *zntA, cadA, cupA*, and *atmA* were up-regulated approximately 18-, 5-, 53-, and 1.5-fold, respectively. The differences in the degree of up-regulation between CH34 and AE104 cells agreed with the lower concentrations of the challenging metals (Table 2). Despite the lower concentration of challenging metal ions and the absence of the plasmid-encoded metal resistance determinants, sufficient zinc, copper, and cadmium reached the cytoplasm of strain AE104 to yield a ZntR-, CadR-, and CupR-dependent up-regulation of *zntA, cadA*, and *cupA*, respectively. There was no change in the expression of the genes encoding sigma factors but an up-regulation of the genes encoding the RNAP-associated proteins NusG and GreA was determined. The antisense activities of three sigma factor genes were down-regulated (*rpoD_1_* for the main housekeeping sigma factor, *rpoJ, rpoO)* and two were up-regulated (*rpoN, rpoM*). No genes were found that were not expressed in metal-challenged CH34 cells but, which were expressed in metal-challenged AE104 cells. No additional genes whose products might have compensated for loss of the plasmid-encoded metal resistance determinants, for instance, in recessive RND-encoding determinants. Due to the lower concentration of the challenging metals used, the genes of non-plasmid-encoded resistance determinants were even less well up-regulated in strain AE104 compared to the wildtype, CH34. This agrees with the observation that strain AE104 simply loses metal resistance when the plasmids pMOL30 and pMOL28 are lost [6].

Finally, in the comparison between metal-starved AE104 and CH34 cells, expression of only seven genes was up-regulated in the comparison between EDTA-treated AE104 and CH34 cells (Table 4). There was no change in the sense transcripts or antisense activities for the genes encoding the metal uptake or efflux systems (Table 8), the porins, the TonB-dependent receptors except Rmet_0123 or the ABC transport systems. However, changes in the antisense activities were noted (Supplementary Material M2). The only noted expression changes concerning genes encoding the RNAP and its sigma factors were changes in the expression of antisense activities for some sigma factors. The antisense activities for *rpoN*, which had been up-regulated in the comparison between metal-shocked cells, was down-regulated in the comparison between EDTA-treated cells, while the antisense transcript associated with *rpoP* displayed the inverse response. The sense or antisense transcript for *rpoO* was unchanged in CH34 cells but its antisense showed a metal-dependent titration pattern, being completely absent in the comparison between metal-treated cells, 0.25-fold down-regulated in non-challenged cells and 0.33-fold down-regulated in EDTA-treated cells. This indicated that antisense transcripts may be involved in control of the expression of sigma factor genes in *C. metallidurans*.

### Independent evidence for the existence of asRNAs

**Figure 2.**
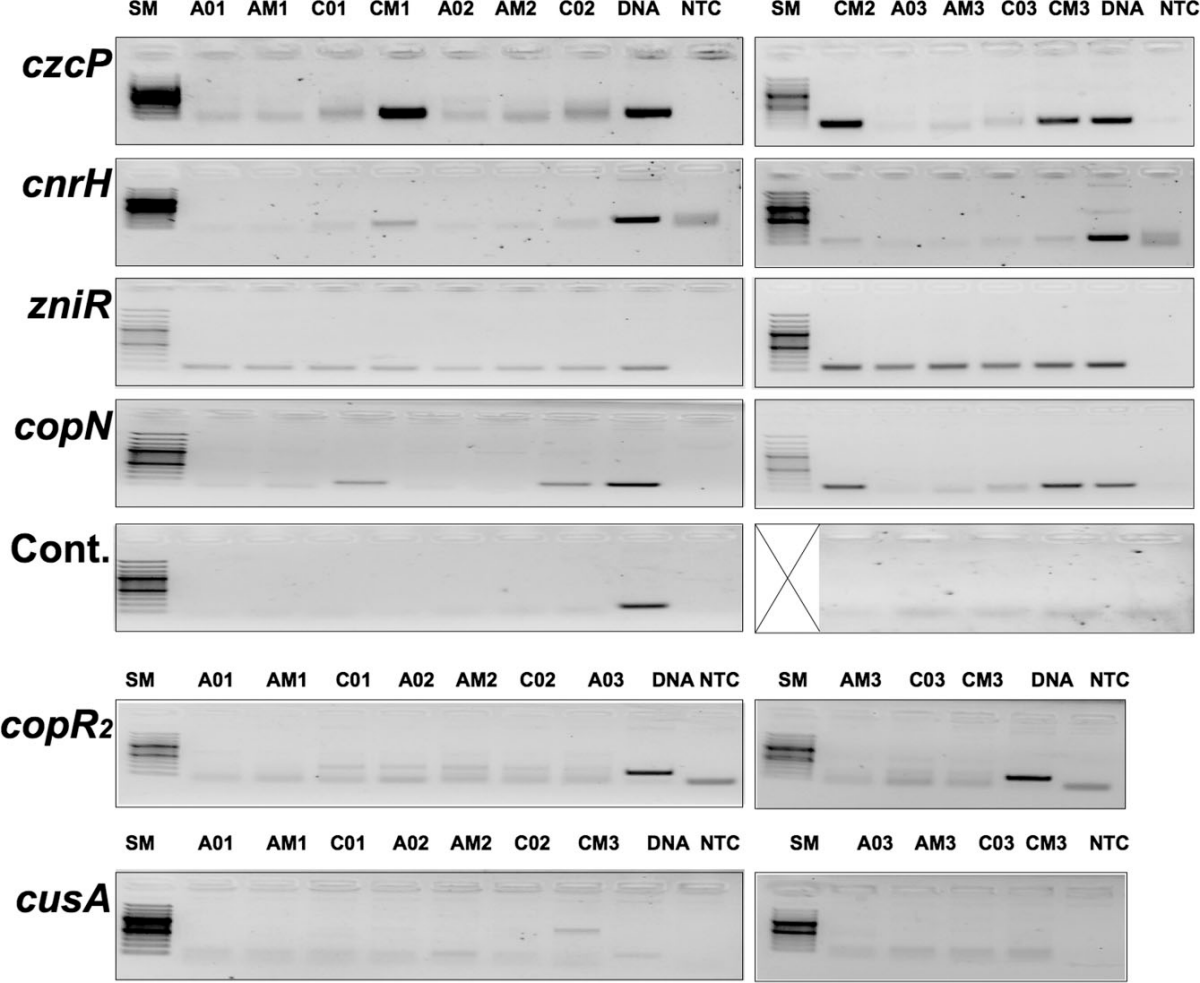
Demonstration of the existence of asRNA using RT-PCR. RNA prepared from *C. metallidurans* strains AE104 (A) or CH34 (C) challenged with metals (AM, CM) or not (A0, C0) were reversely transcribed using RT primers for asRNAs and subsequently amplified by PCR. Controls are DNA, not template (NTC) or for DNA contamination (Cont.). Three experiments. One control does not show the size marker (Lane crossed-out).

Six genes were selected to verify the existence of asRNAs by RT-PCR (Fig. 2). These were the genes encoding the P_IB4_-type ATPase CzcP, the sigma factor CnrH, the response regulators ZniR and CopR_2_, the copper resistance CopN and the RND protein CusA. Moreover, transcriptional start sites (TSS) were determined as published [22] using RNAs from the same cells that were used for the RNASeq experiments.

**Figure 3.**
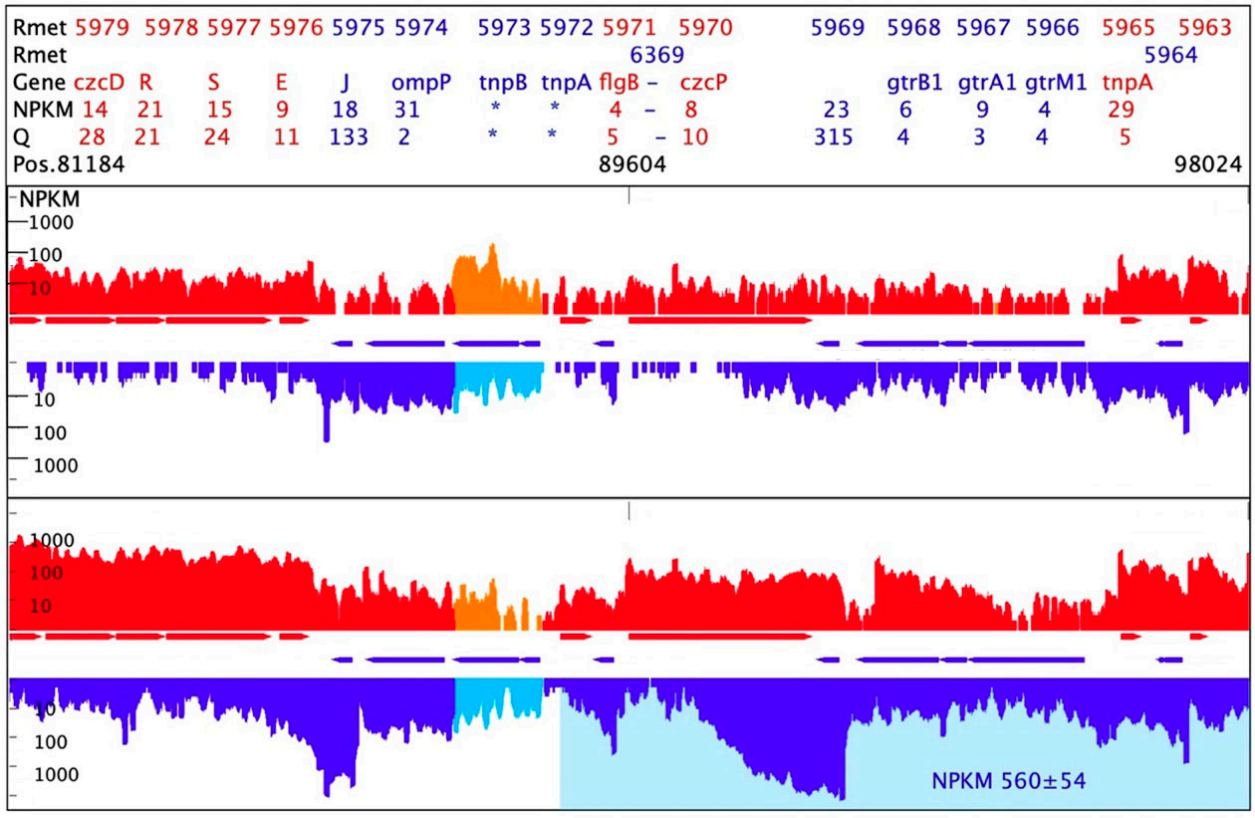
Map of the *czcP* gene region in *C. metallidurans* strain CH34. This map summarizes the data from the RNASeq experiment. The map shows the indicated determinant with NPKM values on one DNA strand (red) or the other direction of transcription (blue) from triple determinations with deviations. The transcript abundances are from one of these experiments. The header gives the position on the chromid, the Rmet locus, and gene names. Shaded areas are annotated antisense transcript regions with their NPKM values. Top, unchallenged CH34 cells; bottom, CH34 with Multitox metal mix.

In the case of the *czcP* gene, antisense transcription was up-regulated 76-fold from an initial NPKM value of 7.33 ± 0.21 to 560 ± 54 (Fig. 3, Table 9, Table S5). This up-regulation was mirrored by the RT-PCR experiment (Fig. 2). The intensity of the *czcP-*asRNA signal was in all three determinations stronger than the DNA control but barely visible when RNA from unchallenged cells of *C. metallidurans* CH34 was used. Since *czcP* is part of the *czc* resistance determinant on plasmid pMOL30, no *czcP-*asRNA-signals could be identified using RT-PCR with RNA from the plasmid-free strain AE104. Faint bands visible here were at the position and intensity of the negative no-template control (Fig. 2, NTC). The annotated asRNA in metal-challenged CH34 cells initiated 8,854 bp downstream of the 3′ end of the 3URT of *czcP* and continued up to 790 bp upstream of the 5UTR of this gene. In contrast, non-challenged CH34 cells contained two shorter asRNAs with low intensity (Table 9, Table S5).

**Table 9.** Transcriptional details of the *czcP* gene and its environment*^a^*.

**Element**	**start**	**strand**	**stop**	**Length**	**Locus Tag/Prom.**	**Signal C0**	**Signal CM**
**TSS_88714 + 4**	88714	+			NotPub , CH34_M	n.f.	46 ± 7
*STP_AST_101723-4*	88625	-	88625	13098			560 ± 54
*STP_AST_89550-4*	88711	-	88711	839		7 ± 2	
**5UTR_5970**	89415	+	89604	189	Rmet_5970, *czcP*	6 ± 1	n.f.
**TSS_89553+4**	89553	+			NotPub , CH34_M	24 ± 6	1202 ± 179
**TSS_89563+4**	89563	+			Pub, *no ass.*	129 ± 15	2639 ± 271
*TSS_89441-4*	89441	-			Pub, RpoD	n.f.	478 ± 426
*AST_89550-4*	89550	-	88711	839		7 ± 2	
*TSS_92052-4*	92052	-			NotPub , CH34_0	23 ± 3	n.f.
**NPKM_5970**	89604	+	92094	2490	Rmet_5970, *czcP*	8 ± 2	75 ± 1
*STP_AST_92956-4*	90958	-	90958	1998		14 ± 2	
			
RT-PCR_czcP	91841		91978	137			
			
**3UTR_5970**	92095	+	92869	774	Rmet_5970, *czcP*	6 ± 1	32 ± 2
*TSS_92062-4*	92062	-			NotPub , CH34_0	27 ± 6	n.f.
*TSS_92535-4*	92535	-			Pub, RpoD	277 ± 12	167358 ± 14211
*AST_92956-4*	92956	-	90958	1998		14 ± 2	.
*TSS_96132-4*	96132	-			Pub, *no ass.*	280 ± 49	1168 ± 150
*TSS_97054-4*	97054	-			Pub, *no ass.*	669 ± 206	2395 ± 828
*TSS_97146-4*	97146	-			NotPub , CH34_M	74 ± 4	125 ± 12
*TSS_97206-4*	97206	-			Pub, RpoD	2718 ± 642	12288 ± 1457
*TSS_98167-4*	98167	-			NotPub , CH34_M	129 ± 12	380 ± 30
*TSS_98671-4*	98671	-			Pub, *no ass.*	149 ± 76	319 ± 35
*TSS_98681-4*	98681	-			NotPub , CH34_M	21 ± 1	80 ± 14
*TSS_99109-4*	99109	-			NotPub , CH34_M	33 ± 4	18 ± 1
*TSS_100199-4*	100199	-			Pub, *no ass.*	1437 ± 309	3355 ± 308
*TSS_100887-4*	100887	-			Pub, (RpoD)	368 ± 43	881 ± 112
*TSS_101020-4*	101020	-			Pub, *no ass.*	270 ± 92	2898 ± 91
*TSS_101042-4*	101042	-			NotPub , CH34_M	77 ± 11	319 ± 27
*AST_101723-4*	101723	-	88625	13098			560 ± 54
*TSS_103614-4*	103614	-			NotPub , CH34_E	23 ± 5	n.f.

a The table gives the details of the transcripts of the *czcP* region on plasmid pMOL30 as determined by RNASeq. The elements are the transcriptional start sites TSS, the asRNAs AST and their respective stop position STP_AST, the genes NPKM and 3UTR for an untranslated region. Bold indicates transcripts from the ‘+’ strand, italics from the “-” strand. The boxed area indicates the RT-PCR product from the asRNA of that region. Shaded asRNAs were measured in RNA isolated from metal-induced cells of *C. metallidurans* CH34, others from cells not treated with metals. The locus tag and names of the genes are also provided. Published TSS (REF) are given with the sigma factor responsible for transcription initiation, here RpoD when the promoter was strongly correlated with the RpoD model, otherwise (RpoD), ‘*no ass.’* means no association with the RpoD model. The last two rows contain the intensity of the signals, NPKM values for the genes, 3UTR and asRNAs and scores for the TSSs in non-treated *C. metallidurans* cells C0 or metal-treated cells CM; n.f. no signal found. Three determinations. When for a TSS one value is given, this means that only one signal appeared in the three experiments.

Three transcriptional start sites were found upstream of *czcP* (Table 9). Two of them in the new determination and here identified in metal-challenged CH34 cells. One reached only a score of 46 ± 7 in these cells and was located 1,240 bp upstream of the 5UTR of *czcP,* the other was within the 5UTR 51 bp upstream of the *czcP* gene. This TSS possessed a score of 1,202 ± 179 in metal-challenged cells. A third TSS was only 10 bp apart and 41 bp upstream of *czcP*, with a score of 2,639 ± 271 in metal-challenged CH34 cells. The respective promoter was classified as a non-RpoD promoter [22]. A large number of TSS existed for the antisense direction, both previously identified [22] and newly identified ones, some of which were associated with the RpoD promoter model, others not (Table 9, Table S5). Many of them were up-regulated in metal-challenged CH34 cells. There was no TSS directly upstream of the long, and strongly expressed, *czcP*-antisense transcript. The closest upstream TSS was 1,891 bps away and not found in metal-challenged CH34 cells. While two promoters were clearly responsible for *czcP* transcription in metal-challenged CH34 cells, the ASTs associated with *czcP* were the sum of many transcriptional events on the respective other DNA strand, leading to a group of overlapping transcripts (Table 9, Table S5, Fig. 3). The *czcP-*associated ASTs comprised a bin, but, nevertheless, the existence of *czcP-*associated asRNAs could be clearly demonstrated by RT-PCR (Figs 2 and 3).

The results for the *cnrH* gene were reminiscent of those for the *czcP* gene, but the signals in the RT-PCR experiments were much weaker. RT-PCT signals were visible when the RNA was derived from metal-challenged CH34 cells, very weak when from non-challenged CH34 cells, and absent in the case of AE104 cells because *cnrH* is located on plasmid pMOL28 (Fig. 2). One promoter was responsible for *cnrH* transcription, one *cnrH*-asRNA was annotated in metal-challenged cells and a shorter and weakly scoring asRNAs in non-challenged cells (Table 10, Table S5). The *cnrH*-asRNA initiated within the *cnrC* gene and extended across *cnrYXH* with a length of 12,994 bps. As for *czcP*, no clear TSS could be associated with this *cnrH-*asRNA, but a variety of transcriptional initiation events occurred in the direction that was antisense to *cnrH.* As in the case of *czcP*, the ASTs were bins of transcripts rather than discrete asRNAs but *cnrH*-specific asRNAs were within this bin.

**Table 10.** Transcriptional details of the *cnrH* gene and its environment^a^.

**Element**	**Start**	**Strand**	**Stop**	**Length**	**Locus Tag/Prom.**	**Gene**	**Signal C0**	**Signal CM**
*STP_AST_56260-5*	43266	-	43266	12994				1,083 ± 159
**TSS_53286+5**	53286	+			Pub, RpoD		2,918 ± 1,624	2,8233 ± 2,977
**NPKM_6205**	53309	+	53597	288	Rmet_6205	*cnrY*	171 ± 56	1,081 ± 178
*STP_AST_54298-5*	53435	-	53435	863			3	
**NPKM_6206**	53593	+	54040	447	Rmet_6206	*cnrX*	99 ± 30	726 ± 116
**NPKM_6207**	54036	+	54612	576	Rmet_6207	*cnrH*	61 ± 14	466 ± 101
RT-PCR_cnrH	54162		54287	125				
*AST_54298-5*	54298	-	53435	863			3	n.f.
*TSS_54822-5*	54822	-			NotPub, CH34_M		n.f.	50 ± 16
**NPKM_6208**	54684	+	55941	1257	Rmet_6208	*cnrC*	48 ± 13	255 ± 30
*TSS_54955-5*	54955	-			NotPub, CH34_M		n.f.	23 ± 5
*TSS_56237-5*	56237	-			Pub, RpoD		32	n.f.
*AST_56260-5*	56260	-	43266	12994				1,083 ± 159
**NPKM_6209**	55937	+	57125	1188	Rmet_6209	*cnrB*	26 ± 5	150 ± 20
**NPKM_6210**	57121	+	60352	3231	Rmet_6210	*cnrA*	30 ± 5	168 ± 10
*TSS_57840-5*	57840	-			NotPub, CH34_E		30 ± 16	0 ± 0
*TSS_58867-5*	58867	-			Pub, RpoD		158 ± 12	212 ± 37
*TSS_59074-5*	59074	-			NotPub, CH34_M		25 ± 6	107 ± 24
*TSS_60287-5*	60287	-			NotPub, CH34_M		81 ± 14	231 ± 20
**NPKM_6211**	60397	+	61453	1056	Rmet_6211	*cnrT*	47 ± 8	230 ± 5
*TSS_60510-5*	60510	-			NotPub, CH34_M		29 ± 6	102 ± 24
*TSS_60580-5*	60580	-			NotPub, CH34_M		45 ± 12	109 ± 30
**3UTR_6211**	61454	+	63472	2018	Rmet_6211	*cnrT*	12 ± 1	29 ± 5
*TSS_63507-5*	63507	-			NotPub, CH34_M		35 ± 6	51 ± 7

a The table gives the details of the transcripts of the *cnr* region on plasmid pMOL28 as determined by RNASeq. The elements are the transcriptional start sites TSS, the asRNAs AST and their respective stop position STP_AST, the genes NPKM and 3UTR for an untranslated region. Bold indicates transcripts from the “+” strand, italics from the “-” strand. The boxed area indicates the RT-PCR product from the asRNA of that region. Shaded asRNAs were measured in RNA isolated from metal-induced cells of *C. metallidurans* CH34, others from cells not treated with metals. The locus tag and names of the genes are also provided. Published TSS (REF) are given with the sigma factor responsible for transcription initiation, here RpoD. The last two rows contain the intensity of the signals, NPKM values for the genes, 3UTR and asRNAs and scores for the TSSs in non-treated *C. metallidurans* cells C0 or metal-treated cells CM; n.f. no signal found. Three determinations. When for a TSS one value is given, this means that only one signal appeared in the three experiments.

Antisense transcripts could be verified for the chromid-encoded *zniR* gene in RNA from all cells, metal-challenged and not challenged CH34 and AE104 cells, with only minor differences in the band intensities (Fig. 2). As in the case of *cnrH* and *czcP*, RNA from metal-treated CH34 cells contained a longer and more abundant asRNA than RNA from non-treated CH34 cells. One TSS could be assigned to the sense transcription of *zniR* but no TSS to the *zniR*-asRNAs. A TSS was located 2,618 bp upstream of the start position of the asRNA in metal-treated CH34 cells and thus is probably not responsible for its transcription.

The presence of an asRNA for the plasmid-encoded *copN* gene could be demonstrated in RNA from metal-challenged CH34 cells and with lower intensity in RNA from non-treated CH34 cells. Signals were absent when the RNA came from cells of the plasmid-free strain AE104 (Fig. 2). Several promoters upstream of *copN* were up-regulated in metal-challenged CH34 cells and were likely responsible for *copN* transcription, or that of the genes *copR_1_* and *copS* upstream of *copN* (Fig. S4). As in the other cases, the annotated asRNA from metal-challenged CH34 cells had a higher abundance and was longer than the two asRNAs from non-challenged cells. Several promoters in the antisense direction were up-regulated in metal-challenged CH34 cells but none was identified directly upstream of the start position of the asRNA up-regulated in metal-challenged CH34 cells.

Only faint RT-PCR signals were associated with asRNAs for the chromid-encoded *copR_2_* and *cusA* genes. This agreed with the observation that, in contrast to the plasmid-encoded genes, these two determinants, which are not plasmid-encoded, were accompanied by down-regulated or unregulated antisense transcripts (Table 6). In metal-challenged CH34 cells, the *copR_2_-*asRNA had a higher abundance and was longer compared to the asRNA from non-challenged cells (Table S5, Fig. S4). The abundances for both *copR_2_*-RNAs were comparably low, which explains the weak signals in the RT-PCR experiment.

The pattern for *cusA* was different from that of the other five genes (Table S5, Fig. S5). In RNA from non-challenged CH34 cells, a large 6,794 bp asRNA with a low abundance of NPKM = 35 ± 5 extended across several genes in the sense direction, including *cusA*. In non-challenged cells, an asRNA with a 2-fold higher abundance of NPKM = 66.7 ± 7.1 initiated 24 bp upstream of the asRNA from metal-challenged cells, but extended across a shorter distance of 4,202 bps, ending within *cusA*, 828 bps from the start of the 3 kb *cusA* open reading frame (ORF). A second asRNA followed but had a very low abundance of only NPKM = 4.7 ± 0.6. As in the case of the other five genes, no TSSs could be associated with the asRNAs but several within the annotated asRNA region were identified, with many of them up-regulated after metal treatment in *C. metallidurans* strain CH34 (Table S5). More details concerning the changes in the sense and antisense transcripts in all these comparisons, especially in the transcriptome of the *cop_2_* and *cus* copper resistance determinants, are provided in the Supplementary Information section.

The RT-PCR experiments gave independent evidence for the existence of asRNA for the six example genes. While one or more TSSs indicated the presence of promoters that were responsible for sense transcription of these genes, no TSSs were found directly upstream of the annotated ASTs. The annotated ASTs were obviously bins of transcripts, including the respective asRNAs. The transcription initiation events on the respective other DNA strand were probably not terminated but extended instead as antisense transcripts into the region of the ORF of the gene under consideration.

## Discussion

### The sense of antisense

The RNASeq technology has allowed the identification of a multitude of interacting RNA molecules in the bacterial cell. Starting with a few examples 40 years ago [35], now non-coding, small regulatory, and antisense RNAs seem to be widely distributed [36–40]. Non-coding RNAs (ncRNAs) different from tRNAs and rRNAs can be *cis-* or *trans*-acting ncRNAs [36]. The *trans*-acting ncRNAs act upon one or several genes at a distance. *Cis*-acting ncRNAs exist as long antisense RNAs (asRNAs) spanning several genes, which may even vary between the sense and the antisense orientation, or shorter antisense RNAs that may overlap with other RNAs at the 5′ end, the 3′ end or may be located internally between or within the ORFs of genes [37]. The excludon concept [41] defines “excludons” as a genomic locus encoding a long asRNA that spans divergent operons encoding products with related or opposing functions. The “dual-use” RNAs identified in *C. metallidurans* adds to the accumulating evidence for this concept. RNAs may start as sense mRNA, extend as asRNA into 5′ parts or parts of the ORFs of adjacent genes on the other DNA strand and may even become sense RNAs again.

Such pervasive transcription may have numerous consequences. Sense and asRNA may form double stranded RNA that may lead to co-degradation by RNAseIII or stabilization of the sense RNA due to inhibition of RNAse E decay. Moreover, the asRNA or the synthesis of it by transcription may lead to promoter exclusion with respect to another RNA, collision of RNA polymerases (RNAP), or a “sitting-duck”, removal of an RNAP that has not yet escaped from the promoter [38]. An asRNA could serve as a “sponge” to titrate its sense RNA, cause termination or interfere with translation initiation. These mechanisms are especially useful for homeostasis and stress response conditions because the sense-antisense interaction defines a low-cost energetic mechanism to mediate a threshold of the response or allows fine-tuning of the expression of several genes [38, 39]. Sudden stress conditions require an immediate response to protect the cell. A rapid expression of a variety of genes needs control of the temporal fine-tuning, regulation of the various gene products and even that of the subunits of hetero-multimeric protein complexes. Additionally, expression of the multitude of the housekeeping genes has to be adapted to synchronize their function with those of the stress defenders. This explains why the largest number of regulated ASTs appears in *C. metallidurans* under metal-shock conditions (Table 3). Due to the method used [28], these ASTs may represent a discrete transcript or a bin of transcripts from the region annotated as “AST”, so that the asRNA could be an independent molecule, part of an RNA containing both sense and antisense functions, or a member of a group of transcripts from the respective region.

Only 1,319 ASTs with an abundance of NPKM > 10 and a length >50 bp were found in the *C. metallidurans* transcriptome, while the total number of ASTs was 10 757. This could be explained with transcription noise [42]. On the other hand, studies on the transcriptome of *Staphalococcus aureus* suggest that hidden asRNAs may sponge-up their sense RNAs, leading to RNAseIII-dependent degradation [43] and subsequently to appearance of small asRNAs in the transcriptome as products of this process. Such a process may be involved in fine-tuning of gene expression but also in global regulatory processes such as RNA degradation or transcription-dependent DNA repair [37]. If such a process exists in *C. metallidurans*, even low abundance asRNAs should have a direct influence on the resulting copy numbers of the respective gene products because they signal the existence of hidden asRNAs.

In bacteria, transcription occurs in the nucleoid, which is located in a defined region in the middle of the cytoplasm [44, 45]. The small ribosomal subunit is also present within the nucleoid [46, 47], so that one “leader” ribosome may bind immediately to the transcribing RNA polymerase, forming an “expressome” protein complex [48, 49]. This allows regulatory mechanisms such as attenuation [50]. The majority of the ribosomes are located at the border between the nucleoid and the remaining cytoplasm, so that for strong gene expression events, the respective genes have to move to the surface of the nucleoid [51]. This has also the advantage of short diffusion distances for nucleoside triphosphates for transcription including GTP for translation, and amino acids for synthesis of the amino-acylated tRNAs for protein synthesis by the ribosome.

In this way, a sense mRNA may be immediately covered by one ribosome or more. Should that not be the case, the Rho factor can bind to *rut* (Rho utilization) sites at the mRNA and follow it toward the transcribing RNA polymerase (RNAP). When it reaches the RNAP, transcription is terminated [32, 33]. Rho enforces coupling of transcription and translation and decreases the probability of the occurrence of unoccupied single stranded RNA. Although the small ribosomal subunit may be present within the nucleoid [46, 47], internal sense RNAs could be produced before Rho was able to act but could not be translated due to a lack of ribosomes within the nucleoid. Interaction with an asRNA could be useful to decide the fate of these RNAs, co-degradation or stabilization until a ribosome appears. On the other hand, asRNA-mediated co-degradation could be an important catalytic mechanism to recycle the RNA components from already translated mRNAs at the border between nucleoid and cytoplasm [37].

Simultaneous transcription of both DNA strands in the sense and antisense direction may lead to head-on collision of the transcribing RNAPs. The number of RNAPs in the *C. metallidurans* cell is about 5,000 [52]. About 10% of the RNAP is usually actively transcribing [53]. With 6,000 genes in *C. metallidurans* [2] giving 12 000 when the antisense events on the respective opposite DNA strand are included and 500 actively transcribing RNAPs, the probability of a gene or antisense transcript for being actively transcribed is 4.17% and thus the head-on collision probability (4.17%)^2^ = 0.17%. Head-on collisions should be rare events, occurring only in strongly expressed genes with strong antisense transcription. On the other hand, head-on collisions may also occur at a long distance [38] so that this mechanism cannot be excluded.

Antisense activities were up-regulated for most metal-resistance genes in metal-shocked *C. metallidurans* CH34 cells. In 696 cases, metal shock resulted in the up- or down-regulation of asRNAs in CH34 cells (Table 3). These numbers were comparable numbers to results from *Pseudomonas aeruginosa*. Here, of 4,993 genes with asRNAs, 298 had an asRNA differentially regulated under various environmental conditions with 186 associated with an up- or down-regulated sense transcript [54]. Interestingly, 45 genes in this bacterium, each with an antisense transcript, were modulated by alternative sigma factors. *Cupriavidus metallidurans* possesses a variety of alternative sigma factors but studies with mutants and gene arrays did not yield a clear picture of their specific physiological functions [55]. These sigma factors may just modulate gene expression by initiation of antisense transcription and, as shown here, asRNAs might regulate expression of genes encoding sigma factors and anti-sigma factors. This would lead to a new level of regulatory processes. External signals may control the release of a sigma factor from its membrane-bound antis-sigma factor. These may subsequently bind to the RNA polymerase to form the respective holoenzyme, which initiates transcription of interacting sense, antisense RNAs and *trans*-acting RNAs. These RNAs may interact to modulate gene expression, including those for other sigma factors.

In metal-shocked *C. metallidurans* cells, genes involved in translation, transcription, tRNA synthesis and energy conservation were down-regulated after 10 min (Fig. 1). Following metal shock, expression of the genes for metal efflux pumps was induced. The resulting proteins may compete with those for the F_1_F_0_ ATPase and respiratory chain components for space in the inner membrane and for the approximately 1 000 proteins required for protein export and membrane insertion, mainly SecY, Ffh for the signal recognition particle, the receptor FtsY and the alternative insertion pathway YidC [56]. Due to the limited protein export capacity, this competition may lead to a down-regulation of gene expression for those encoding the F_1_F_0_ ATPase and respiratory chain components (Fig. 1), either in the long or the short term. In the long term, a higher demand for energy to drive the metal efflux pumps in combination with the decreased ability to conserve energy as ATP or proton motive force would result in some degree of energy starvation. As a solution to this problem, systems with a high energy demand are also down-regulated, *e.g*. genes encoding components of the motility and protein synthesis apparatuses. It should be noted that down-regulation of all these components is much stronger in metal-shocked CH34 cells compared to metal-shocked AE104 cell, so that the plasmid-encoded large trans-envelope efflux pumps CzcCBA and CnrCBA may be responsible for space and export competition and dealing with a subsequent mild ‘energy crisis’. Being able to survive in the presence of high zinc, cobalt and nickel concentrations is a resource-intensive problem for *C. metallidurans* CH34. This also explains the presence of asRNA in these plasmid-encoded metal resistance determinants, which may prevent an overshooting expression of these genes.

Alternatively to this scenario, a rapid onset of metal shock needs the immediate expression of defense genes so that RNA polymerases are titrated away from their usual house-keeping promoters. The RNA for this study was isolated 10 min after metal shock was initiated because the mRNA for proteins involved in metal resistance appeared after 2–20 min in *E. coli* or *C. metallidurans* following metal shock [57, 58], while RNA abundance decreased after this time period. During this short time period, decreased expression of ribosomal and other house-keeping genes may not have resulted in a strong decrease of the abundance of the respective gene products. Should the number of these housekeeping proteins remain low after a longer period, this would indicate that the production of the defense systems causes a continual burden to the cell. Should there be a comparable small effect on the abundance of the housekeeping proteins, this would indicate that upon metal shock, expression of the housekeeping genes was repressed to allow recruitment of the RNA polymerase, ribosomes, and protein export capacity for expression of the defense gene with a subsequent recovery of the cells. The observation that the response regulator CzcR of a *czc* determinant in *P. aeruginosa* is required to regulate expression of flagellar genes suggests direct control of energy-consuming cellular function by metal resistance genes [59]. In agreement with this assumption, metal shock did not result in an overall global decrease of expression of the housekeeping genes, but instead for only selected groups of them, and only in the plasmid-containing strain CH34. Production or operation of the plasmid-encoded metal resistance systems, e.g. the CzcCBA and CnrCBA transenvelope efflux complexes may have resulted in a down-regulation of the described groups of housekeeping genes to allow their translation and membrane-insertion. Proteomics may reveal whether the production or the operation of these factors was the main reason for the observed effects.

A change in expression in the gene for the starvation sigma factor RpoS was not observed under metal-shock conditions (Table 7), which would mean that the observed effect was not a starvation response. On the other hand, a number of regulatory circuits affect translation of the *rpoS*-mRNA and stability of the protein [60], so that a starvation response cannot be fully excluded without consideration of proteomic data.

In some respects, pervasive transcription from both DNA strands and the formation of transient double-stranded RNAs could be a relic of the ancient past. DNA as the information-storage macromolecule likely evolved at a later stage in evolution of the last common ancestor LUCA [61] and interestingly, nucleus-like structures are still appearing during viral replication in bacteria [62]. In a time before DNA, RNA might have been the main genetic storage material and DNA may have subsequently evolved as chemically stable form to allow horizontal gene transfer. Single-stranded RNA folds into hairpins as secondary and more complicated tertiary conformations, for instance, the rRNAs in the translating ribosome [63]; however, folded RNA is probably difficult to use as mRNA. Consequently, the function of an antisense RNA could be to prevent folding of an mRNA and keep it accessible for translation. Evolvement of RNAse III subsequently diverged the asRNA world into those possessing recognition sites for this enzyme, leading to co-degradation, and those which bind and protect their sense RNAs. All these elements of the bacterial transcriptome seem to be needed to allow a rapid but synchronized and balanced response to stress conditions such as a metal shock.

## Materials and methods

### Bacterial strains and growth conditions

Plasmids and *C. metallidurans* strains in this study were strain CH34 wild type and its plasmid-free derivative AE104 that lacks pMOL28 and pMOL30 [6]. Tris-buffered mineral salts medium [6] containing 2 g sodium gluconate/L (TMM) was used to cultivate these strains aerobically with shaking at 30°C. Analytical grade salts of cation chlorides, potassium chromate, and potassium arsenate were used to prepare 1 M stock solutions, which were sterilized by filtration. Tris-buffered media were solidified by incorporating 20 g agar/l.

A previously used multi-metal mix [27] had been used to challenge *C. metallidurans* CH34 and AE104 simultaneously with several transition metal cations. This MultiTox metal mix was optimized for the transcriptome determination because the previously used mix did not address the different toxicities of the individual metals. Moreover, it did not discriminate between the more metal sensitive strain AE104 and its wild-type CH34, and did not contain arsenate, chromate, or mercury. For the optimization of the CH34- and AE104-specific MultiTox metal mixes, the IC_50_ values for the respective metals were again determined for *C. metallidurans* CH34 and AE104. Based upon these, strain-specific metal mixes were designed that contained the challenging ions in concentrations proportional to their IC_50_ values and the IC_50_ of this strain-specific mix was finally determined. The resulting IC_50_ value for the CH34-specific MultiTox metal mix was 3.35 mM, composed of 461 µM Zn(II), 241 µM Cu(II), 1,503 µM arsenate, 0.37 µM Hg(II), 19 µM chromate, 15 µM Cd(II), 761 µM Ni(II), and 347 µM Co(II). Strain AE104 was challenged with 1,000 µM of its specific MultiTox mixture comprised of 5 µM Zn(II), 120 µM Cu(II), 849 µM arsenate, 0.18 µM Hg(II), 7 µM chromate, 3 µM Cd(II), 9 µM Ni(II), and 7 µM Co(II)). These MultiTox metal mixes should lead to a comparable toxicity of each metal in the cells of the strains CH34 and in AE104, and lead to an up-regulation of all metal resistance determinants present in both strains.

### Inductively-coupled plasma mass spectrometry

Cells were incubated in TMM up to early stationary phase at 30°C and with shaking at 200 rpm, diluted 20-fold into fresh TMM medium and incubation continued at 30°C for 24 h. Cells were diluted 50-fold into fresh medium and incubation continued at 30°C at 200 rpm until 100 Klett units were reached, which was before the mid-exponential phase of growth. Where indicated, MultiTox metal mix or EDTA were added, and incubation was continued at 30°C and at 200 rpm until 150 Klett units were reached. Ten milliliters of the cells were harvested by centrifugation, washed twice with 50 mM Tris-HCl buffer (pH 7.0) containing 10 mM EDTA at 0°C and suspended in 50 mM Tris-HCl buffer (pH 7.0). For ICP-MS analysis, HNO_3_ (trace metal grade; Normatom/PROLABO) was added to the samples to a final concentration of 67% (w/v) and the mixture mineralized at 70°C for 2 h. Samples were diluted to a final concentration of 2% (w/v) nitric acid. Indium and germanium were added as internal standards at a final concentration of 1 ppb and 10 ppb, respectively. Elemental analysis was performed via ICP-MS using a Cetac ASX-560 sampler (Teledyne, CETAC Technologies, Omaha, Nebraska), a MicroFlow PFA-100 nebulizer (Elemental Scientific, Mainz, Germany) and an ICAP^TM^-RQ ICP-MS instrument (Thermo Fisher Scientific, Bremen) operating with a collision cell and flow rates of 4.5 ml x min^−1^ of He/H_2_ (93%/7% [64]), with an Ar carrier flow rate of 0.76 l x min^−1^ and an Ar make-up flow rate at 15 l x min^−1^. An external calibration curve was recorded with ICP-multi-element standard solution XVI (Merck) in 2% (v/v) nitric acid. The sample was introduced via a peristaltic pump and analyzed for its metal content. For blank measurement and quality/quantity thresholds, calculations based on DIN32645 TMM were used. The results were calculated from the ppb data as atoms per cell as described [27].

### Determination of Rho-utilization sites

The program RhoTermPredict was used to predict all possible *rut* sites in the genome of *C. metallidurans* CH34 using the gene bank entry CH34.gbk. This program gives for each possible *rut* site the DNA sequence, the downstream sequence and a list of one or more conserved sequence motifs with a score between 6 and 15 for each set of conserved motifs [65]. The *rut* sites were denominated for each replicon and DNA strand depending on the order of the start position, the “+” or “−” strand and the replicon number from “2” for the chromosome to “5” for plasmid pMOL28. For each *rut* site, the score of the best conserved sequence motif was noted. As the next step, the *rut* sites were assigned to a gene if they were located within or downstream of its ORF. For each gene, the number 5 was subtracted from each *rut* site and all the resulting values for each gene were summarized to give a *rut* probability per gene (Supplementary Material M2 and M3).

### RNA isolation

*Cupriavidus metallidurans* strains CH34 and AE104 were cultivated to a turbidity of 120 Klett units. EDTA was added at 50 µM for both strains, and 3.35 mM MultiTox metal mix specific for CH34, 1 mM for AE104, or no addition was made. After a further 10 min incubation with shaking at 30°C, cells were rapidly harvested at RT and stored at −80°C. Total RNA was isolated with RNeasy Plus Mini Kit (Qiagen, Hilden, Germany) according to the manufacturer's instruction. One DNase treatment was performed. To exclude experimental artifacts resulting from DNA contamination, only RNA was used that did not generate products in several PCR reactions with chromosomal- and plasmid-designed oligonucleotide primers. RNA concentration was determined photometrically, and RNA quality was checked on formamide gels [66] and measured as RNA integrity number on an Agilent 2100 Bioanalyzer (Agilent Technologies, Waldbronn, Germany). Three biological repeats were done per strain and condition.

### Determination of the transcriptome

RNASeq was performed by Vertis Biotechnology AG (Freising, Germany). The program TraV (Mac) [28] was used to calculate the NPKM values for all genes, 5′ and 3′ untranslated regions (5UTR, 3UTR), free and antisense transcripts (FT, AST) for all strains (CH34, AE104), conditions (no addition, EDTA, metal mixes), and biological repeats. Gene bank accession CH34.gbk served as the basis for annotation and contains the replicons CP000352, 3, 4, and 5 corresponding to the chromosome, chromid, plasmid pMOL30, and pMOL28, respectively. The NPKM values were the transcript abundances of annotated features such as genes for proteins and RNAs. The 5UTR and 3UTR indicated continued transcription directly adjacent to the annotated features. The constraint for FT were transcripts outside of annotated features neither on the positive nor negative DNA strand. AST was finally similar to FT but required an annotated feature on the other DNA strand [28]. ASTs were thus uninterrupted transcript abundances that continued into, or across, regions that contained annotated features on the other DNA strand.

The 10 179 sense transcripts (genes, 3UTRs, 5UTRs) in untreated cells of *C. metallidurans* CH34 were listed according to the associated Rmet locus tag with the mean NPKM value and deviation of the three biological repeats, associated antisense transcript, mean NPKM value plus deviation of the AST signal and the ratio of the mean NPKM values of the sense and antisense transcript “S/AS”. These contained the values for ORFs, 3UTRs, and 5UTRs. Moreover, the sense signals were associated with operons as described [55], the *rut* score, the KEGG orthology [67, 68], and description of the gene product (Supplementary Material M2). In five comparisons, the ratios of the sense, antisense, and S/AS values were calculated. These were the comparisons metal-challenged to non-challenged CH34 cells (CH34_M_0), EDTA-challenged to non-challenged CH34 cells (CH34_E_0), comparison of non-challenged AE104 to CH34 cells (0_AE104_CH34), and the same for metal- (M_AE104_CH34) and EDTA-(E_AE104_CH34) challenged cells (Supplementary Material M2). All significant differences (*D* > 1) with ratios below 0.5 or above 2 were noted. This comparison allowed to identify the sense transcripts regulated under metal stress or starvation, in strain AE104 differently compared to the wild- type CH34, compare this regulation with that of the associated antisense transcript and assign everything to a metabolic pathway via the KEGG orthology system.

The signals for the genes were named according to the Rmet locus tag of the respective gene, for instance “NPKM_6315” as signal for locus tag Rmet_6315, which is the *parA* gene of plasmid pMOL28. The 5′ and 3′ untranslated regions were named according to their directly adjacent downstream gene as “5UTR” and “3UTR” signals, respectively. The free and antisense transcripts were “FT” or “AST”, followed by the position of the first base, “−” or “+” for the DNA strand and the number 2, 3, 4, or 5 that identified the replicon, for instance “AST_39 425 + 5” starts at position 39 425 on the “+”-strand of replicon CP000355 or plasmid pMOL28. Following the naming, the mean values and deviations of the NPKM values of the three biological repeats were calculated for all data points, genes, UTRs, FTs, and ASTs. Since the annotation of the CH34 genome has changed many times in the last years, the “Rmet” locus tags were used to allow comparability with older data.

To identify overlaps between the ASTs and the sense transcripts from genes and UTRs, the ASTs, 5UTRs, 3 UTRs, and annotated ORFs were sorted in the order of the increasing start (DNA “+” strand) or decreasing (DNA “−” strand) stop position. In this way, “Cont”-ASTs were identified that directly continued a sense transcript of the ORF of a gene (distance up to 15 bp from the position of the last bp of the stop codon allowed but was 1 is most cases). All other ASTs were annotated as “FREE”. These started within a gene or UTR and this overlap was noted, or they started outside of an annotated region of a sense transcript. After bridging regions without regions of annotated sense transcripts, some ASTs continued into other annotated regions of sense transcripts further downstream.

To assign ASTs as an antisense transcript specifically to a sense transcript, the ASTs were also listed in the order of the start and stop position of the annotated sense transcripts on the other DNA strand. An AST was assigned to a gene or UTR, if it stopped, started or ran across a sense region. In this way, for each gene or UTR: (i) A mean value and deviation of the sense NPKM values was obtained; (ii) If present, an associated AST with its FREE, Cont, or overlapping feature with respect to the transcripts on the same DNA strand could be identified; (iii) The NPKM mean value and deviation of the AST were defined; and (iv) The ratio of the sense and antisense NPKM values S_AS was determined. These results were obtained for both strains and their three different growth conditions, CH34_0 without metal shock, CH34_M shocked with its strain-specific metal mix, CH34_E treated with EDTA, and again the analogous conditions for strain AE104, AE104_0, AE104_M, AE104_E.

In the final comparison, the sense and antisense NPKM values for two strains or conditions were compared. The *Q* ratios were calculated for the sense, antisense NPKM values and the S_AS values. The *D* values (absolute difference of the mean values divided by the sum of the deviations) were also calculated. A value was considered as significant if ((*Q* > 2 or *Q* ≤ 0.5) and *D* > 1). The comparisons were CH34_M_0 (metal shock divided by non-shocked cells), CH34_E_0 (EDTA or not), 0_AE104_CH34 (difference of the strains under standard growth conditions), M_AE104_CH34 (cells shocked with their specific metal mix), and E_AE104_CH34 (comparison of the EDTA-treated cells). Finally, all gene and UTR loci with their respective “Rmet” locus tags were assigned to their predicted operons, the KEGG orthology and a description of the gene products.

### RT-PCR

For the RT reaction, 1 µg of total RNA and 0.1 µg hexamer or gene-specific primers were incubated at 65°C for 5 min and snap-cooled on ice. After addition of 0.5 mM each of dATP, dGTP, dTTP and dCTP, 20 mM DTT, and 100 U of reverse transcriptase (superscript II) in reaction buffer (Thermo Fisher Scientific, Germany) reverse transcription proceeded for 10 min at room temperature, followed by 1 h at 42°C. After finishing the RT reaction, the enzyme was inactivated at 70°C for 10 min. One microliter of the resulting cDNA was amplified by PCR with 0.2 µM of each primer and 1 U of Taq-Polymerase (Roche Diagnostics GmbH, Mannheim, Germany). A no-template control and a no-RT (negative) control were performed under identical conditions as for the target genes. The primer sequences are in Table S6.

### Statistics

Students’ *t*-test was used but in most cases the distance (D) value, *D*, has been used several times previously for such analyses [69–71]. It is a simple, more useful value than Student's *t*-test because non-intersecting deviation bars of two values (*D* > 1) for three repeats always means a statistically relevant (≥95%) difference provided the deviations are within a similar range. At *n* = 4, significance is ≥97.5%, at *n* = 5 ≥ 99% (significant) and at *n* = 8 ≥ 99.9% (highly significant).

## Supplementary Material

mfae057_Supplementary_Files

## Data Availability

RNASeq data were deposited as BioProject PRJNA753702.
