# Supplementary material for: Antisense transcription is associated with expression of metal resistance determinants in Cupriavidus metallidurans CH34
Source: Metallomics. 2024 Nov 19;16(12):mfae057. doi: 10.1093/mtomcs/mfae057 (PMC11647585; doi:10.1093/mtomcs/mfae057)
Supplement: mfae057_Supplementary_Files [file mfae057_supplementary_files.zip › Suppl_data_Supplement.pdf]

Supplement

Antisense transcriptome in *Cupriavidus metallidurans*

Antisense activities. Using the RNASeq data, the program TraV Mac (1) was used to calculate the transcript abundances as NPKM values (nucleotide activities per kilobase of exon model per million mapped reads) for all genes, 5' and 3' untranslated regions (5UTR, 3UTR), free and antisense transcripts (FT, AST) for all strains (CH34, AE104), conditions (no addition, EDTA, MultiTox metal mixes) and biological repeats. The signals for the genes were named according to the Rmet locus tag of the respective gene, for instance "NPKM_6315" as signal for locus tag Rmet_6315, which is the *parA* gene of plasmid pMOL28. The 5' and 3' untranslated regions were named according to their directly adjacent downstream gene as "5UTR" and "3UTR" signals, respectively. The free and antisense transcripts were "FT" or "AST", followed by the position of the first base, "-" or "+" for the DNA strand and the number 2, 3, 4, or 5 that identified the replicon, for instance "AST_39425+5" starts at position 39,425 on the "+"-strand of replicon CP000355 or plasmid pMOL28. Following the naming, the mean values and deviations of the NPKM values of the three biological repeats were calculated for all data points, genes, UTRs, FTs and ASTs.

It is important to note that ASTs were uninterrupted transcript abundances that continued into or across regions with open reading frames on the other DNA strand (1). The software recognized regions with high transcript abundances between those with low or no abundance. Should such a region contain a segment in antisense orientation to open reading frames on the other DNA strand, they were defined as "AST". This did not exclude that another portion of this region contains sense transcripts for genes, 3UTRs or 5UTRs. The NPKM value was subsequently calculated for the complete AST, including sense and antisense parts. Consequently, an annotated AST could either represent a discrete RNA molecule or a group of overlaying RNA molecules in a transcribed region. Some RNAs in a bin may be mRNAs, others asRNAs or even small RNAs but at least one RNA in the bin should have a portion with an antisense feature. An AST as defined here could be bin and should not be automatically considered as a molecule.

The number of 6,841 annotated features (Suppl. Tab. S1) with NPKM values corresponded mostly to the 6,771 genes (locus tags Rmet) plus 96 RNAs such as tRNAs (locus tag Rmet_R), with 26 no longer existing annotated features missing. The mean size of the genes of 923 ± 624 bp corresponded to the expected mean size of a bacterial gene of 1 kb (2). RNAs that were transcripts of annotated features filled between 82% (pMOL30) and 91% (chromosome) of the coding capacity of the replicons (referred to the total number of base pairs, which is the coding capacity of one DNA strand), with plasmid pMOL30 being especially loosely packed.

Supplementary Table S1. Predicted RNAs in non-challenged cells of *C.* metallidurans CH34[a]

Replicon	Chromosome	Chromid	pMOL30	pMOL28
total bp	3,928,089	2,580,084	237,720	171,459
NPKM, n	3,876	2,507	283	175
NPKM, MEAN bp	923±624	929±679	693±591	873±683
NPKM, total bp	3,577,519	2,32,8331	196,020	152,847
%total bp	91.1%	90.2%	82.5%	89.1%
5UTR, n	789	771	74	36
5UTR, MEAN bp	467±788	227±335	394±432	493±822
5UTR, total bp	368,394	174,755	29,146	17,760
%total bp	9.4%	6.8%	12.3%	10.4%
3UTR, n	789	788	78	36
3UTR, MEAN bp	997±1095	564±588	813±910	877±1144
3UTR, total bp	786,372	444,264	63,444	31,587
%total bp	20.0%	17.2%	26.7%	18.4%
AST, n	5016	5300	231	210
AST, MEAN bp	1,262±3,693	661±1,806	1,558±2,880	1,104±2,449
AST, total bp	6,330,941	3,50,2951	359,909	231,919
%total bp	161%	136%	151%	135%
FT, n	112	233	28	12
FT, MEAN bp	114±70	116±81	137±92	105±50
FT, total bp	12,746	27,132	3,838	1,264
%total bp	0.3%	1.1%	1.6%	0.7%
All, bp	11,075,972	6,477,433	652,357	435,377
%total bp	282%	251%	274%	254%
All-AST, bp	4,745,031	2,974,482	292,448	203,458
%total bp	121%	115%	123%	119%

[a]The number of base pairs of the four replicons is indicated in the first line. RNAs from genes (NPKM), 5' and 3' untranslated regions (5UTR. 3UTR), antisense (AST) and free transcripts (FT) are listed with the total number of these RNAs n, the mean value of the length with deviations, the sum of these lengths and the ratio of this sum divided by the total number of base pairs in the replicon as listed in the first line (coding capacity of one DNA strand). All gives the sum of all these RNAs, All-AST of all except the sum of the lengths of the ASTs.

Only 1,670 5UTRs were found (Suppl. Tab. S1) and their mean size per replicon was between 200 and 500 bp, filling 7% to 12% of the coding capacity of the four replicons. Not all genes possessed an upstream 5UTR. The total number of 1,670 5UTRs corresponded more closely to the 2119 operon regions (3) in the *C. metallidurans* genome, so that about 80% of the operon regions may possess a 5UTR. The number of 3UTRs was very similar to that of the 5UTRs, 1,691, again indicating one 3UTR per operon region, but the 3UTRs were much longer than the 5UTRs, between 500 bp and 1 kb, filling about 20% of the coding capacity of the replicons (Suppl. Tab. S1).

A large number of 10,757 regions of antisense activities were found with mean sizes between 600 bp and 1.6 kb, depending on the replicon (Suppl. Tab. S1, Suppl. Mat. M1). The ASTs filled the 1.5-fold coding capacity of the replicons, meaning that antisense transcripts overlapped with transcripts of genes, 5UTRs and 3UTRs. When the lengths of all annotated transcript activities were summarized, a 2.5- to 2.8-fold coverage of the replicons was found, which agrees with ASTs overlapping with all other elements. In contrast, only 385 free transcripts activities were found, which covered only about 1% of the total coding capacity. The mean size of a FT was about 100 bp (Suppl. Tab. S1). The total lengths of all annotated sense transcripts was 120% of the coding capacity. 5UTRs and 3UTRs were annotated as regions with similar transcript abundance upstream and downstream, respectively, of a gene on the same DNA strand, not considering an overlap with a gene on the other DNA strand (1). This explains why genes, 5UTRs and 3UTRs filled 120% of the total coding capacity genome. ASTs, annotated as regions of similar transcript abundances opposite to annotated genes, overlapped with 5UTRs, 3UTRs and continued into or across sense transcripts.

When all ASTs were selected with a constraint NPKM > 10 and a length > 50 bp, only 1,319 AST of the 10,757 ASTs remained (Suppl. Tab. S2). These could be counted as expressed and sufficiently long ASTs to have a biological effect. Most were abundant on a low level with NPKM ≤ 100 and only three on a very high level with NPKM > 1000. These were AST_793320-2, AST_3009456+2 and AST_3037043+2 with sizes between 7 kb and 9 kb. Also, three ASTs remained with sizes above 50 kb, AST_2324569-2, AST_3403828-2 and AST_3832310-2, which were abundant with NPKM values between 220 and 340 (Suppl. Tab. S3). In contrast to the ASTs, only 12 FTs remained when a constraint of NPKM > 10 was used (Suppl. Tab. S2), all of them had sizes above 50 bp. One of these FTs, FT_2348650+3, was present on a low level of abundance, a second one (FT_727889-3) was present in high numbers with an NPKM value of 645, the remaining 10 FTs on a very low level with NPKM < 30.

Supplementary Table S2. Distribution of the NPKM values and length of antisense and free transcript activities in non-challenged cells of *C. metallidurans* CH34[a]

Replicon	Chromosome	Chromid	pMOL30	pMOL28	Total
AST (antisense transcripts)					
NPKM >10, length > 50 bp	710	518	53	38	1319
Length > 50 bp and:					
-NPKM >30, NPKM ≤ 100	243	182	21	9	455
-NPKM >100, NPKM ≤ 300	157	28	1	6	192
-NPKM >300, NPKM ≤ 1000	47	8	0	1	56
-NPKM > 1000	3	0	0	0	3
NPKM >10 and:					
-Length > 0.5 kb and length ≤ 1.5 kb	66	65	8	9	148
-Length > 1.5 kb and length ≤ 5 kb	283	275	20	13	591
-Length > 5 kb and length ≤ 15 kb	252	135	22	12	421
-Length > 15 kb and length ≤ 50 kb	70	10	2	1	83
-Length > 50 kb	3	0	0	0	3
FT (free transcripts)					
NPKM >10, length > 50 bp	4	7	1	0	12
Length > 50 bp and					
-NPKM >30, NPKM ≤ 100	0	1	0	0	1
-NPKM >100, NPKM ≤ 300	0	0	0	0	0
-NPKM >300, NPKM ≤ 1000	0	1	0	0	1

The Table lists the antisense and free transcripts above a cut-off value of a NPKM > 10 and a length > 50 bp. For the ASTs, these were further sub-distributed according to their NPKM values or sizes. All free transcripts with NPKM > 10 were larger than 50 bp

With the algorithm used, not many free transcript activities were found in non-challenged CH34 cells. In contrast, antisense transcription activities were found that covered the complete sense transcripts of genes, 5UTRs and 3 UTRs. The majority of these antisense activities were of a small size or low abundance. Nevertheless, antisense transcription activities clearly existed in *C. metallidurans* cells.

<u>Localization of ASTs with respect to sense transcripts on the same DNA strand</u>. The ASTs, 5UTRs, 3 UTRs and annotated open reading frames (ORFs) were sorted in the order of the increasing start (DNA “+” strand) or decreasing (DNA “-“ strand) stop position. Among the 10,757 ASTs, only 132 were directly continuing sense transcripts (Suppl. Tab. S4, Suppl. Mat. M1). About 2/3 of these continuing “Cont-ASTs” had a NPKM value > 10. Except for 7 of the 132 Cont-ASTs, the distance to the upstream sense feature was 1, meaning that a sense transcript continued directly into a downstream transcript that acted as antisense transcripts for sense transcripts from the other DNA strand. They may represent discrete RNA molecules composed of an mRNA at the 5’-end and an asRNA part at the 3’-end. Among the 7 exceptions,

only 1 was expressed with NPKM > 10. Only 17 of the 132 Cont-ASTs did not continue into a subsequent downstream sense feature, 115 did and of these, 84 were expressed with NPKM > 10. This means that these 84 RNAs were “double-use” transcripts, which are sense transcripts at the 5’ beginning and 3’ end, and contained at least one middle part that acted as antisense transcript for transcripts from the other DNA strand. These “double-use” transcripts may represent discrete RNA molecules or a bin of transcripts from different parts of the annotated AST region.

Another large part of the expressed (NPKM > 10) ASTs also continued as sense transcript on one DNA strand after acting as antisense transcripts for the other DNA strand (FREE and continuing, Suppl. Tab. S4). About half of them started outside of annotated features on the same DNA strand. These may represent “double-use” transcripts with a 5’ antisense and a 3’ sense part. Another half overlapped with 5UTRs and 101 ASTs overlapped with ORFs but started upstream of the stop codon. These were also “double-use” transcripts, however, in contrast to the 84 Cont-ASTs, the part of the 5’ end may act as sense mRNA and as antisense transcript for a sense mRNA from the other DNA strand. Together with the 84 Cont-ASTs, the “double-use” ASTs comprised the majority of 82% of the expressed ASTs. These ASTs might couple transcription events on both DNA strands.

The vast majority of the FREE-ASTs did not continue into sense transcripts but only 2% of these FREE-ASTs were expressed with NPKM > 10. If expression of these ASTs was never up-regulated, they may represent noise of the RNASeq method. Very few of the non-continuing FREE-ASTs started within an ORF, 3UTR, or 5UTR on the same DNA strand, and 23 of these were expressed with NPKM > 10. In 214 instances, FREE-ASTs were not overlapping with or extending into a sense transcriptional region and were also expressed. These 214 FREE-AST (16%) comprise a second group of ASTs that was independent from sense transcripts and could be ASTs without parts that may serve as sense RNAs.

The three highly expressed (NPKM > 1000, Suppl. Table S3) ASTs AST_793320-2, AST_3009456+2 and AST_3037043+2 with sizes between 7 kb and 9 kb all crossed into open reading frames, the small ORF Rmet_6426, *ompA* and genes adjacent to those involved in cobalamin biosynthesis and uptake, respectively. These ASTs probably represented a group of overlapping transcripts. The same was true for the three long (> 50 kb) ASTs AST_2324569-2, AST_3403828-2 and AST_3832310-2. They continued into sense transcripts for DNA replication genes, cell division and murein biosynthesis and *ompW*, respectively. AST_2324569-2 was an antisense transcript for the *rho* gene for the transcription termination factor but also sense transcript for the *rpoS* gene for the starvation sigma factor. This arrangement may couple expression of the gene for the starvation sigma factor with that for the terminator.

AST_3832310-2 was also a sense transcript for the *atp* operon for the F_1F_0 ATPase, *cupA* and *cupR* for the main copper-exporting P-type ATPase and its MerR-type regulator, before being antisense again for *cupC* for the copper chaperone (Suppl. Mat. M2, Suppl. Tab. S3). These transcripts were “double-use” transcripts forming a mosaic of sense and antisense parts and most probably a bin of transcripts.

The “double-use” transcripts should display a similar abundance for the sense and antisense parts but this could not be observed (Suppl. Mat. M2). Transcription events starting at different transcriptional start sites may run across annotated sense or antisense regions so that the measured NPKM values of these ASTs are the sum of the abundances of overlaying transcripts. Knowledge of the transcriptional start sites and their regulation would be required to understand such a process.

Abundant antisense transcripts seem to exist in *C. metallidurans* cells, most of them (82%) are dual-use transcripts that are part sense and part antisense. A second group (16%) of expressed ASTs were independent from sense transcripts. The 82% ASTs might connect transcription events on both DNA strands, the 16% act independently. A discrete RNA molecule or a bin of overlapping transcripts could be behind these annotated ASTs.

Supplementary Table S3. The three most abundant and the three longest antisense transcripts in non-challenged CH34 cells[a].

Element	locus tag	gene	MEAN	SDEV	S/AS	Operon	Description
**Antisense transcripts with the highest NPKM values (all on the bacterial chromosome)**							
AST_793320-2, NPKM = 3,909±403							
NPKM_0709	Rmet_0709		116.0	12.1	0.03	Op0201f_1	Q1LQI1 Putative uncharacterized protein
NPKM_6425	Rmet_6425		284.3	96.0	0.07	Op0201f_1	NA
3UTR_6425	Rmet_6425		28.7	5.5	0.01	Op0201f_1	NA
5UTR_0713	Rmet_0713	*gyrA*	99.3	11.1	0.03	Op0203f_1	Q1LQH7 DNA gyrase subunit A
NPKM_0713	Rmet_0713	*gyrA*	368.0	3.0	0.09	Op0203f_1	Q1LQH7 DNA gyrase subunit A
NPKM_0714	Rmet_0714		750.3	32.9	0.19	Op0203f_2	Q1LQH6 Putative signal peptide protein
NPKM_0715	Rmet_0715	*serC*	293.7	8.1	0.08	Op0203f_2	Q1LQH5 Phosphoserine aminotransferase
NPKM_0716	Rmet_0716	*pheA*	249.3	3.8	0.06	Op0203f_2	Q1LQH4 Prephenate dehydratase
AST_3009456+2, NPKM = 1,017±228							
NPKM_2767	Rmet_2767	*mrp*	116.7	7.2	0.11	Op0776r_1	Q1LJN6 Cobyrinic acid a,c-diamide synthase
NPKM_2770	Rmet_2770		68.0	7.8	0.07	Op0778r_2	Q1LJN3 Putative lipoprotein
NPKM_2771	Rmet_2771		55.0	3.0	0.05	Op0778r_1	Q1LJN2 Putative uncharacterized protein
AST_3037043+2, NPKM = 2,794±546							
NPKM_2789	Rmet_2789	*btuB*	93.0	2.0	0.03	Op0784r_1	Q1LJL4 TonB-dependent receptor, plug
NPKM_2794	Rmet_2794	*matB*	51.3	4.2	0.02	Op0786r_3	Q1LJK9 AMP-dependent synthetase and ligase
3UTR_2794	Rmet_2794	*matB*	37.0	4.4	0.01	Op0786r_3	Q1LJK9 AMP-dependent synthetase and ligase
NPKM_2795	Rmet_2795		27.0	2.0	0.01	Op0786r_3	Q1LJK8 Enoyl-CoA hydratase/isomerase
**Longest antisense transcripts (all on the bacterial chromosome)**							
AST_2324569-2, NPKM =230±26							
NPKM_2087	Rmet_2087	*narL*	10.3	2.5	0.04	Op0603f_1	Q1LLL0 Transcriptional regulator, LuxR family
3UTR_2089	Rmet_2089	*talB*	34.0	4.0	0.15	Op0605f_1	Q1LLK8 Transaldolase
NPKM_2089	Rmet_2089	*talB*	140.7	3.2	0.61	Op0605f_1	Q1LLK8 Transaldolase
5UTRR0025	Rmet_R0025		109.7	9.9	0.48	Op0607f_1	NA
NPKMR0025	Rmet_R0025		11978.3	4371.6	52.00	Op0607f_1	NA
NPKM_2093	Rmet_2093		359.3	27.7	1.56	Op0607f_2	Q1LLK4 Transcriptional regulator, BadM/Rrf2 family
NPKM_2094	Rmet_2094		43.3	2.9	0.19	Op0607f_2	Q1LLK3 NAD-dependent epimerase/dehydratase
3UTR_2094	Rmet_2094		14.3	1.5	0.06	Op0607f_2	Q1LLK3 NAD-dependent epimerase/dehydratase

5UTR_2111	Rmet_2111		138.7	15.2	0.60	Op0609f_1	Q1LLI6 Putative uncharacterized protein
NPKM_2111	Rmet_2111		1128.7	86.6	4.90	Op0609f_1	Q1LLI6 Putative uncharacterized protein
3UTR_2111	Rmet_2111		35.7	4.0	0.15	Op0609f_1	Q1LLI6 Putative uncharacterized protein
5UTR_2113	Rmet_2113		27.0	3.5	0.12	Op0611f_1	Q1LLI4 Endonuclease/exonuclease/phosphatase
NPKM_2113	Rmet_2113		30.3	1.2	0.13	Op0611f_1	Q1LLI4 Endonuclease/exonuclease/phosphatase
3UTR_2113	Rmet_2113		6.3	1.2	0.03	Op0611f_1	Q1LLI4 Endonuclease/exonuclease/phosphatase
5UTR_2119	Rmet_2119		20.3	2.1	0.09	Op0613f_1	Q1LLH8 Alcohol dehydrogenase, zinc-binding
NPKM_2119	Rmet_2119		36.3	1.5	0.16	Op0613f_1	Q1LLH8 Alcohol dehydrogenase, zinc-binding
3UTR_2119	Rmet_2119		9.3	1.2	0.04	Op0613f_1	Q1LLH8 Alcohol dehydrogenase, zinc-binding
5UTRR0026	Rmet_R0026	*ffs*	104.0	38.9	0.45	Op2026f_1	Signal recognition particle 4.5S RNA
NPKMR0026	Rmet_R0026	*ffs*	28519.0	15060.0	123.82	Op2026f_1	Signal recognition particle 4.5S RNA
3UTRR0026	Rmet_R0026	*ffs*	124.0	13.5	0.54	Op2026f_1	Signal recognition particle 4.5S RNA
5UTR_2134	Rmet_2134	*trxA*	216.0	20.9	0.94	Op0615f_1	Q1LLG3 Thioredoxin
NPKM_2134	Rmet_2134	*trxA*	1352.0	27.9	5.87	Op0615f_1	Q1LLG3 Thioredoxin
NPKM_2135	Rmet_2135	*rho*	509.0	40.0	2.21	Op0615f_1	Q1LLG2 Transcription termination factor Rho
AST_3403828-2, NPKM = 220±24							
NPKM_3095	Rmet_3095	*hpt*	207.7	5.0	0.94	Op0871f_1	Q1LIQ9 Phosphoribosyltransferase
3UTR_3095	Rmet_3095	*hpt*	126.3	14.3	0.57	Op0871f_1	Q1LIQ9 Phosphoribosyltransferase
5UTR_3101	Rmet_3101	*nudH*	30.0	4.6	0.14	Op0873f_1	Q1LIQ3 NUDIX hydrolase
NPKM_3101	Rmet_3101	*nudH*	642.3	30.5	2.92	Op0873f_1	Q1LIQ3 NUDIX hydrolase
NPKM_3102	Rmet_3102		486.7	25.6	2.21	Op0873f_1	Q1LIQ2 Putative lipoprotein
NPKM_3107	Rmet_3107	*ispB*	581.0	56.3	2.64	Op0875f_1	Q1LIP7 Polyprenyl synthetase
NPKMR0049	Rmet_R0049		3110.7	1065.1	14.12	Op0875f_1	NA
NPKM_3108	Rmet_3108	*pilB*	292.7	5.7	1.33	Op0875f_2	Q1LIP6 Type II secretion system protein E
NPKM_3109	Rmet_3109	*pilC*	296.3	17.0	1.34	Op0875f_2	Q1LIP5 Type II secretion system protein
NPKM_3110	Rmet_3110	*pilD*	291.0	2.0	1.32	Op0875f_2	Q1LIP4 Type 4 prepilin peptidase 1. Aspartic peptidase. MEROPS family A24A
NPKM_3111	Rmet_3111	*coaE*	125.3	5.1	0.57	Op0875f_2	Q1LIP3 Dephospho-CoA kinase
NPKM_3112	Rmet_3112		631.0	15.6	2.86	Op0875f_3	Q1LIP2 UPF0289 protein Rmet_3112
NPKM_3113	Rmet_3113		570.0	24.9	2.59	Op0875f_3	Q1LIP1 Putative uncharacterized protein
3UTR_3113	Rmet_3113		18.7	2.1	0.08	Op0875f_3	Q1LIP1 Putative uncharacterized protein
5UTR_6576	Rmet_6576		7.0	0.0	0.03	Op2047f_1	NA

NPKM_6576	Rmet_6576		5.7	0.6	0.03	Op2047f_1	NA
3UTR_6576	Rmet_6576		0.3	0.6	0.00	Op2047f_1	NA
5UTR_3120	Rmet_3120		12.0	1.0	0.05	Op0877f_1	Q1LIN4 Putative uncharacterized protein
NPKM_3120	Rmet_3120		120.3	8.3	0.55	Op0877f_1	Q1LIN4 Putative uncharacterized protein
3UTR_3120	Rmet_3120		19.7	2.5	0.09	Op0877f_1	Q1LIN4 Putative uncharacterized protein
5UTR_3139	Rmet_3139		411.7	39.7	1.87	Op0879f_1	Q1LIL5 Putative uncharacterized protein
NPKM_3139	Rmet_3139		447.0	44.6	2.03	Op0879f_1	Q1LIL5 Putative uncharacterized protein
AST_3832310-2, NPKM = 334±45 (also mRNA for the *atp* operon as well as *cupA* and *cupR* upstream of *cupC)*							
NPKM_3483	Rmet_3483	*sdaA*	21.7	2.3	0.06	Op0969f_2	Q1LHM1 L-serine ammonia-lyase
NPKM_3484	Rmet_3484		39.0	5.6	0.12	Op0969f_2	Q1LHM0 Beta-lactamase-like protein
3UTR_3484	Rmet_3484		13.3	1.5	0.04	Op0969f_2	Q1LHM0 Beta-lactamase-like protein
5UTR_3502	Rmet_3502		115.3	17.8	0.35	Op0971f_1	Q1LHK2 Putative uncharacterized protein
NPKM_3502	Rmet_3502		36.0	4.4	0.11	Op0971f_1	Q1LHK2 Putative uncharacterized protein
3UTR_3502	Rmet_3502		10.3	1.5	0.03	Op0971f_1	Q1LHK2 Putative uncharacterized protein
5UTR_3509	Rmet_3509		157.7	24.8	0.47	Op0973f_1	Q1LHJ5 Transcriptional regulator, LysR family
NPKM_3509	Rmet_3509		77.7	2.1	0.23	Op0973f_1	Q1LHJ5 Transcriptional regulator, LysR family
3UTR_3509	Rmet_3509		25.3	2.3	0.08	Op0973f_1	Q1LHJ5 Transcriptional regulator, LysR family
5UTR_6600	Rmet_6600		5.7	0.6	0.02	Op2057f_1	NA
NPKM_6600	Rmet_6600		5.0	0.0	0.01	Op2057f_1	NA
3UTR_6600	Rmet_6600		7.3	2.1	0.02	Op2057f_1	NA
5UTR_3525	Rmet_3525	*cupC*	0.7	0.6	0.00	Op0975f_1	Q1LHH9 Heavy metal detoxification protein
NPKM_3525	Rmet_3525	*cupC*	184.3	37.2	0.55	Op0975f_1	Q1LHH9 Heavy metal detoxification protein
3UTR_3525	Rmet_3525	*cupC*	11.7	1.2	0.03	Op0975f_1	Q1LHH9 Heavy metal detoxification protein
5UTR_3533	Rmet_3533	*phhA*	23.3	5.5	0.07	Op0977f_1	Q1LHH1 Phenylalanine 4-hydroxylase
NPKM_3533	Rmet_3533	*phhA*	24.0	4.6	0.07	Op0977f_1	Q1LHH1 Phenylalanine 4-hydroxylase
NPKM_3534	Rmet_3534	*phhB*	85.7	5.9	0.26	Op0977f_2	NA

[a]The three most abundant and the three longest antisense transcripts are listed in association with their sense transcripts annotated as gene (NPKM), 5UTR or 3UTR. The Rmet locus tag, gene name, NPKM and deviation of the sense transcript, the ratio sense/antisense NPKM, the operon of the sense transcript and the description of the gene follows from the left to the right. For AST_3832310-2, the two lines label the position of the *atp* operon and the *cupAR* genes, respectively.

Supplementary Table S4. Assignment of antisense activities to annotated features on the same DNA strand in non-challenged cells of *C. metallidurans* CH34[a]

AST	Number	Number > NPKM = 10	% NPKM > 10
ASTs assigned total	10,757	1319	12.3%
FREE ASTs	9,378	237	2.5%
•• not overlapping 5’	9,195	214	2.3%
•• overlap with 3UTR	79	7	8.9%
•• overlap with 5UTR	46	7	15.2%
•• overlap with ORFs	58	9	15.5%
FREE and continuing	1,247	997	80.0%
•• not overlapping 5’	534	436	81.6%
•• overlap with 3UTRs	14	9	64.3%
•• overlap with 5UTRs	517	451	87.2%
•• overlap with ORFs	182	101	55.5%
Continuing ASTs	132	85	64.4%
•• into features	115	84	73.0%

On the 8 DNA strands of the 4 replicons, the genes, 3UTR, 5UTR and AST signals were sorted in the order of the increasing start (+ strands) or decreasing stop positions (- strands). This information was used to compare the ASTs with the other annotated signals on the same DNA strand. ASTs annotated with a “FREE” started at least 15 bp downstream of the end of upstream open reading frames (ORFs) but sometimes in the subsequent 5UTR, 3UTR or ORF. A few ASTs were between 1 and 14 bp upstream of an 5UTR, 3UTR or ORF (< 15 bp at 3' end). Some FREE-ATRs are also continuing into respective downstream ORF, 3UTR or 5UTR. ASTs directly downstream (maximum 14 bp distance) of ORFs were annotated as “Cont”, continuing ASTs. All ASTs are listed in the supplementary AST-Assignment file.

Association of antisense transcripts to their sense transcripts from the other DNA strand. For each replicon, DNA strand, strain and growth condition, the antisense transcripts were associated with sense transcripts (genes, 5UTRs, 3UTRs) on the other DNA strand, if they started, stopped or continued across the region of an annotated sense transcript (Suppl. Mat. M2). After association of antisense, sense transcripts and their respective NPKM values, the ratio of the NPKM values of the sense transcript divided by the antisense transcript was calculated.

The long sizes of some antisense transcripts may be due to regulation of the genes in CMGIs (catabolic metabolic chromosomal islands, (4, 5)). To investigate this, the natural logarithms of the NPKMs of the sense and associated antisense transcripts were plotted against the Rmet locus numbers in chromosomal regions that contain CMGIs. For the “-“ strand, these values were multiplied by -1 (Suppl. Fig. S1). There appeared to be a very low content of antisense transcripts of the “-“ strand within CMGI1, CMGI2 and CMGI3 but not for CMGI4, so that a low content of antisense transcripts for the “-“ strand of CMGIs was not the

rule. On the other hand, antisense transcripts for all genes on the "+" strand were visible. Sometimes, the abundance of the antisense transcripts was higher than that of the sense transcripts, while sometimes it was the other way around. Nevertheless, if the longest and most abundant antisense transcripts were not associated to CMGIs then this hypothesis was falsified.

Factor Rho and *rut*-sites. The "open-donut"-shaped ATP-dependent Rho factor mediates transcription termination. AST_2324569-2 may couple the expression of the *rho* gene with that of *rpoS,* which encodes the starvation sigma factor. Rho competes with the ribosome for the mRNA, which increases the quality of the transcription-translation coupling (6-8), and which would be especially required under starvation conditions. An antisense RNA should be a fourth molecule competing for an mRNA, in addition to RNAses, Rho and the ribosomes. It was analyzed whether *rut* (Rho utilization site) sites were enriched in regions with high antisense activity, or if the opposite was the case.

The program RhoTermPredict (9) was used to predict *rut* sites and possible Rho-dependent transcription terminators in the genome of *C. metallidurans* CH34. A number of 1,476 possible *rut* sites were found, which contained up to 30 scores for individual sequences of the respective 78 bp fragment. The scores were between 6 and 15, depending on the C/G ratio, existence of inverted sequence features and possible pause sites downstream of the *rut* sequence (9). For each site, the maximum score was calculated (RTS_ALL). These *rut* sites were associated with an annotated gene if the 5' end of the *rut* site was downstream of the 5' end of the open reading frame on the same DNA strand. For each *rut* site, 5 was subtracted from its score so that the weakest *rut* site had a resulting score of 1, the strongest of 10. For each gene, the scores of all its associated *rut* sites were summarized (Suppl. Mat. M3).

The strongest *rut* score was 801 for Rmet_0965 encoding a transcriptional regulator, followed by 607 for *nodJ* (gene for a ABC transporter component) and Rmet_3704 (encoding a porin), 547 for Rmet_3672 (putative uncharacterized gene product) and 510 for Rmet_4693 (gene for a lytic transglycosylase). These genes had associated ASTs that were all continuing sense transcripts. Rmet_0965 and Rmet_3672 were strongly expressed with NPKM-values above 1,000, but with much lower AST abundances. In comparison, the AST associated with *nodJ* was abundant with a NPKM value of 415 ± 45 while the respective value for the sense transcript was only 47.7 ± 5.9. The other two genes were not expressed under the tested conditions. No insight into a possible interaction of Rho and ASTs resulted from these data for the genes with the strongest possibility of Rho-dependent termination events. For all genes, the *rut* scores were plotted against the abundances of their ASTs (Suppl. Fig. S2A, open grey circles). No connection between the *rut* score of a gene and the antisense abundance was evident at this stage.

Supplementary Figure S1. Sense and antisense transcripts in the chromosomal region of the four longest CMGIs. The plot gives the ln of the NPKM values of the sense transcripts (black) and antisense transcripts (red) plotted against the Rmet locus number. The values of the "-" strand were plotted with negative values. The position of the CMGIs is indicated. Deviations, which would lead to strong cluttering in the logarithmic scale, are not indicated.

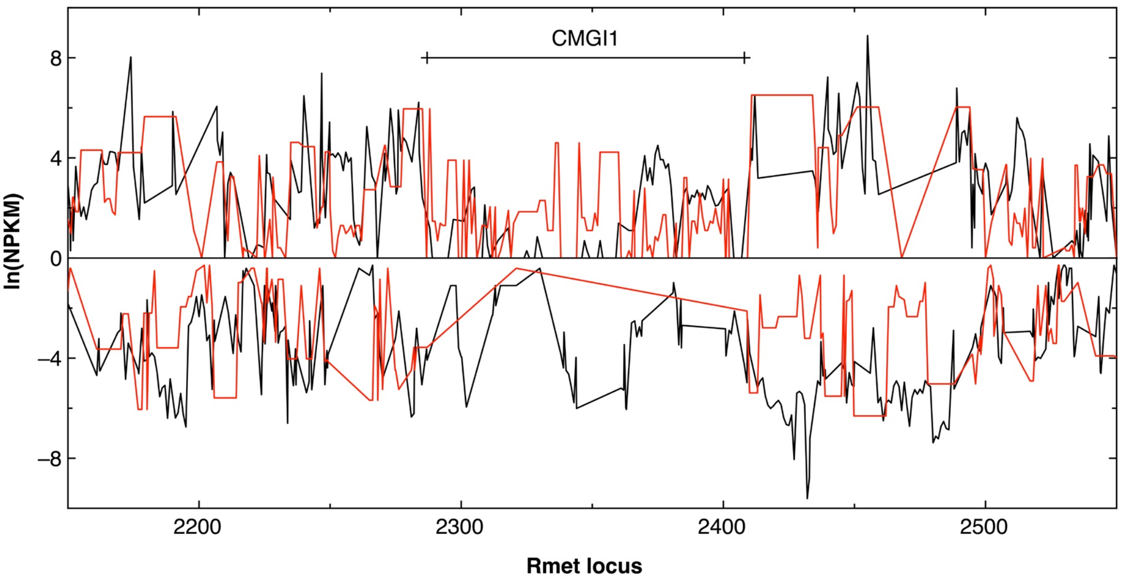

Supplementary Figure S1A: the region of CMGI1

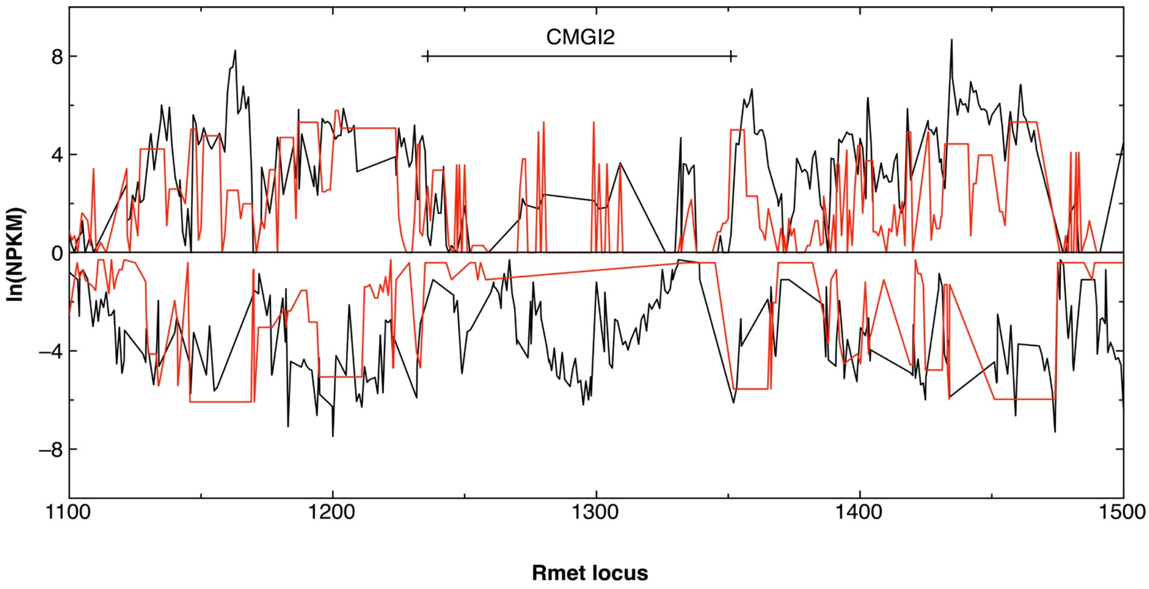

Supplementary Figure S1B: the region of CMGI2

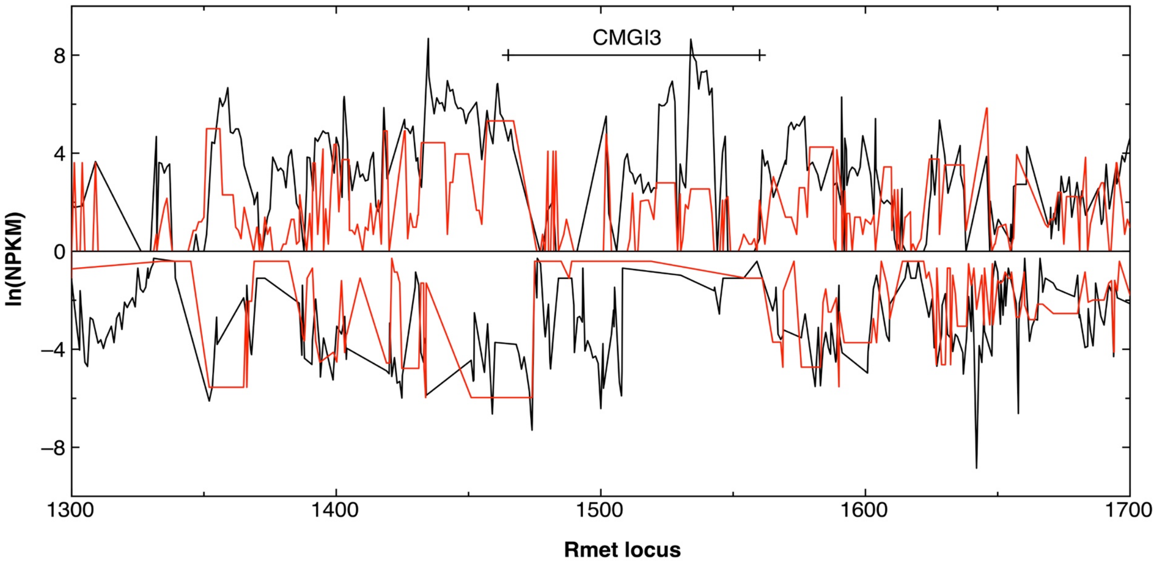

Supplementary Figure S1C: the region of CMGI3

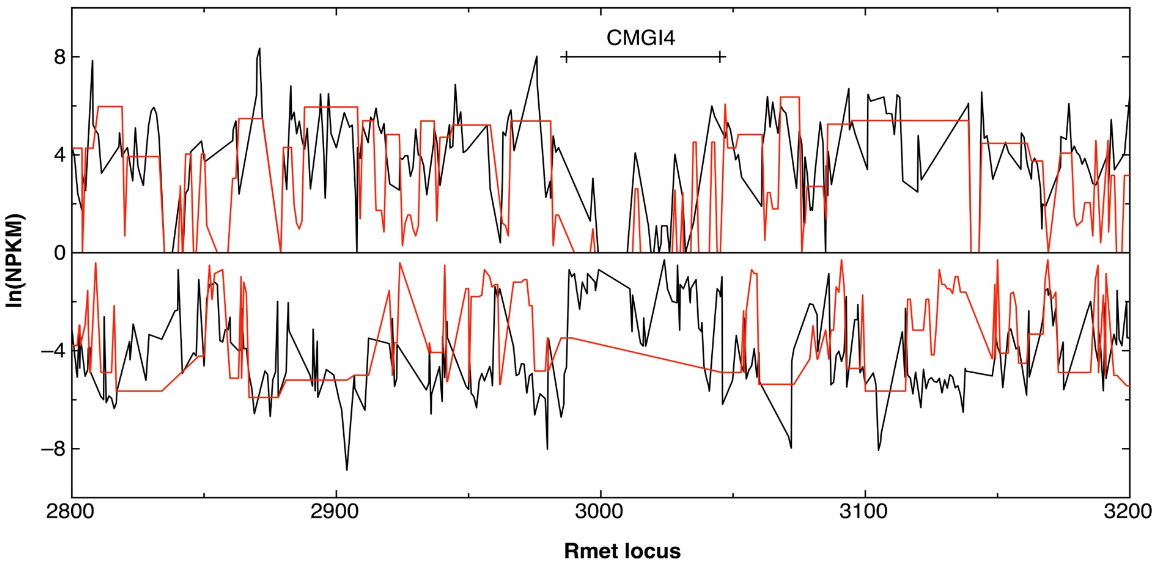

Supplementary Figure S1D: the region of CMGI4

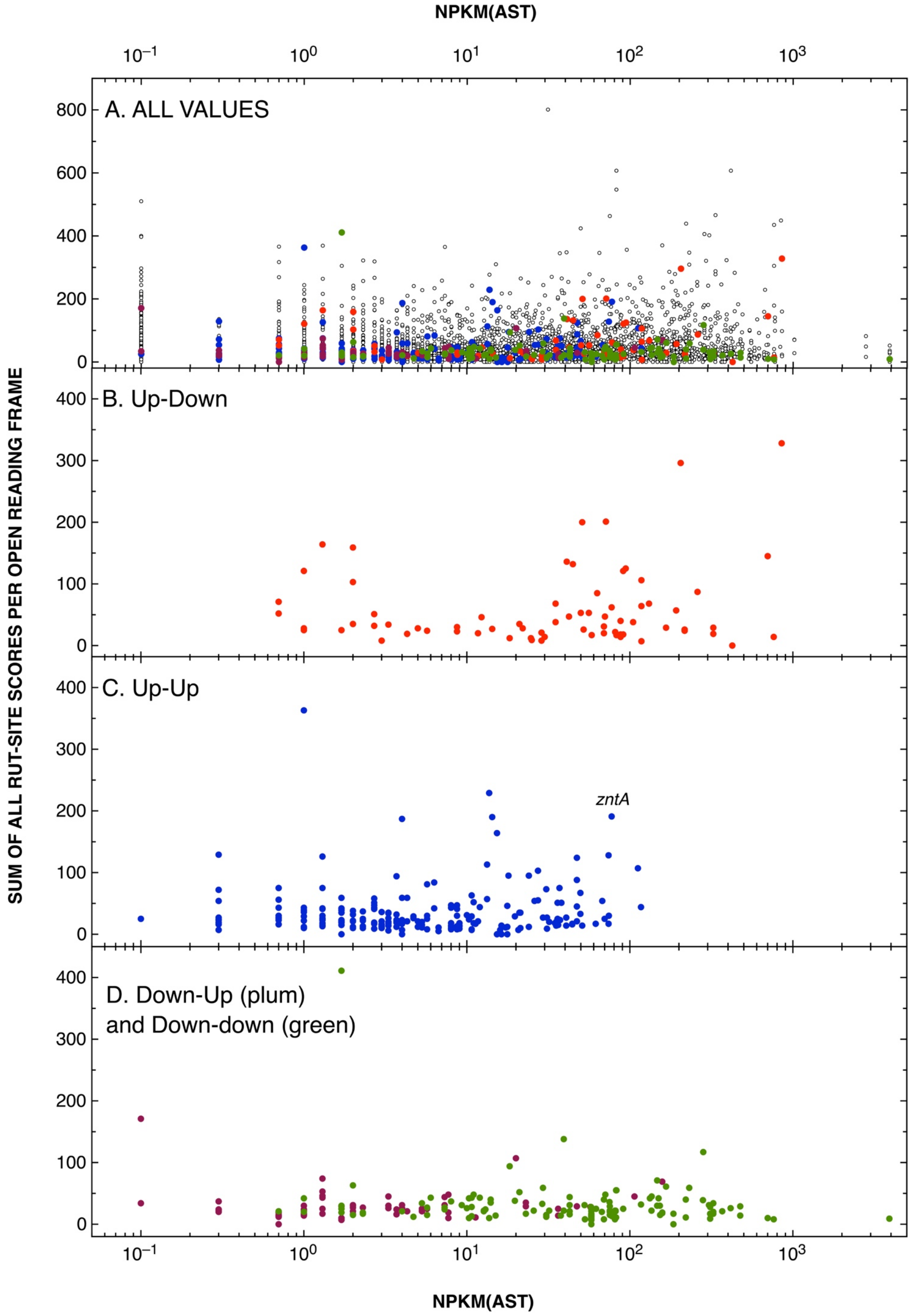

Supplementary Figure S2. Interplay of antisense and sense transcripts as well as Rho-dependent termination of sense transcripts. For all CH34 genes, the *rut* score was plotted against the abundance of associated antisense transcripts in non-challenged cells (Panel A, open circles). The data points were color-labeled if the respective sense transcripts up-regulated after treatment with the MultiTox metal mix were accompanied by a down-regulation of the associated antisense transcript (red circles) or down-regulated with respect to the AST (blue circles). In case of down-regulated sense transcripts, up-regulated associated antisense transcripts (plum-colored circles) or down-regulated ASTs (green circles). Panels B (up-down), C (up-up) and D (down-up, down-down) give only the results for metal-regulated genes. In the logarithmic plot of the x-axis, NPKM = 0 (no antisense transcript) was represented by the value 10^{-1}. The *zntA* gene for the major zinc-exporting P-type ATPase of CH34 is labeled in Panel C.

Antisense abundance and *rut* scores in metal-challenged cells. The plot of the *rut* score for each gene against the abundance of the antisense transcripts was repeated genes of *C. metallidurans* CH34 in the CH34_M_0 comparisons (Suppl. Fig. S2) that were up- or down-regulated and were accompanied by up- or down-regulated antisense activities. The results for down-regulated genes gave no pattern (Suppl. Fig. S2D) and was independent of the abundance of the AST transcript. For up-regulated genes with a down-regulated antisense activity, the *rut* scores increased with the abundance of the antisense transcript (Suppl. Fig. S3). Genes that were: (i) up-regulated under metal stress; (ii) accompanied by highly abundant antisense transcripts in non-challenged cells; and that were (iii) down-regulated upon metal stress, contained very high *rut* scores.

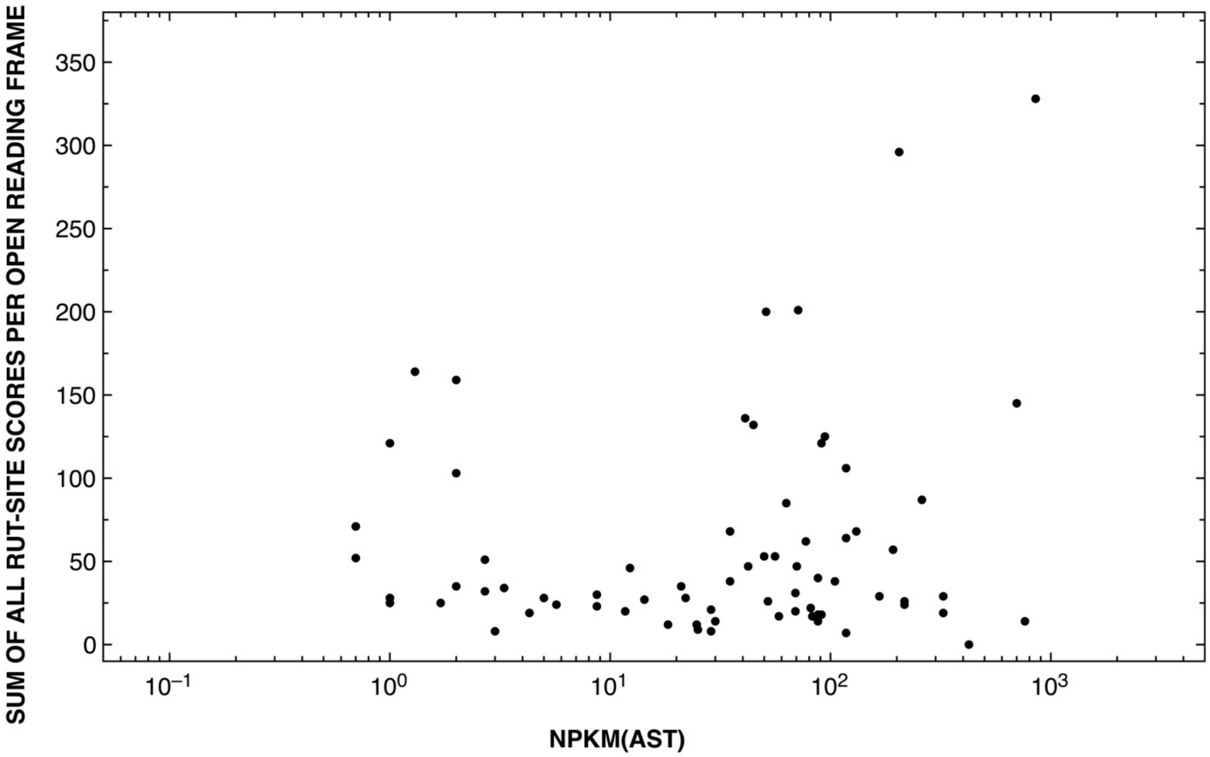

Supplementary Figure S3. Interplay of antisense and sense transcripts as well as Rho-dependent termination of sense transcripts. For all CH34 genes, the *rut* score was plotted against the abundance of associated antisense transcripts in non-challenged cells (Suppl. Fig. S2). This figure shows only the results for genes that were up-regulated after MultiTox metal mix treatment and accompanied by a down-regulated antisense abundance.

For up-regulated sense accompanied by up-regulated antisense transcripts (Suppl. Fig. S2C), the data points were shifted towards lower AST abundances. The data point that represents the highest *rut* score was associated with a predicted antisense transcript that was not expressed (NPKM = 1). For down-regulated genes (Suppl. Fig. S2D), the *rut* scores were lower than for up-regulated genes, with “down-up”-regulated shifted to lower antisense abundances than “down-down”-regulated gene values.

As judged by the *rut* score, there was an enrichment of *rut* sites in genes upregulated by metal stress associated with antisense activities, which were down-regulated by metal stress from a high abundance level in non-treated cells. Should the antisense transcript have the function to stabilize the sense transcript, loss of the antisense transcript would require an increased control of the transcription-translation coupling by Rho. On the other hand, up-regulation of both sense and antisense transcription would be hampered by Rho-dependent termination if the antisense RNA sequesters the sense RNA for translation at a later period of time.

On the other hand, should the antisense RNA destabilize the sense RNA, for instance by providing recognition sites for dsRNA-dependent RNAses, a highly expressed antisense RNA should down-regulate expression of the associated gene transcript. When the sense transcript is up- and the antisense transcript down-regulated, the strength of antisense-mediated repression would decrease, which would increase the importance of Rho-mediated transcription-translation coupling.

The respective up-down- and up-up-regulated genes encoded for instance ZneC (outer membrane factor of the Zne system), an integrase, a patatin, a transporter of the major facilitator superfamily, but no genes encoding the metal resistance determinants of *C. metallidurans* were identified. The only exception was *zntA* for the major zinc-exporting P-type ATPase of *C. metallidurans*, which was up-regulated by metal stress, accompanied by an also up-regulated antisense activity that exhibited a *rut* score of 191 (Suppl. Fig. S2C). The *zntA* antisense transcript AST_1215221+3 crossed *zntA* before it continued as a sense transcript of the *czcI*$_2$ gene and its 5UTR on the other DNA strand (Suppl. Mat. M2). While ZntA is the main zinc-exporting P-type ATPase, CzcI$_2$ regulates the activity of the CzcCBA efflux complex and both genes are under control of different regulatory circuits (10-12). To understand this complicated interaction between a possible function of the antisense RNA, Rho and the function of the gene products, a more detailed analysis of the sense and antisense levels in metal-treated CH34 cells will be required.

<u>Transcriptional network in *C. metallidurans* CH34 cells after treatment with the MultiTox metal-mix</u>. A number of 407 annotated genes among the 1,066 sense transcripts (Table 3) were up-regulated in the CH34_M_0 comparison (Suppl. Mat. M2). The remaining up-regulated transcript abundances were associated with 3UTRs and 5URTs. The 10 most strongly up-regulated (> 300-fold, Table 4) genes after metal shock were 7 metal resistance genes, one transposase, one sensor and an uncharacterized protein, both of unknown function (Table 4). All were accompanied by up-regulated antisense activities (Suppl. Mat. M2). The following 18 genes (> 100-fold) included 17 metal resistance genes plus one for a peptidase and 14 genes with up-regulated antisense activities. In their majority, the most strongly up-regulated genes encoded metal resistance factors and were accompanied by up-regulated antisense activities.

With decreasing up-regulation quotient, genes for other functions became more prominent and the ratio of genes that were not accompanied by up-regulated antisense activities increased.

Confirming the published gene array results (13), all active metal resistance determinants in CH34 were up-regulated following treatment with the MultiTox metal mix (Suppl. Mat. M2). Most determinants that encoded RND-driven trans-envelope efflux systems or components for them (*czc, czc_2, cnr, zni/zne, nimC, cus, sil*) were accompanied by antisense transcription activities (Table 5). Especially the sense transcripts of the major metal resistance determinants (*czc, cnr, cop, cup, chr* and *ars)* were accompanied by up-regulated antisense activities while recessive determinants (*nre/ncc, hmv, hmz*) were not. This strengthened our premise of the importance of the antisense activities for full regulatory function of the active and dominant metal resistance determinants. In comparison, the *cus* and *cop_2* determinants that were not plasmid-encoded were accompanied by down-regulated or unregulated antisense transcripts (Table 6). Plasmid-encoded metal resistance genes appear to need an up-regulated antisense transcript in addition to the up-regulated sense transcript.

All *czc* genes were up-regulated and accompanied by up-regulated antisense activities (Suppl. Mat. M2). The plasmid-encoded *czc* determinant contains a central *czcICBADRSE* region, which is also expressed under unchallenged conditions. Two additional genes (*czcM, czcN*) are located upstream of this central region on different DNA strands, and two downstream, on directly adjacent (*czcJ)* and one more distal (*czcP*)(Table 1). For the central *czcICBADRSE* region, the ASTs started at various positions on the opposite DNA strand and were below the threshold of NPKM = 10 in non-challenged cells. They were up-regulated more than 18-fold upon metal stress. Upstream of the central *czc* region, up-regulation of *czcN*-expression was accompanied by an up-regulated antisense activity that subsequently crossed the 5UTR of *czcN* to continue as sense transcript of the *czcM* gene in the opposite direction of transcription. The AST of *czcN* was up-regulated 10-fold from NPKM = 16 ± 2 to 158 while the sense transcript of *czcM* was up-regulated 5-fold from NPKM = 13 ± 2 to 70. These numbers would indicate that the AST of *czcN* and the mRNA of *czcM* were part of the same RNA molecule.

In a similar way, the antisense activity for *czcM* (NPKM = 51±7) initiated within the 3UTR of a Rmet_5986, upstream of *czcM* on the other DNA strand, continued across *czcM* as a sense transcript of *czcN* (NPKM 19±2 for the gene and 35±8 for its 5UTR) and the complete *czcICBADRSE* region (NPKM between 15 and 613) before being in antisense orientation to the 3UTR of *czcJ* on the other DNA strand. Up-regulation of the *czcM* AST was 12-fold to NPKM = 606, whereas the abundances of the sense transcripts of the *czcICBADRSE* region reached NPKM = 258 (*czcE*), 354 (*czcS),* 448 (*czcR*), 382 (*czcD*), 638 (*czcA*), 806 (*czcB*), 1,170 *(czcC*), 4,687 (*czcI*) and 996 (*czcN)* in metal-shocked cells (Supp. Mat. M2). These

numbers indicated the existence of many transcripts initiated from different transcriptional start sites on both DNA strands of the *czc* determinant or were processing products.

In a similar manner to what was observed for *czcN* and *czcM*, the antisense activity for *czcE* started within the transposase gene *tnpB*, continued as a sense transcript for the outer membrane genes *ompP* and *czcJ* before it reached *czcE* as its antisense transcript. Despite the strong, 133-fold up-regulation of *czcJ* following metal-stress, the *czcE* AST was only up-regulated 4-fold. This feature could be dependent on a high Rho-dependent termination frequency at *czcJ*, which had a *rut* score of 234. Finally, *czcP* for the high-rate zinc-exporting P_{IB4}-type ATPase had its own antisense activity that was up-regulated 840-fold under metal stress while the *czcP* sense transcript was up-regulated around 10-fold. For *czc*, a variety of FREE ASTs, double-use ASTs, sense transcripts and *rut* sites seemed to contribute to a controlled expression of this large and complicated plasmid-encoded metal resistance determinant.

A similar interaction network of up-regulated sense transcripts and antisense activities after metal shock was also observed for other metal resistance determinants (Table 1). For the interrupted “ancient” *czc*$_2$ region adjacent to *zntA*, the antisense activity (5-fold up-regulated from NPKM = 77 to 428) continued as a sense transcript of *czcI*$_2$ on the other DNA strand (26-fold upregulated from NPKM = 189 to 4,950), while the antisense activity for *czcI*$_2$ (49-fold from NPKM = 32 to 1,572) continued into *zntA* as a sense transcript (73-fold upregulated from NPKM = 42 to 3,052).

For the cobalt-nickel resistance determinant *cnr* on plasmid pMOL28, the antisense activities for the central region *cnrXHCBA* were independent of other transcripts. At the borders of the determinant, the antisense for the *cnrY* gene for the anti-sigma factor continued into an adjacent gene while that for the *cnrT* gene for an inner membrane nickel exporter continued from genes downstream of *cnrT* into this gene. The antisense activities may also interact with adjacent genes outside of the metal resistance determinants, in the case of *cnr* with the adjacent chromate-resistance determinant.

Other metal resistance determinants (Table 1) such as *chr*, *chr*$_2$ and *ars* were also up-regulated after metal stress (Table 5) and were accompanied in the case of most genes by up-regulated antisense activities. Up-regulated genes that were not part of metal-resistance determinants were *gshAB* (accompanied by down-regulation of the antisense activity), *isc* (down-regulation of the AST for *iscRS,* up-regulation of that for *iscUA-hscBA* and again down-regulation of the AST for *fdx* and the subsequent genes), the large phosphate cluster (upregulation of the antisense for *pstSCBA-phoUBR* and *ppk* for the polyphosphate kinase, while *ppx* for the polyphosphatase was not regulated in the sense and 2-fold-downregulated in the antisense activity) and the *gig* cluster. Other up-regulated genes encoded enzymes of the Entner-Doudoroff pathway for gluconate degradation, the Fe(II) efflux system, FieF (also

with up-regulated sense and antisense activities), the ABC exporter, AtmA (14), the sigma factor RpoN (with down-regulated antisense activities) and others (Suppl. Material M2).

These data demonstrate that following metal stress, metal resistance determinants are up-regulated, as expected, and that in most but not all cases this up-regulation is paralleled by an up-regulation of an associated antisense activity (Table 5). A similar pattern was exhibited by genes encoding phosphate uptake by the PtsABC transporter, iron-cluster and glutathione biosynthesis and the *gig* genes. In these cases, the respective gene products were needed to protect the cells against damage caused by the high concentration of toxic metals. An antisense activity that reduces expression of genes for such important proteins would be counterproductive. This suggests that for metal resistance genes, antisense transcripts may interact with sense transcripts to stabilize them.

Genes down-regulated following metal stress. The 10 most strongly down-regulated of the 654 genes (< 0.08-fold; only annotated genes expressed in non-challenged CH34 cells were counted, Table 4) included two TonB-dependent outer membrane receptors and a general porin. No gene was accompanied by any change in the potential antisense activities.

The next gene-set with a lower down-regulation quotient included genes involved in hydrogenase synthesis, in motility, respiratory chain and translation complexes. Expression of the genes for the membrane-bound hydrogenase, located in the large operon Op0370r_1 (Suppl. Mat. M2, Fig. 1. MbrHyd), were down-regulated between 0.1-fold (*hoxK*, small subunit, at the 5'-end of the operon) to 0.81-fold (not significant value, *hypD1*, penultimate gene). No antisense activity accompanied this operon. On the contrary, the gene region for the soluble, NAD^+-reducing and also nickel-containing hydrogenase plus the adjacent Calvin cycle-encoding genes were not down- or up-regulated under metal stress (Suppl. Mat. M2, Fig. 1, SolHydCC).

Not only were the genes for the membrane-bound hydrogenase down-regulated after metal shock, but also the *atp* genes for the F_1F_0-ATPase (0.23- to 0.48-fold, no change in the antisense activity), most *nuo* genes for the NADH-quinone oxidoreductase (*nuoCDEFGH* 0.44- to 0.48-fold, others 0.53- to 1.23-fold), *cox* genes for cytochrome *c* oxidase (0.67- to 0.82-fold), *cyo* genes for a cytochrome *o* ubiquionol oxidase (initially annotated as cytochrome *c* oxidase, 0.34- to 0.59-fold; no antisense effect), *app, cyd* and *cyo* genes for other respiratory chain components (0.18-fold down to unregulated; antisense 0.19-fold down for *cyoB* and 13.5-fold up-regulated for *cyoC*; others did not change). This indicated that metal shock resulted in a down-regulation of most membrane-bound proteins involved in energy conservation from the generation of the proton motive force to the synthesis of ATP (Fig. 1, OxPhs).

While the chemotaxis genes were only mildly down-regulated following metal shock (> 0.53-fold; no antisense effects), the *flh/flg* genes (0.18- to 0.31-fold for 11 genes, 6 genes 0.52-

to 0.92-fold; 3 genes with antisense up- and 2 genes with antisense down-regulation) and the *fla/fli* genes (9 genes 0.15- to 0.49-fold, 4 genes 0.54- to 0.68-fold; one antisense down-regulation) for the flagella were all down-regulated (Fig. 1, Flag). Metal shock with the subsequent synthesis of membrane-bound metal efflux systems resulted in the down-regulation of other membrane-bound proteins, the membrane-bound hydrogenase, flagella, F_1F_0 ATPase and respiratory chain components. While a lower content of flagella may help conserve proton motive force, the ability to build-up the proton motive force and to use it for ATP biosynthesis decreased upon metal stress, despite energy being needed for the function of the metal efflux systems.

Most genes of the tricarboxylic acid cycle, which provided NADH for the respiratory chain as well as anabolic metabolites (Fig. 1, TCA), were unchanged in expression with a mild down-regulation in expression of the genes for the membrane-bound succinate dehydrogenase (0.52- to 0.62-fold) and the other genes between 0.47- and 1.33-fold. Two genes were accompanied by down- (0.09-, 0.13-fold; *acnB* and *pckG*, respectively) and two genes by up- (1.69-fold, *fumA* and *fumB*) -regulated antisense activities. The pathway for the uptake and degradation of gluconate (Fig. 1, KDPG/FbP) was down-regulated with respect to the gene *gntT* for the transporter (0.43-fold, antisense 0.21-fold), the kinase gene *gntK* (0.30-fold; antisense unchanged) and the dehydratase *edd* (0.13-fold; antisense 0.19-fold). In the case of the dehydratase, another gene, *edd1*, was 4-fold up-regulated (antisense 1.9-fold) and its gene product likely took over the function of the *edd* gene product. All genes for the subsequent degradation pathway, from the aldolase gene *eda* to the components of the pyruvate dehydrogenase complex, were unchanged with the exception of the enolase gene (0.49-fold down-regulated) and the gene for the pyruvate dehydrogenase subunit AceE (3.23-fold; antisense 8.6-fold up-regulated for *aceE* and the adjacent *aceF*).

With respect to the assimilation of nitrogen and the subsequent synthesis of amino acids, expression of the genes for the glutamate dehydrogenase *gdhB* and the glutamine synthetase *glnA* was unchanged, while expression of *gltDB* encoding the glutamate synthase was down-regulated 0.37- and 0.30-fold, respectively. Together with GlnA, glutamate is synthesized by an ATP-consuming pathway while the direct assimilation of ammonium by GdhB depends only on NADH. Nitrogen assimilation was shifted to a less energy-consuming but also less efficient pathway. The genes for the aminotransferases were unchanged and only the genes encoding three aminoacyl-tRNA synthetases were slightly down-regulated (*lysS* 0.38-, *pheS* 0.49- and *ileS* 0.44-fold), with some scattered antisense activities among the genes for the tRNA synthetases (Fig. 1, Suppl. Mat. M2).

After metal shock, expression of the genes encoding enzymes for the degradation of gluconate to the level of acetyl-CoA and those for the synthesis of amino acids up to the tRNA synthesis were changed little, except that the initial input reactions (*gntT, gntK, edd)* for carbon

from gluconate and nitrogen via the ATP-dependent GlnA-GltDB enzymes were down-regulated. This may decrease the overall flow of metabolites from gluconate to amino acids loaded to their tRNAs.

The genes for the ribosomal proteins were all down-regulated 0.4-fold (0.13- to 0.79-fold, down-regulation of an antisense activity covering the genes for 25 ribosomal proteins, Fig. 1). Expression of the genes encoding the translational initiation factors was mildly down-regulated (0.49- to 0.65-fold), while that of the genes encoding the elongation factors was more strongly down-regulated (*efp* 0.39-, *fusA*$_1$ 0.35- and *tuf* 0.46-fold for elongation factors P, G and Tu, respectively, Fig. 1). Reminiscent of *edd*, *fusA*$_2$ expression was up-regulated 5.5-fold with an accompanying 31-fold up-regulation of an antisense activity, so that the chromid-encoded FusA$_2$ may support a down-regulated FusA$_1$ as elongation factor G, which moves the translating ribosome one codon ahead.

Regarding the genes whose products are involved in protein export, expression of *ffh* and *ftsY* encoding membrane integration via the signal recognition particle was not down-regulated but expression of all genes for the general secretion pore were, including the leader peptidases and YidC (0.39- to 0.54-fold), all except *secA*. Expression of the genes encoding the twin arginine transport pathway was also unaffected (Suppl. Mat M2). This indicates that the energy-consuming translation and protein export by the general secretion pathway was down-regulated following metal shock (Fig. 1).

Compared to the genes for the ribosomal proteins, no gene for subunits of the DNA polymerase III or the other DNA polymerases were down-regulated. DNA replication and repair were not impaired. In contrast (Table 7), the genes for the RNA polymerase core enzyme, the *nus* and *gre* factors, *rho* and the major house-keeping sigma factor *rpoD*$_1$ were all down-regulated between 0.34- and 0.79-fold (mean value 0.55-fold), with exception of *rpoZ* (mildly up-regulated 1.4-fold). For *rpoB, rpoC, nusG, greA* and *rpoD*$_1$, this was accompanied by an up-regulation of the associated antisense activity, for *rpoA* and *nusB* with a down-regulation. The *rapA* gene (15, 16) was barely expressed in non-challenged CH34 cells (NPKM = 11±1.2), was unregulated in metal-challenged cells and possessed a strong antisense activity, which was also not regulated in metal-shocked CH34 cells. Among the genes for the alternative sigma factors, *rpoH, rpoN, rpo*Q and *cnrH* were up-regulated, *cnrH* accompanied by a 361-fold up-regulation of its antisense activity. Only the chemotaxis and motility sigma factor *fliA* was down-regulated (Table 7). Not only the translation but also the general transcription activity of metal-shocked *C. metallidurans* cells was down-regulated, possibly to conserve energy by decreasing the energy-intensive process of protein biosynthesis.

In total, multiple metal shock of *C. metallidurans* CH34 led to an up-regulation of metal resistance determinants as expected, in most cases accompanied by an up-regulation of antisense activities. These determinants encode a multitude of membrane-bound metal efflux

systems. Simultaneously, the genes for the F_1F_0 ATPase and respiratory chain components are down-regulated, which counterintuitively should decrease the amount of available energy. Consequently, high energy-consuming processes such as motility, transcription, translation and protein export are down-regulated, probably to save sufficient energy to drive or synthesize the efflux systems. These processes also lead to a higher competition of the efflux systems, F_1F_0 and the respiratory chain components for protein export capacity by the general secretion pore (Fig. 1).

Adaptation to metal starvation in CH34 wild type. The top ten of the 34 up-regulated genes (> 5-fold) identified in *C. metallidurans* after treatment with EDTA (CH34_E_0 comparison) were difficult to interpret in terms of roles in metal-limitation (Table 4), except for possibly the 5.15-fold up-regulation of *groS* encoding a chaperonin, indicating possible problems with protein folding under this stress. Other genes with a reduced up-regulation quotient included *zwf* (4.26-fold; glucose-6-phosphate 1 dehydrogenase), *bfr* (3.21-fold; bacterioferritin), *zniA* and *zniB* (2.5-fold; RND-driven transport system) and *cysS* (2.17-fold) as representative of the *cobW*$_1$ zinc starvation cluster. The genes in this cluster were up-regulated about 2-fold. The accompanying antisense activities of the up-regulated genes displayed no pattern with 6 antisense activities down- and three up-regulated.

Similarly, the top ten of the 63 down-regulated genes (≤ 0.23-fold) also did not yield many insights (Table 4). These were mostly genes encoding regulators. The subsequent genes in the list were the genes *zntA* (0.40-fold), *cadA* (0.48-fold), which encoded two P-type ATPases, and the *czc* genes, including *czcD* (0.41- to 0.46-fold, Table 7), indicating that efflux systems for transition metals were down-regulated under conditions of metal starvation. The *edd*$_1$ gene product, which was up-regulated in metal-shocked cells, could substitute for its paralog, the *edd* gene product, and was down-regulated 0.44-fold. With the exception of the genes for efflux systems, no gene for a metabolic process was evident that was down-regulated under EDTA-induced metal starvation stress. There was also little change in the antisense patterns of the down-regulated genes.

Among the genes for transport proteins (Table 8), only *zupT* was up-regulated (1.63-fold). None of the genes for the siderophore biosynthesis cluster downstream of the sigma factor genes *rpoI* was up- or down-regulated so that the EDTA-treated cells did not suffer from iron starvation after 10 min of incubation (Suppl. Mat. M2, “Transport”). This was supported by the unchanged amount of iron in these cells (Table 2). Except for *zntA, cadA* and *czcD,* none of the genes for efflux systems, which were up-regulated following metal shock, was down-regulated in EDTA-treated cells. The “anabolic” Cu(I)-exporting P-type ATPase genes *rdxI* and *ctpF* and the Co(II) exporter gene *dmeF* were not up-regulated following metal stress but the *ctpF* antisense activity was strongly up-regulated (15-fold) under metal stress. There was not

much change in the metal transportome of the inner membrane under conditions of metal starvation, except for the down-regulation of *zntA, cadA* and *czcD* (Table 8).

Comparing the composition of the outer membrane proteins in *C. metallidurans* after metal shock or EDTA treatment, the former resulted in a down-regulation of 7 porin genes (0.09- to 0.37-fold), while two *ompP* paralogs were up-regulated (5.3- and 2.1-fold), one of these paralogs revealed a 130-fold up-regulation of its associated antisense activity. One porin was up-regulated after EDTA treatment (Rmet_1733, 3-fold). Four sets of genes encoding TonB-dependent receptors were down-regulated after metal shock (0.03- to 0.27-fold) and one was up-regulated (*piuA* 2.22-fold). Following EDTA treatment, the TonB-dependent Rmet_0123 was strongly (11-fold) up-regulated. This indicated that *C. metallidurans* changed the composition of the general porins following metal stress and may produce a TonB-dependent receptor for import of essential ions (Suppl. Mat M2, Transport).

Following transport into the cytoplasm, these ions may be further imported into the cytoplasm by an ABC transport system. While a variety of *phn-, ptx-* (phosphonate), and *pst-* (phosphate) ABC importer genes were up-regulated under metal shock, none of the genes up-regulated under metal starvation conditions was able to reach an expression level higher than the threshold of NPKM = 10 (Suppl. Mat. M2, Transport).

With the exception of the TonB-dependent Rmet_0123, the already present metal transportome plus some Zur-dependent up-regulation of *zupT* and the *cobW1* cluster may be sufficient for *C. metallidurans* to survive EDTA-mediated metal starvation. In contrast to metal resistance determinants that counteracted metal shock, no "starvation-resistance" determinants seem to exist for non-iron transition metals in *C. metallidurans*, except the Zur regulon. Moreover, no expression change for the sigma factors was observed (Table 7) with the exception of up-regulation of the antisense activities for the plasmid-encoded *cnrH*. It is, however, the question whether this low impact of the EDTA treatment on the transcriptome was not only the consequence of the short incubation time of 10 min, which might not allow the cells the really "feel" the starvation.

<u>Differences between unchallenged cells of CH34 and AE104</u>. In the comparison of non-challenged control cells, only 15 genes were up-regulated (Q > 2, D > 1, gene annotated) in the plasmid-free derivative AE104 compared to the wild type, and 5 genes were down-regulated. The up-regulated genes included the ortholog *czcC*$_2$ of the outer membrane factor of the Czc trans-envelope efflux system for zinc, cobalt and cadmium, which was also expressed in non-challenged CH34 cells. With loss of the plasmid-encoded mercury resistance determinants, expression of the gene for the regulator of the chromosomal *mer* determinant *merR* also increased, as did the operon Op1498f_1 encoding the ABC importer for peptides Dpp. The few down-regulated genes (Q > 2, D > 1, NPKM > 10 in unchallenged CH34 cells)

encoded a transposase, a cold-shock protein and the TonB-dependent receptor Rmet_0123. Expression of the receptor gene was accompanied by a strong up-regulation of the antisense activity, which was independent from any sense transcripts on its own DNA strand.

Expression of the *dpp* genes at the 3' end of the operon was accompanied by down-regulated antisense activities, which were also independent of sense transcripts from their encoding DNA strand. Expression of the genes for the metal efflux and uptake systems were not different in both strains (Table 8). Expression of genes encoding the siderophore synthesis, TonB-dependent receptors (except Rmet_0123 and some antisense activities), the porins (except 2 antisense activities) and ABC transporters (both categories with the exception of some antisense activities) was also unchanged (Supp. Mat. M3, "Transport"). There was also no change in the expression of sigma factors and other genes involved in transcription (Table 7), except the now missing plasmid-encoded *cnrH* and strong down-regulation of four antisense transcripts for *rpoS, rpoM, rpoO* and *rpoP.* With the exception of Rmet_0123, $CzcC_2$ and of course the missing plasmid-encoded efflux systems, expression of the genes encoding metal transportome and sigma-regulons of unchallenged plasmid-free cells was similar to that in CH34 wildtype cells, and the expression of the chromosomal *merR* regulator gene is primed to activate expression of the chromosomal *mer* determinant, should the cells be exposed to Hg(II). Otherwise, the plasmid-encoded metal resistance determinants had little impact on cells living under non-challenging conditions.

Differences between metal-shocked CH34 and AE104 cells. Cells of the plasmid-free derivative AE104 and its wildtype CH34 were challenged with two strain-specific metal mixes to address their individual metal resistance. Examining genes exhibiting up- ($Q > 2$, $D > 1$, annotated genes) and down-regulated expression ($Q < 0.5$, $D > 1$, NPKM > 10 in CH34_0, annotated genes), 305 genes were up-regulated and 159 genes were down-regulated in AE104 compared to CH34 (Table 4). The top ten of the up-regulated genes, of them with $Q > 10$, contained one gene for a glutamine synthetase. This gene was down-regulated upon metal shock in CH34 cells. In total, 230 genes of the 305 genes up-regulated in AE104 were down-regulated ($Q < 0.7$) in metal-shocked CH34 cells. These included 31 genes encoding ribosomal proteins, elongation factor P, some flagellar components, SecE and SecG involved in protein export, the RNA polymerase-associated factors GreA and NusG, components of the respiratory chain complexes, the F_1F_0 ATPase, ABC importers for phosphate, phosphonate and the 6-phosphogluconate dehydratase, one of the initial steps of the KDPG pathway for gluconate degradation. With respect to the ribosomal gene transcripts, the mean value of the products Q_down-regulated in CH34 times Q_up-regulated in AE104 was 83.1 ± 13.9%. This indicated that 75% of the up-regulated genes in AE104 nearly reached the same transcript abundances in metal-stressed AE104 cells as in non-challenged CH34 cells. This means that

the plasmid-encoded metal resistance determinants were responsible for the energy shortfall and its consequences in strain CH34.

In 38 cases, genes already up-regulated in metal-stressed CH34 cells were expressed at an even higher abundance in metal-stressed AE104 cells. These were components of the chromosomal or chromid-encoded metal resistance determinants *cus*, cop_2 (Table 6, Figs. 3 and 4), and especially *mer*, which was up-regulated already 4,000-fold in CH34 and now again additionally two-fold in AE104 when the plasmid-encoded *mer* determinants were absent (Suppl. Mat. M2). In AE104 cells, with the exception of the gene encoding the response regulator HmzR, no component of the recessive RND-driven trans-envelope systems was up-regulated above the level determined in metal-challenged CH34 cells. These systems did not compensate for the plasmid-encoded systems. There was also no change in the expression of the genes for the metal uptake systems, with one exception: *pitA* was 0.34-fold down-regulated in metal-challenged CH34 cells and 2.44-fold up-regulated in metal-challenged AE104 cells (Table 8), nearly reaching its abundance in un-challenged CH34 cells. The antisense activity of $corA_1$ was 50-fold up-regulated in metal-challenged AE104 cells.

Comparing the abundances of the efflux systems in metal-challenged AE104 to CH34 cells, *zntA* was down-regulated 0.25-, *cadA* 0.08-, *cupA* 0.42- and *atmA* 0.40-fold. On the other hand, these genes were up-regulated metal-challenged AE104 to non-challenged AE104 cells 18.3-, 4.96-, 53.1- and 1.47-fold, respectively. Thus, with the exception of *atmA,* these genes were also up-regulated in AE104 but to a lower level than in CH34 cells (Table 8). This agreed with the lower concentrations of the challenging metals (Table 2). Despite the lower concentration of challenging metal ions and the absence of the plasmid-encoded metal resistance determinants, sufficient zinc, copper and cadmium reached the cytoplasm of strain AE104 to yield a ZntR-, CadR- and CupR-dependent up-regulation of *zntA, cadA* and *cupA*, respectively.

There were also genes encoding two TonB-dependent receptors and 3 porins whose expression levels were up-regulated in AE104_M and down-regulated in CH34_M (Suppl. Mat. M2). With respect to the TonB-dependent receptors, antisense activities for six sense transcripts were down-regulated and for three were up-regulated. Among the 159 gene down-regulated transcripts in AE104_M, most (126) were up-regulated in CH34_M. In most of these cases, the down-regulation in AE104_M compensated for the up-regulation in CH34_M. The genes following this pattern were metal-resistance genes *cadA, cadC,* $chrF_3$*, zntA, atmA, cupA,* $czcI_2$*, zniCBA,* for the regulators *zntR,* $czcR_2$*, zneR, phoU, phoR, cupR*. Except *atmA,* $czcR_2$ and *phoR*, the products of the up-regulation in CH34_M and down-regulation in AE104_M were greater than 2, so that these genes were up-regulated in AE104_M but not as strongly as in CH34_M, again as an answer to the lower concentration of the individually challenging metal ion concentrations. Other genes following this pattern were the components

of the Pst phosphate-specific ABC importer and *fusA*$_2$ for the elongation factor G. Genes regulated in the opposite direction, down-regulated in CH34_M and even more so in AE104_M, encoded components of the membrane-bound hydrogenase and the TonB-dependent receptor OprC.

There was no change in the expression of the genes for sigma factors but an up-regulation of the genes for the RNAP-associated proteins NusG and GreA was determined. The antisense activities of 3 sigma factor genes were down-regulated (*rpoD*$_1$ for the main housekeeping sigma factor, *rpoJ, rpoO)* and two were up-regulated (*rpoN, rpoM*).

There were no genes evident that were not expressed in metal-challenged CH34 cells but were expressed in metal-challenged AE104 cells. No additional genes compensated for loss of the plasmid-encoded metal resistance determinants, for instance the recessive RND-encoding, inactivated determinants. Due to the lower concentration of the challenging metals used, the non-plasmid-encoded resistance determinants were even less up-regulated in strain AE104 compared to wildtype CH34. This agrees with the observation that strain AE104 simply loses metal resistance when losing the plasmids pMOL30 and pMOL28 (17).

Comparison of strains CH34 and AE104 under conditions of metal starvation. In total, only 7 genes were up-regulated in the comparison between EDTA-treated AE104 and CH34 cells, among them again was *merR* for the chromosomal copy of the *mer* regulator (Table 4). Even the 4 genes that were expressed above NPKM = 10 in unchallenged CH34 cells were down-regulated in these cells, encoding for instance the TonB-dependent receptor Rmet_0123 (Table 4). There was no change in the sense transcripts or antisense activities for the genes encoding the metal uptake or efflux systems (Table 8), the porins, the TonB-dependent receptors except Rmet_0123 or the ABC transport systems, but changes in the antisense activities were noted (Supp. Mat M2). These were for the siderophore synthesis cluster, some TonB-dependent receptors, including a very strong 0.01-fold down-regulation of the antisense activity of Rmet_0123 and some down-regulation of four other transcripts of these genes, a down-regulation of the antisense activities for *phoU* and *pstB* as well as a 68-fold up-regulation of the antisense activity of *cysP* for a thiosulfate-binding protein.

The only noted expression changes concerning genes encoding the RNAP and its sigma factors were changes in the expression of antisense activities for some sigma factors. The antisense activities for *rpoN*, which had been up-regulated in the comparison of metal-shocked cells, was down-regulated in the comparison of EDTA-treated cells, while the antisense for *rpoP* displayed the inverse pattern. The sense or antisense for *rpoO* was not changed in CH34 cells but its antisense showed some kind of metal-dependent titration pattern, completely absent in the comparison of metal-treated cells, 0.25-fold down-regulated in that of non-challenged cells and 0.33-fold down-regulated in that of EDTA-treated cells. This

indicated that antisense transcripts may be involved in control of the expression of sigma factor genes in *C. metallidurans*.

Supplementary Table S5. Transcriptional landscape for six selected genes. The table gives the details of the transcripts as determined by RNASeq. The elements are the transcriptional start sites TSS, the asRNAs AST and their respective stop position STP_AST, the genes NPKM and 3UTR for an untranslated region. Red indicates TSSs from the "+" strand, blue from the "-" strand. The yellow area indicates the RT-PCR product from the asRNA of that region. Shaded asRNAs were measured in RNA isolated from metal-induced cells of C. metallidurans CH34, others from cells not treated with metals. The locus tag and names of the genes are also provided. Published TSS [Große, 2022 #498] are given with the sigma factor responsible for transcription initiation. The last 4 rows contain the intensity of the signals, NPKM values for the genes, 3UTR and asRNAs and scores for the TSSs; n.f. no signal found. Three determinations. When for a TSS one value is given, this means that only one signal appeared in the three experiments. CO, CH34 control, CM, metal-treated cells. Promoters, green field "s" for RpoD model, yellow field "m" moderate probability for RpoD, others no RpoD model.

Cnr

Element	start	strand	stop	length	replicon/TSS	locus tag/Prom	gene	MEAN_C0	SDEV_C0	MEAN_CM	SDEV_CM
**STP_AST_56260-5**	**43266**	**-**	**43266**	**12994**	**CP000355**					**1083.3**	**159.5**
TSS_53286+5	53286	+			Pub	s		2918	1624	28233	2977
NPKM_6205	53309	+	53597	288	CP000355	Rmet_6205	*cnrY*	171.3	55.6	1080.67	177.51
STP_AST_54298-5	53435	-	53435	863	CP000355			3.00	0.0		
NPKM_6206	53593	+	54040	447	CP000355	Rmet_6206	*cnrX*	99.3	30.0	726.00	115.75
NPKM_6207	54036	+	54612	576	CP000355	Rmet_6207	*cnrH*	61.0	13.7	466.33	101.04
**RT-PCR_cnrH**	**54162**		**54287**	**125**							
AST_54298-5	54298	-	53435	863	CP000355			3.00	0.0		
TSS_54822-5	54822	-			NotPub	CH34_M		0.00		50	16
NPKM_6208	54684	+	55941	1257	CP000355	Rmet_6208	*cnrC*	48.3	13.4	255.00	30.35
TSS_54955-5	54955	-			NotPub	CH34_M		0.0		23	5
TSS_56237-5	56237	-			Pub	s		32		0	
**AST_56260-5**	**56260**	**-**	**43266**	**12994**	**CP000355**					**1083.3**	**159.5**
NPKM_6209	55937	+	57125	1188	CP000355	Rmet_6209	*cnrB*	26.0	4.6	150.00	19.92
NPKM_6210	57121	+	60352	3231	CP000355	Rmet_6210	*cnrA*	29.7	5.0	168.33	10.07
TSS_57840-5	57840	-			NotPub	CH34_E		30	16	0	
TSS_58867-5	58867	-			Pub	s		158	12	212	37
TSS_59074-5	59074	-			NotPub	CH34_M		25	6	107	24
TSS_60287-5	60287	-			NotPub	CH34_M		81	14	231	20
NPKM_6211	60397	+	61453	1056	CP000355	Rmet_6211	*cnrT*	47.3	8.3	230.00	5.29
TSS_60510-5	60510	-			NotPub	CH34_M		29	6	102	24
TSS_60580-5	60580	-			NotPub	CH34_M		45	12	109	30
3UTR_6211	61454	+	63472	2018	CP000355	Rmet_6211	*cnrT*	12.3	1.2	29.00	5.00
TSS_63507-5	63507	-			NotPub	CH34_M		35	6	51	7

CzcP

Element	start	strand	stop	length	replicon	locus tag	gene	MEAN_C0	SDEV_C0	MEAN_CM	SDEV_CM
TSS_88714+4	88714	+			NotPub	CH34_M		0.0		46	7
**STP_AST_101723-4**	**88625**	**-**	**88625**	**13098**	**CP000354**					**560.0**	**53.7**
STP_AST_89550-4	88711	-	88711	839	CP000354			7.33	2.1		
5UTR_5970	89415	+	89604	189	CP000354	Rmet_5970	*czcP*	6.3	1.2		
TSS_89553+4	89553	+			NotPub	CH34_M		24	6	1202	179
TSS_89563+4	89563	+			Pub	*no*		129	15	2639	271
TSS_89441-4	89441	-			Pub	s		0		478	426
AST_89550-4	89550	-	88711	839	CP000354			7.33	2.1		
TSS_92052-4	92052	-			NotPub	CH34_0		23	3	0	
NPKM_5970	89604	+	92094	2490	CP000354	Rmet_5970	*czcP*	7.7	1.5	74.67	1.15
STP_AST_92956-4	90958	-	90958	1998	CP000354			14.33	1.5		
RT-PCR_czcP	91841		91978	137							
3UTR_5970	92095	+	92869	774	CP000354	Rmet_5970	*czcP*	5.7	0.6	32.00	2.00
TSS_92062-4	92062	-			NotPub	CH34_0		27	6	0	
TSS_92535-4	92535	-			Pub	s		277	12	167358	14211
AST_92956-4	92956	-	90958	1998	CP000354			14.33	1.5		
TSS_96132-4	96132	-			Pub	*no*		280	49	1168	150
TSS_97054-4	97054	-			Pub	*no*		669	206	2395	828
TSS_97146-4	97146	-			NotPub	CH34_M		74	4	125	12
TSS_97206-4	97206	-			Pub	s		2718	642	12288	1457
TSS_98167-4	98167	-			NotPub	CH34_M		129	12	380	30
TSS_98671-4	98671	-			Pub	*no*		149	76	319	35
TSS_98681-4	98681	-			NotPub	CH34_M		21	1	80	14
TSS_99109-4	99109	-			NotPub	CH34_M		33	4	18	1
TSS_100199-4	100199	-			Pub	*no*		1437	309	3355	308
TSS_100887-4	100887	-			Pub	m		368	43	881	112
TSS_101020-4	101020	-			Pub	*no*		270	92	2898	91
TSS_101042-4	101042	-			NotPub	CH34_M		77	11	319	27
**AST_101723-4**	**101723**	**-**	**88625**	**13098**	**CP000354**					**560.0**	**53.7**
TSS_103614-4	103614	-			NotPub	CH34_E		23	5	0	

ZniR

Element	start	strand	stop	length	replicon	locus tag	gene	MEAN_C0	SDEV_C0	MEAN_CM	SDEV_CM
**STP_AST_2034583-3**	**2024507**	**-**	**2024507**	**10076**	**CP000353**					**282.3**	**29.1**
**TSS_2030010+3**	2030010	+			Pub2	s		293	152	798	281
STP_AST_2034515-3	2030380	-	2030380	4135	CP000353			16.33	1.5		
3UTR_5322	2030886	-	2031609	723	CP000353	Rmet_5322	*zniS*	4.0	1.0	5.67	2.08
NPKM_5322	2031610	-	2033200	1590	CP000353	Rmet_5322	*zniS*	13.3	0.6	18.00	2.00
NPKM_5323	2033309	+	2033930	621	CP000353	Rmet_5323	*zniR*	129.0	3.0	137.00	10.00
5UTR_5323	2033134	+	2033309	175	CP000353	Rmet_5323	*zniR*	14.7	1.2	11.00	2.65
RT-PCR_zniR	2033369		2033495	126							
NPKM_5324	2033937	-	2034249	312	CP000353	Rmet_5324		8.7	2.1	10.67	2.52
TSS_2034263-3	2034263	-			NotPub	CH34_M		0		32	6
TSS_2034321-3	2034321	-			Pub	*w*		27	11	26	4
AST_2034515-3	2034515	-	2030380	4135	CP000353			16.33	1.5		
5UTR_5324	2034250	-	2034515	265	CP000353	Rmet_5324		11.7	2.3	9.00	1.73
**AST_2034583-3**	**2034583**	**-**	**2024507**	**10076**	**CP000353**					**282.3**	**29.1**
TSS_2037201-3	2037201	-			NotPub	CH34_M		0		68	6

CopN

Element	start	strand	stop	length	replicon	locus tag	gene	MEAN_C0	SDEV_C0	MEAN_CM	SDEV_CM
**STP_AST_195530-4**	**189324**	**-**	**189324**	**6206**	**CP000354**					**185.0**	**17.3**
5UTR_6111	190986	+	191402	416	CP000354	Rmet_6111	*copR1*	18.3	2.5	31.33	7.51
TSS_190988+4	190988	+			Pub			127	30	114	37
TSS_191305+4	191305	+			NotPub	CH34_E		50	12	0	
TSS_191346+4	191346	+			Pub	*no*		406	18	63800	11337
TSS_191356+4	191356	+			NotPub	CH34_M		0		63	15
NPKM_6111	191402	+	192089	687	CP000354	Rmet_6111	*copR1*	24.0	4.4	621.00	40.15
TSS_191547+4	191547	+			NotPub	CH34_M		21	1	109	24
NPKM_6110	192085	+	193477	1392	CP000354	Rmet_6110	*copS1*	27.7	4.5	585.67	26.41
STP_AST_193651-4	192708	-	192708	943	CP000354			6.67	0.6		
TSS_192980+4	192980	+			Pub	*(w)-sba*		210	40	721	17
TSS_193060+4	193060	+			NotPub	CH34_M		0		22	1
**TSS_193249+4**	193249	+			Pub	m		399	62	1612	227
NPKM_6109	193527	+	194022	495	CP000354	Rmet_6109	*copN*	29.0	5.6	451.33	39.11
AST_193651-4	193651	-	192708	943	CP000354			6.67	0.6		
STP_AST_195153-4	193719	-	193719	1434	CP000354			7.67	0.6		
RT-PCR_copN	193865		194024	159							
3UTR_6109	194023	+	195366	1343	CP000354	Rmet_6109	*copN*	10.0	1.0		
TSS_194343-4	194343	-			NotPub	CH34_M		60	14	210	45
TSS_194715-4	194715	-			NotPub	CH34_M		0		66	16
TSS_194769-4	194769	-			Pub			92	16	25820	4420
TSS_194841-4	194841	-			NotPub	CH34_M		0		57	18
AST_195153-4	195153	-	193719	1434	CP000354			7.67	0.6		
**AST_195530-4**	**195530**	**-**	**189324**	**6206**	**CP000354**					**185.0**	**17.3**
TSS_197125-4	197125	-			Pub	s		683	164	1926	427

CopR2

Element	start	strand	stop	length	replicon	locus tag	gene	MEAN_C0	SDEV_C0	MEAN_CM	SDEV_CM
**STP_AST_2431948-3**	**2425619**	**-**	**2425619**	**6329**	**CP000353**					**57.3**	**5.9**
5UTR_5672	2431115	+	2431332	217	CP000353	Rmet_5672	*copR2*	29.3	5.5	26.33	1.15
**TSS_2431185+3**	2431185	+			Pub	s		102	5	177	18
STP_AST_2431777-3	2431138	-	2431138	639	CP000353			8.33	0.6		
TSS_2431257+3	2431257	+			Pub	*no*		0		0	
TSS_2431286+3	2431286	+			Pub	*no*		206	53	1525	229
NPKM_5672	2431332	+	2432019	687	CP000353	Rmet_5672	*copR2*	9.7	1.2	53.33	8.14
RT-PCR_copR2	2431401		2431571	170							
AST_2431777-3	2431777	-	2431138	639	CP000353			8.33	0.6		
**AST_2431948-3**	**2431948**	**-**	**2425619**	**6329**	**CP000353**					**57.3**	**5.9**
NPKM_5673	2432018	+	2433407	1389	CP000353	Rmet_5673	*copS2*	5.3	1.2	39.33	3.21
3UTR_5673	2433408	+	2433526	118	CP000353	Rmet_5673	*copS2*	2.0	1.0	10.00	1.00
TSS_2434874-3	2434874	-			NotPub	AE104_E		0		0	

CusA

Element	start	strand	stop	length	replicon	locus tag	gene	MEAN_C0	SDEV_C0	MEAN_CM	SDEV_CM
**TSS_1715990+3**	1715990	+			Pub	s		7922	417	13076	1725
NPKM_5030	1716010	+	1716412	402	CP000353	Rmet_5030	*cusD*	291.0	37.0	494.67	48.01
NPKM_5031	1716484	+	1717786	1302	CP000353	Rmet_5031	*cusC*	1.7	0.6	23.33	1.53
STP_AST_1724302-3	1717508	-	1717508	6794	CP000353			35.00	5.0		
**STP_AST_1720089-3**	**1716631**	**-**	**1716631**	**3458**	**CP000353**					**4.7**	**0.6**
NPKM_5032	1717782	+	1719300	1518	CP000353	Rmet_5032	*cusB*	2.0	0.0	24.33	2.52
NPKM_5033	1719296	+	1722464	3168	CP000353	Rmet_5033	*cusA*	1.7	0.6	23.67	1.53
**AST_1720089-3**	**1720089**	**-**	**1716631**	**3458**	**CP000353**			**4.7**	**0.6**		
**STP_AST_1724326-3**	**1720124**	**-**	**1720124**	**4202**	**CP000353**					**66.7**	**7.1**
RT-PCR_cusA	1722137		1722263	126							
NPKM_5034	1722460	+	1722832	372	CP000353	Rmet_5034	*cusF*	2.3	0.6	49.67	14.74
3UTR_5034	1722833	+	1722892	59	CP000353	Rmet_5034	*cusF*	4.0	1.0		
NPKM_6707	1722965	+	1723169	204	CP000353	Rmet_6707		38.7	12.9		
3UTR_6707	1723170	+	1724969	1799	CP000353	Rmet_6707		10.3	1.2		
NPKM_5035	1722953	-	1723172	219	CP000353	Rmet_5035		411.3	56.9	507.00	32.70
NPKM_5036	1723199	-	1723433	234	CP000353	Rmet_5036		179.0	15.7	308.00	31.51
TSS_1723428-3	1723428	-			NotPub	CH34_M		28	4	41	5
TSS_1723457-3	1723457	-			Pub	*no*		5211	481	12446	1480
TSS_1723500-3	1723500	-			NotPub	CH34_M		22	7	32	8
NPKM_5037	1723646	-	1724087	441	CP000353	Rmet_5037		27.3	3.2	25.00	2.00
TSS_1724117-3	1724117	-			Pub	m		1061	105	581	127
NPKM_5038	1724188	-	1725235	1047	CP000353	Rmet_5038	*bug*	3.0	0.0	2.33	1.15
AST_1724302-3	1724302	-	1717508	6794	CP000353			35.00	5.0		
**AST_1724326-3**	**1724326**	**-**	**1720124**	**4202**	**CP000353**					**66.7**	**7.1**
TSS_1727041-3	1727041	-			NotPub	CH34_E		33	22	0	

The *cus* and *cop*$_2$ determinants. The *cus* and *cop*$_2$ determinants were analyzed in more detail. The structural genes of the *cop*$_2$ determinant were not expressed in unchallenged CH34 and AE104 cells, nor were those of *copS*$_2$ and that of *copR*$_2$ was around the threshold of NPKM = 10 (Suppl. Fig. S4). Challenging the cells with metals resulted in an up-regulation of these genes in both strains, in the plasmid-free strain AE104 to higher levels compared to the plasmid-containing wildtype strain, CH34. Moreover, expression of some genes neighboring *cop*$_2$ was also up-regulated. Downstream of *copD*$_2$ on the other DNA strand and transcribed in the direction towards *copD*$_2$ were genes for a LysR-type regulator Rmet_5666 and a small membrane protein Rmet_5667. Downstream of *copS*$_2$ were the genes of the recessive metal-resistance determinant *nim*, which encodes an RND-driven trans-envelope system but with a gene for the RND protein NimA being interrupted by a transposon insertion. Downstream of *nim* on the same DNA strand were three additional genes with one of them (Rmet_5676) encoding an ArsR-type regulator (Suppl. Fig. S4). Expression of this *nim* region was up-regulated in metal-challenged CH34 cells and was further up-regulated in metal-challenged AE104 cells. In strain AE104, this resulted in a large transcript. It was initiated at the truncated *nimA*$_2$ gene, spanned *nimC* and three additional genes, including that for Rmet_5676, and became antisense to *copR*$_2$*S*$_2$ transcripts with a lower abundance. The transcript continued with a higher abundance as sense transcript for *copA*$_2$*B*$_2$*C*$_2$*D*$_2$ and was finally antisense again for the two genes downstream of *copD*$_2$ (Suppl. Fig. S4D). In metal-challenged cells, this resulted in a complex interaction of this transcript with transcripts from the other DNA strand, especially the *copR*$_2$*S*$_2$ transcript and another one that extended from *Rmet_5667* as antisense transcript into *copD*$_2$.

In unchallenged CH34 and AE104 cells, the *cus* genes were also not expressed (Suppl. Fig. S5), with the exception of *cusD*. All *cus* genes were up-regulated in metal-challenged CH34 cells but even more so in metal-challenged AE104 genes (5- to 7-fold compared to metal-challenged CH34 cells, Table 5). TraV Mac annotated antisense transcripts that started and stopped at different positions. The antisense abundance at the single base pairs of *cus* differed only to a small extent, which nevertheless resulted in different start and stop positions of the annotated transcripts, and subsequently to different NPKM values of these transcripts (Suppl. Fig. S5, blue boxes). A RpoD-dependent promoter was annotated upstream of *cusD* (18) for unchallenged cells of strain CH34, while the promoter for the antisense transcript was not associated with *rpoD*. Transcript abundance vanished just downstream of *cusD* in non-challenged cells but continued in metal-stressed cells (Suppl. Fig. S5), indicating an antitermination event or an internal promoter between *cusD* and *cusC*.

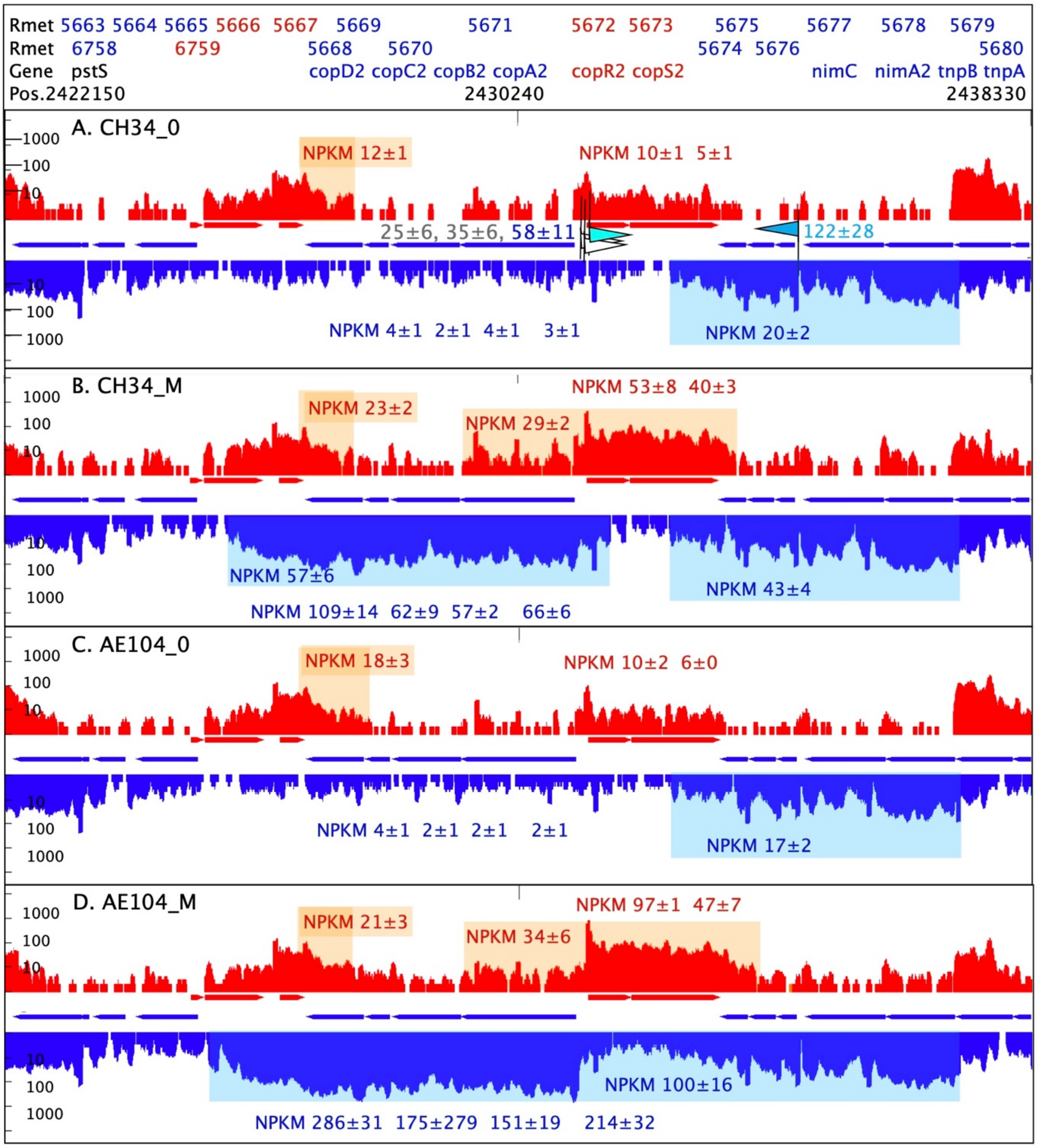

Supplementary Figure S4. Map of the *cop₂* metal resistance determinants in *C. metallidurans* strain CH34. This map summarizes the data from the RNASeq experiment. The map shows the indicated determinant with NPKM values on one DNA strand (red) or the other direction of transcription (blue) from triple determinations with deviations. The transcript abundances are from one of these experiments. The header gives the position on the chromid, the Rmet locus and gene names. TSS (flags) were only obtained for unchallenged CH34 cells (18) and are indicated with the corresponding TSS score (not: RpoD score), white for scores < 50 with no promoter consensus motifs indicated, blue shades for promoters not associated to the RpoD model. Shaded areas are annotated antisense transcript regions with their NPKM values. Panel A, unchallenged CH34 cells; Panel B, CH34 with MultiTox metal mix; Panel C, unchallenged AE104 cells; Panel D, AE104 with MultiTox metal mix.

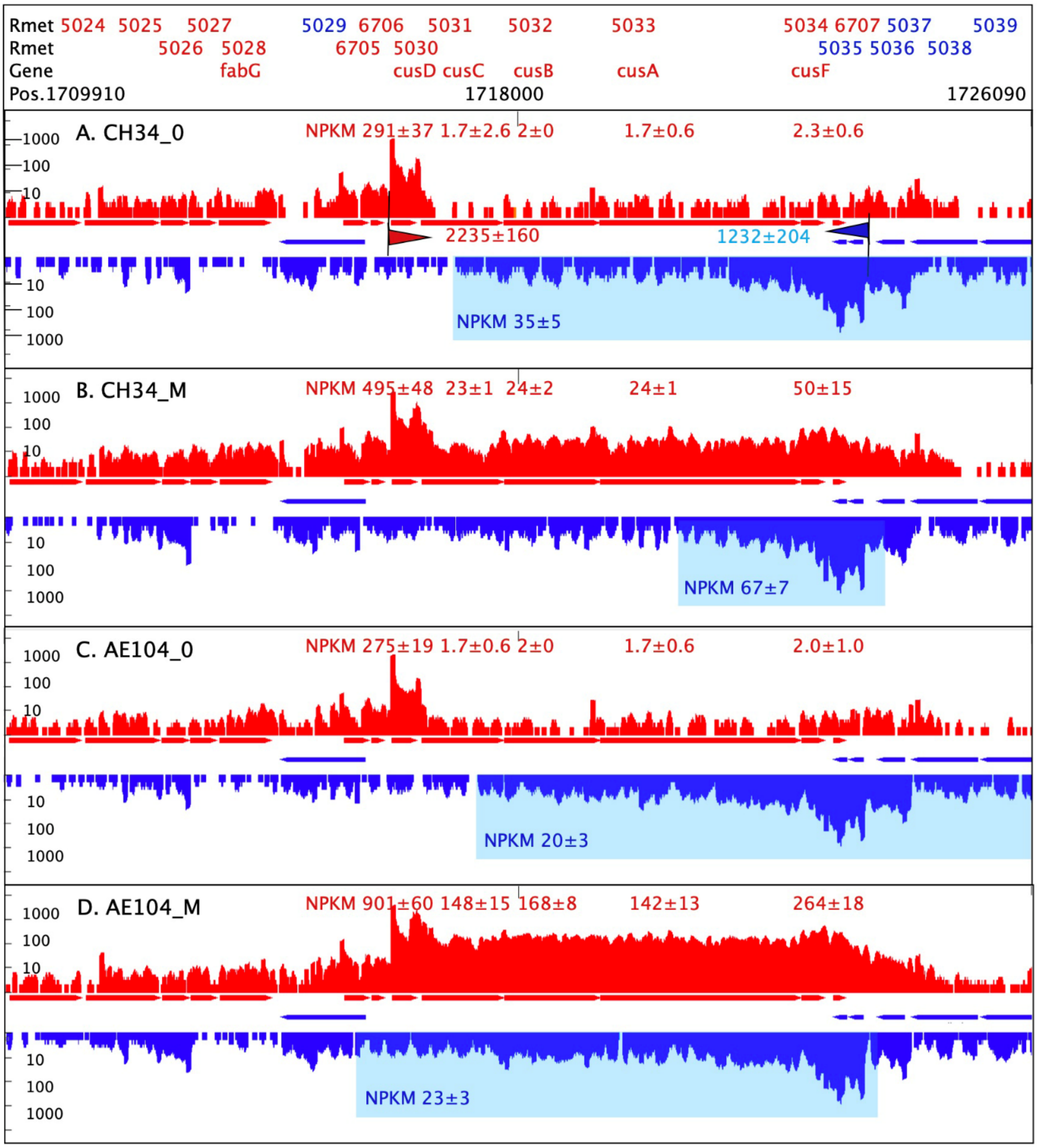

Supplementary Figure S5. Map of the *cus* metal resistance determinants in *C. metallidurans* strain CH34. This map summarizes the data from the RNASeq experiment. The map shows the indicated determinant with NPKM values on one DNA strand (red) or the other direction of transcription (blue) from triple determinations with deviations. The transcript abundances are from one of these experiments. The header gives the position on the chromid, the Rmet locus and gene names. TSS (flags) were only obtained for unchallenged CH34 cells (18) and are indicated with the corresponding TSS score (not: RpoD score), red shades for strong (>1000, red) RpoD promoters, blue shades for strong ones not associated to the RpoD model. Shaded areas are annotated antisense transcript regions with their NPKM values. Panel A, unchallenged CH34 cells; Panel B, CH34 with MultiTox metal mix; Panel C, unchallenged AE104 cells; Panel D, AE104 with MultiTox metal mix.

Supplementary Table S6. Primers used for RT-PCR-based amplification of asRNAs

Name	Sequence
cnrH(6207) 1126 up	CCC TGA AAC CGA TGT GGA
cnrH(6207) 1234 down	CGC ATC TTG TTC AGC CCA
czcP(5970) 3237 up	CAC TGG AAA CGG CGG ATG
czcP(5970) 3357 down	GCA GAA GGA ACG CCA CGA
zniR(5323) 1060 up	GAC CGC TAC GCT CAT CGC
zniR(5323) 1169 down	GCC CAC ATC GCA CAG GAC
copN(6109) 1338 up	TCG CTG TCG TGC TAT CGC
copN(6109) 1480 down	CCC TAC TTC GCC CAC TCG
cusA(5033) 3841 up	TCT CAA GCA CGC CTG GGA
cusA(5033) 3950 down	CAG GAT GAC GGC GAC GGT
copR2(5672) 1069 up	CGG CTT CAT CGT GGA CCT
copR2(5672) 1222 down	GCC GTC AGG AAC AGC ACC

Literature of the Supplement

1. Dietrich S, Wiegand S, Liesegang H. 2014. TraV: a genome context sensitive transcriptome browser. PLoS One 9:e93677.
2. Baker BJ, Comolli LR, Dick GJ, Hauser LJ, Hyatt D, Dill BD, Land ML, Verberkmoes NC, Hettich RL, Banfield JF. 2010. Enigmatic, ultrasmall, uncultivated Archaea. Proc Natl Acad Sci U S A 107:8806-8811.
3. Große C, Poehlein A, Blank K, Schwarzenberger C, Schleuder G, Herzberg M, Nies DH. 2019. The third pillar of metal homeostasis in *Cupriavidus metallidurans* CH34: Preferences are controlled by extracytoplasmic functions sigma factors. Metallomics 11:291-316.
4. Van Houdt R, Monchy S, Leys N, Mergeay M. 2009. New mobile genetic elements in *Cupriavidus metallidurans* CH34, their possible roles and occurrence in other bacteria. Antonie Van Leeuwenhoek 96:205-226.
5. Van Houdt R, Monsieurs P, Mijnendonckx K, Provoost A, Janssen A, Mergeay M, Leys N. 2012. Variation in genomic islands contribute to genome plasticity in *Cupriavidus metallidurans*. BMC Genomics 13:111.
6. Ray-Soni A, Bellecourt MJ, Landick R. 2016. Mechanisms of bacterial transcription termination: all good things must end. Annu Rev Biochem 85:319-347.
7. Molodtsov V, Wang C, Firlar E, Kaelber JT, Ebright RH. 2023. Structural basis of Rho-dependent transcription termination. Nature 614:367-374.
8. Mitra P, Ghosh G, Hafeezunnisa M, Sen R. 2017. Rho Protein: roles and mechanisms. Annu Rev Microbiol 71:687-709.
9. Di Salvo M, Puccio S, Peano C, Lacour S, Alifano P. 2019. RhoTermPredict: an algorithm for predicting Rho-dependent transcription terminators based on *Escherichia coli*, *Bacillus subtilis* and *Salmonella enterica* databases. BMC Bioinformatics 20:117.
10. Scherer J, Nies DH. 2009. CzcP is a novel efflux system contributing to transition metal resistance in *Cupriavidus metallidurans* CH34. Mol Microbiol 73:601-621.
11. Legatzki A, Anton A, Grass G, Rensing C, Nies DH. 2003. Interplay of the Czc-system and two P-type ATPases in conferring metal resistance to *Ralstonia metallidurans*. J Bacteriol 185:4354–4361.
12. Schulz V, Schmidt-Vogler C, Strohmeyer P, Weber S, Kleemann D, Nies DH, Herzberg M. 2021. Behind the shield of Czc: ZntR controls expression of the gene for the zinc-exporting P-type ATPase ZntA in *Cupriavidus metallidurans*. J Bacteriol 203:e00052-21.
13. Große C, Kohl T, Herzberg M, Nies DH. 2022. Loss of mobile genomic islands in metal resistant, hydrogen-oxidizing *Cupriavidus metallidurans*. Appl Environ Microbiol 88:e02048-21.
14. Rottet S, Iqbal S, Xifaras R, Singer MT, Scott C, Deplazes E, Callaghan R. 2023. Biochemical interactions between the Atm1-like transporter from *Novosphingobium aromaticivorans* and heavy metals. Arch Biochem Biophys 744.
15. Inlow K, Tenenbaum D, Friedman LJ, Kondev J, Gelles J. 2023. Recycling of bacterial RNA polymerase by the Swi2/Snf2 ATPase RapA. Proc Natl Acad Sci U S A 120:e2303849120.
16. Sukhodolets MV, Jin DJ. 2000. Interaction between RNA polymerase and RapA, a bacterial homolog of the SWI/SNF protein family. J Biol Chem 275:22090-22097.
17. Mergeay M, Nies D, Schlegel HG, Gerits J, Charles P, van Gijsegem F. 1985. *Alcaligenes eutrophus* CH34 is a facultative chemolithotroph with plasmid-bound resistance to heavy metals. J Bacteriol 162:328-334.

18. Große C, Grau J, Große I, Nies DH. 2022. Importance of RpoD- and non-RpoD-dependent expression of horizontally acquired genes in *Cupriavidus metallidurans*. Microbiol Spectr 10: 10.1128/spectrum.00121-22.
